# Mathematical Model Predicts Effective Strategies to Inhibit VEGF-eNOS Signaling

**DOI:** 10.3390/jcm9051255

**Published:** 2020-04-26

**Authors:** Qianhui Wu, Stacey D. Finley

**Affiliations:** 1 Department of Biomedical Engineering, University of Southern California, Los Angeles, CA 90089, USA; qianhuiw@usc.edu; 2 Department of Biomedical Engineering, Mork Family Department of Chemical Engineering and Materials Science, and Department of Biological Sciences, University of Southern California, Los Angeles, CA 90089, USA

**Keywords:** tumor angiogenesis, Thrombospondin-1, differential equation modeling, cell signaling

## Abstract

The endothelial nitric oxide synthase (eNOS) signaling pathway in endothelial cells has multiple physiological significances. It produces nitric oxide (NO), an important vasodilator, and enables a long-term proliferative response, contributing to angiogenesis. This signaling pathway is mediated by vascular endothelial growth factor (VEGF), a pro-angiogenic species that is often targeted to inhibit tumor angiogenesis. However, inhibiting VEGF-mediated eNOS signaling can lead to complications such as hypertension. Therefore, it is important to understand the dynamics of eNOS signaling in the context of angiogenesis inhibitors. Thrombospondin-1 (TSP1) is an important angiogenic inhibitor that, through interaction with its receptor CD47, has been shown to redundantly inhibit eNOS signaling. However, the exact mechanisms of TSP1′s inhibitory effects on this pathway remain unclear. To address this knowledge gap, we established a molecular-detailed mechanistic model to describe VEGF-mediated eNOS signaling, and we used the model to identify the potential intracellular targets of TSP1. In addition, we applied the predictive model to investigate the effects of several approaches to selectively target eNOS signaling in cells experiencing high VEGF levels present in the tumor microenvironment. This work generates insights for pharmacologic targets and therapeutic strategies to inhibit tumor angiogenesis signaling while avoiding potential side effects in normal vasoregulation.

## 1. Introduction

### 1.1. Overview of Tumor Angiogenesis and Its Importance in Tumor Progression

Angiogenesis, the formation of new blood capillaries from pre-existing vessels, plays a critical role in tumor progression [1]. For a solid tumor to expand beyond millimeters in size, it must promote new blood vessel formation to achieve sustained blood supply [1,2]. The process of angiogenesis is driven by a number of pro-angiogenic factors which promote several endothelial cell (EC) functions that contribute to new blood vessel formation. The resulting new vessels carry oxygen and nutrients to the tumor, allowing the tumor to enlarge, and also providing a path for the cancer cells to metastasize [1]. In fact, a meta-analysis revealed that high intra-tumoral microvessel density (MVD) is a significant predictor of poor survival for breast cancer patients [3].

### 1.2. VEGF-eNOS Signaling and Its Importance

**Figure 1:**
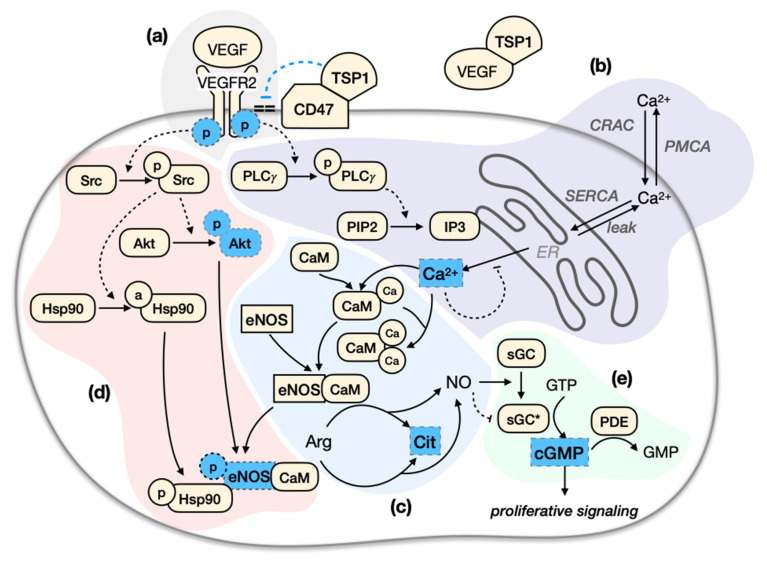
Model Schematic. (**a**) Receptor module. The signaling pathway begins with VEGF binding to receptor R2. Ligated R2 undergoes autophosphorylation and triggers the phosphorylation of Src and PLCγ. (**b**) Calcium module. Active PLCγ converts PIP_2_ to IP_3_ through hydrolysis. IP_3_ binds to its receptor on the ER membrane and induces Ca^2+^ release into the cytosol. The ER store Ca^2+^ depletion triggers further Ca^2+^ entry through the CRAC channel which is quickly balanced by several homeostatic mechanisms. (**c**) eNOS module. Ca^2+^ binds to and activates CaM, which in turn activates eNOS. eNOS converts its substrate arginine to citrulline, producing NO. (**d**) Src/Akt/Hsp90 module. Active Src activates Akt and the chaperon protein Hsp90. Hsp90 facilitates Akt association with eNOS, which results in eNOS phosphorylation. The binding of eNOS with Hsp90 and phosphorylation of eNOS both enhance eNOS activity. (**e**) sGC module. NO triggers cGMP synthesis from GTP. cGMP is degraded by PDE. TSP1 sequesters VEGF in the extracellular space and binds to its own receptor CD47 on the EC membrane, disrupting the coupling of CD47 and R2. Furthermore, TSP1 has been shown to reduce VEGF-induced activation of R2, Akt, and eNOS, agonist-stimulated Ca^2+^ increase, basal eNOS activity, and basal or VEGF/agonist-induced cGMP production. The signaling species observed to be affected by TSP1 are highlighted in blue. Shading indicates the relative location of reactions in the network. Light red: Src/Akt/Hsp90; light purple: Ca^2+^ system; light blue: eNOS activity; light green: sGC activity. Abbreviations: Akt, protein kinase B; CaM, Calmodulin; cGMP, Cyclic Guanosine Monophosphate; Cit, Citrulline; CRAC, Calcium Release-Activated Channels; eNOS, Endothelial Nitric Oxide Synthase; EC, Endothelial Cell; ER, Endoplasmic Reticulum; GMP, Guanosine Monophosphate; GTP, Guanosine Triphosphate; Hsp90, Heat Shock Protein 90; IP_3_, Inositol 1,4,5-Trisphosphate; PDE, Phosphodiesterase; PLCγ, Phospholipase C, Gamma; PIP_2_, Phosphatidylinositol 4,5-Bisphosphate; PMCA, Plasma Membrane Ca^2+^ Atpase; NO, Nitric Oxide; SERCA, Sarco/Endoplasmic Reticulum Ca^2+^-Atpase; sGC, Soluble Guanylate Cyclase; sGC *, activated Soluble Guanylate Cyclase; Src, proto-oncogene tyrosine kinase Src; TSP1, Thrombospondin-1; VEGF, Vascular Endothelial Growth Factor; VEGFR2 or R2, Vascular Endothelial Growth Factor Receptor 2.

One of the most well studied pro-angiogenic factors is vascular endothelial growth factor (VEGF), which is often upregulated in the tumor microenvironment [4,5,6,7]. VEGF-mediated signal transduction through its principal receptor VEGFR2 (herein abbreviated as R2) induces biological responses required for angiogenesis, including EC proliferation, survival, migration, and enhanced vascular permeability [8,9,10]. VEGF signaling through R2 promotes the endothelial nitric oxide synthase (eNOS) activity through multiple mechanisms [11,12,13,14] (Figure 1). eNOS produces nitric oxide (NO), an important vasodilator that rapidly diffuses throughout the endothelium. NO activates the enzyme soluble guanylate cyclase (sGC) to produce cyclic guanosine monophosphate (cGMP) [13,15]. This particular part of the eNOS signaling pathway contributes to both an acute vasodilating effect in the neighboring vascular smooth muscle cells (VSMCs) [8,11,16] and the long-term angiogenic functions of ECs, such as proliferation [11,14,17]. Given the biological significance of VEGF-mediated eNOS signaling, it is important to understand the dynamics of this multi-faceted pathway and how it responds to angiogenic factors.

As tumor progression strongly depends on angiogenesis, in the past decades researchers have developed anti-angiogenic therapies to inhibit tumor angiogenesis [18,19,20]. Many of these angiogenic inhibitors target VEGF and its receptor R2; however, they have only achieved limited success across a wide range of patients [19,20,21,22,23,24,25,26,27,28]. Therapeutic responses to VEGF inhibitors may be limited by parallel activation of the downstream pathways by additional pro-angiogenic factors. In addition, inhibiting R2 signaling can reduce the level of endothelium NO available for the paracrine regulation of VSMCs and platelets [29]. In fact, hypertensive and pro-thrombotic activities are frequent side effects of therapeutic angiogenic inhibitors. Furthermore, because the aberrant and leaky tumor vasculature is less responsive to vasoactive agents than healthy tissue [6,30], a vasodilator used to counteract hypertension can indirectly decrease blood flow through the tumor. This can further cause challenges in drug delivery to the targeted tumor location.

### 1.3. Thrombospondin-1 (TSP1) and Its Inhibitory Functions

Physiologically, angiogenesis is controlled by a balance between the angiogenic promotors and inhibitors [2]. The expression levels of both pro- and anti-angiogenic factors are often dysregulated in the tumor microenvironment in favor of angiogenesis. For example, the matricellular glycoprotein TSP1 is a potent endogenous angiogenic inhibitor, and its expression is inversely correlated with the malignant progression of several types of cancer [31,32,33]. TSP1 regulates the bioavailability and activity of VEGF via several mechanisms. TSP1 can directly bind and sequester VEGF in the extracellular space [34,35]. TSP1 also antagonizes R2 signaling through its own receptors, CD36 and CD47. At physiological (picomolar) concentrations, TSP1 binds to receptor CD47 on ECs and redundantly inhibits eNOS signaling [36,37]. At the nanomolar concentration, TSP1 blocks myristate uptake and induces EC apoptosis via receptor CD36 [38,39,40,41].

Due to TSP1′s potency and redundancy in inhibiting angiogenic signaling, the use of TSP1 and its mimetics in cancer therapy may be more advantageous than anti-VEGF treatments. Several CD47 antibodies are currently being investigated in preclinical studies or Phase I & II clinical trials [42,43]; however, rather than the anti-angiogenic effect, these mimetics are sought after to overcome immune evasion of the tumor cells expressing CD47 [44,45]. Meanwhile, the exact intracellular mechanisms of TSP1′s angiogenesis-inhibiting functions remain unknown, and the relative importance of its multiple signaling pathways mediated by receptors CD36 and CD47 are not quantitatively understood.

### 1.4. Need for Computational Modeling and Systems Biology Approaches

With the incomplete understanding of TSP1′s role in angiogenic regulation intracellularly, it is important to incorporate the accumulated knowledge and systematically study its inhibitory effects on angiogenic signaling. A computational systems biology approach allows for understanding such complex biological processes. Furthermore, by taking advantage of mathematical modeling, it is possible to predict optimal treatment strategies and identify biomarkers for patient screening. Thus, modeling is a powerful tool to efficiently generate testable hypotheses, which can reduce the cost of extensive “wet lab” and preclinical studies.

The vast majority of such modeling efforts have focused on the pro-angiogenic signaling networks. For example, we and others have developed models in the context of angiogenesis on various scales: intracellular signaling [46,47,48,49,50,51,52,53,54], cell and tissue level [55,56,57,58,59], and whole-body compartment models [60,61,62,63,64,65]. Previously, we have established a model of TSP1′s apoptotic signaling through receptor CD36, predicting several treatment strategies that may enhance the angiogenic inhibition in tumor [47]. However, this work provided quantitative insight into only one pathway by which TSP1 exerts its anti-angiogenic effect. Meanwhile, TSP1′s effect through its receptor CD47 remains understudied. Recently, Bazzazi et al. developed a mechanistic model to demonstrate how TSP1 may inhibit VEGF/R2 signaling via receptor CD47 [50]; however, their study focused on TSP1′s mechanisms at the receptor level, while the downstream, intracellular inhibitory mechanisms of TSP1 were not evaluated.

In the present study, we focused on uncovering the intracellular mechanisms of TSP1 through receptor CD47. We constructed a molecularly detailed, quantitative mechanistic model of VEGF- and TSP1-mediated eNOS signaling in ECs. We used the model to systematically explore the intracellular mechanisms of TSP1-CD47 signaling. Specifically, we focused on the effects of varying influential parameter values in the model without changing the model structure to hypothesize the mechanisms through which TSP1 inhibits the agonist-induced Ca^2+^ influx-plateau phase and the NO and cGMP generation in both basal and VEGF-stimulated conditions. Then, we used the model to identify effective strategies that selectively target ECs that experience higher VEGF level associated with the tumor microenvironment. Our results highlight several perturbations that achieve the experimentally observed TSP1-mediated inhibitory effects on the signaling outputs of interest. In addition, we showed that certain perturbations can achieve selective inhibition of the pro-angiogenic signaling when the VEGF level is high (similar to the concentrations present in tumor tissue), but not at normal VEGF levels. Thus, the model provides detailed insight regarding strategies to inhibit tumor angiogenesis while avoiding potential side effects in normal vasoregulation.

## 2. Methods

### 2.1. Mathematical Model

We constructed a rule-based model to describe the intracellular eNOS signaling in ECs mediated by VEGF and TSP1. The reaction rules are defined using the BioNetGen software. As depicted in Figure 1, the pro-angiogenic signaling pathway begins with VEGF binding to its main receptor, R2, on the endothelial cell membrane (Figure 1a). Ligated R2 undergoes autophosphorylation and triggers the phosphorylation of proto-oncogene tyrosine kinase Src (Src) and Phospholipase C, Gamma (PLCγ). Active PLCγ converts Phosphatidylinositol 4,5-Bisphosphate (PIP_2_) to Inositol 1,4,5-Trisphosphate (IP_3_) through hydrolysis. IP_3_ binds to its receptor on the endoplasmic reticulum (ER) membrane and induces Ca^2+^ release into the cytosol. The ER store Ca^2+^ depletion triggers further Ca^2+^ entry through the calcium release-activated channels (CRAC). This rapid increase in cytosolic Ca^2+^ is quickly balanced by several homeostatic mechanisms, including the re-sequestration of Ca^2+^ into the ER via the sarco/endoplasmic reticulum Ca^2+^-ATPase (SERCA), and the extrusion of Ca^2+^ from the cell via the plasma membrane Ca^2+^-ATPase (PMCA). The ER Ca^2+^ may also passively leak to the cytosol (Figure 1b). Ca^2+^ binds to and activates calmodulin (CaM), which in turn activates eNOS. eNOS converts its substrate Arginine to Citrulline, producing NO (Figure 1c). Src activates protein kinase B (Akt) and the chaperon protein, heat shock protein 90 (Hsp90). Hsp90 facilitates Akt association with eNOS, which results in eNOS phosphorylation. The binding of eNOS with Hsp90 and phosphorylation of eNOS both enhance eNOS activity (Figure 1d). NO rapidly diffuses throughout the endothelium and binds to sGC. Active sGC synthesizes cGMP from guanosine triphosphate (GTP). cGMP binds to and activates phosphodiesterase (PDE), which degrades cGMP to GMP (Figure 1e). This eNOS signaling pathway is essential to VEGF-mediated EC proliferation. For TSP1′s known functions, we include that TSP1 binds to VEGF in the extracellular space and its own receptor CD47 on the EC membrane, disrupting the coupling of CD47 and R2. For details on the experimental evidence that supports the formulated biochemical reactions in this model, see Supplemental Text in the Appendix A, Appendix B, Appendix C, Appendix D and Appendix E. Excitingly, our model is an advance compared to previous modeling works [50,52] as it includes the set of interactions between Hsp90, CaM, Akt, and eNOS, as well as signaling species downstream of eNOS. We applied this detailed model to understand the anti-angiogenic effects of TSP1 and other targeted perturbations in conditions recapitulating the tumor microenvironment.

### 2.2. Model Implementation

We formulated the biochemical reaction network using a rule-based approach with the BioNetGen software [66]. The reactions are assumed to follow well-established mass-action or Michaelis-Menten kinetics. Based on the specified rules, BioNetGen produces a set of ordinary differential equations (ODEs) that describe the rate of change in each species’ concentration over time. We describe this process in more detail in the Appendix A, Appendix B, Appendix C, Appendix D and Appendix E. The model file from BioNetGen, the resulting SBML model file, and a list of ODEs generated are provided in Files S1–S3. Using the MATLAB (The MathWorks, Inc., Natick, MA, USA) ODE solver suitable for stiff problems, *ode15s*, we computed time courses of species concentrations. The concentrations of the signaling species were computed for the same times used in the experimental time-course data available. The model is comprised of 127 kinetic rate parameters, three geometric parameters, 160 total species, with 18 non-zero initial conditions.

### 2.3. Publication Selection and Data Extraction

We searched for publications on Pubmed and Google Scholar, and found papers through references and citations. For full model fitting, the criteria for data inclusion was: (1) in vitro studies performed using the Human Umbilical Vascular Endothelial Cell (HUVEC) cell line, (2) cells were only stimulated by VEGF or TSP1, and (3) datasets showed time-course measurements for species’ concentrations. We also included two datasets showing R2 dynamics upon stimulation with VEGF and treatment with cycloheximide to estimate receptor internalization rates.

We extracted experimental data from figures found in various published studies [67,68,69,70,71,72] using an online data extraction software, WebPlotDigitizer [73]. If the datasets were not already quantified, we used Western blot images shown in the published paper. The images were extracted using Image J software based on the density of the protein bands and were normalized to their respective controls [74]. For datasets with arbitrary units, we normalized the data points to the maximum within each dataset.

### 2.4. Sensitivity Analysis

We performed the extended Fourier Amplitude Sensitivity Test (eFAST) to guide parameter selection for both model training and perturbation simulations. This is a variance-based approach that identifies the model parameters that significantly influence the model outputs [75,76], which are the predicted species’ levels. In this method, the parameter space is sampled within a specific range around the baseline parameter values over a specified distribution. We allowed the parameters to vary two orders of magnitude to investigate the influence of each parameter over a wide range of values. The eFAST method computes a total sensitivity index (*S_ti_*), which quantifies the variance of the model output with respect to the variances of each input and covariances between combinations of inputs. The *S_ti_* is a measure of the global sensitivity, accounting for the correlations among multiple inputs. The individual sensitivity indices are normalized by the sum total in order to be compared. Furthermore, the resulting sensitivity indices for all parameters are compared to that of the random “dummy” variable, and only indices significantly different from the dummy variable index (*p* < 0.05) are reported. The eFAST method has been used extensively in our previous work [46,47,55,56,77]. The parameters with *S_ti_* values larger than a cutoff value of 0.2 were determined as influential.

### 2.5. Identifiability Analysis

Prior to parameter estimation, we performed a structural parameter identifiability analysis [78,79]. This analysis determines whether the calibration problem is well posed and identifies which parameters can be uniquely specified from the available data. In this method, pair-wise correlation coefficients between model parameters were calculated. Parameters that were locally identifiable had correlations with all other parameters between −0.9 and 0.9. Parameters that were not locally identifiable, termed a priori unidentifiable, had correlations of >0.9 or <−0.9 with at least one other parameter. When two parameters are highly correlated, thus unidentifiable, and their values are unknown, it is necessary to specify the value of one of the parameters (described in model parameterization below) and estimate the value of the other parameter rather than estimate both redundant parameters.

### 2.6. Model Parameterization

Initial parameter settings: We pursued model development in a modular fashion. We developed several sub-modules that can be constrained independently, as illustrated in Figure 1. As a starting point, we first set the unknown parameter values based on information from various sources, including experimental studies [71,80,81,82] and previously established computational models [46,50,55,56,83,84,85,86,87,88,89]. For CD47 receptor concentration, we obtained the geometric mean of the number of CD47 receptors on cultured human microvascular endothelial cells (HMVECs) experimentally quantified using flow cytometry. Since there is no quantitative data available regarding the receptor number for HUVECs, we made the assumption that CD47 expressed on HUVECs is at the same level as on HMVECs.

**Figure 2:**
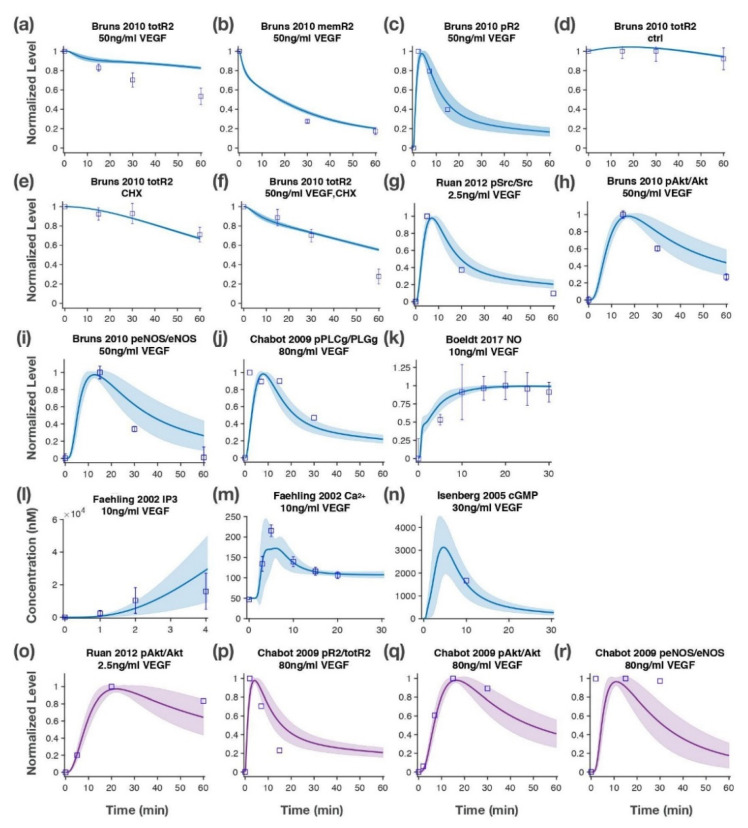
Model training and validation. The ODE model was trained to match in vitro experimental measurements of HUVECs for the activated species in the VEGF-mediated eNOS signaling pathway. Fitting results include model simulation compared to experimental datasets: (**a**) total R2 level; (**b**) membrane R2; (**c**) pR2 upon 50 ng/mL VEGF stimulation [67]; (**d**) total R2 in control condition [67]; (**e**) total R2 with cycloheximide treatment [67]; (**f**) total R2 with 50 ng/mL VEGF and cycloheximide treatment [67]; (**g**) pSrc with 2.5 ng/mL VEGF treatment [69]; (**h**) pAkt, (**i**) peNOS with 50 ng/mL VEGF treatment [67]; (**j**) pPLCγ with 80 ng/mL VEGF treatment [68]; (**k**) NO level with 10 ng/mL VEGF treatment [70]; concentration of (**l**) IP_3_ and (**m**) cytosolic Ca^2+^ with 10 ng/mL VEGF treatment [71]; (**n**) cGMP concentration with 30 ng/mL VEGF treatment [72]. (**o**–**r**) Several independent sets of data [68,69] were used to validate the model prediction. Solid line: mean of 19 best fits. Shaded area: standard deviation of 19 best fits. Squares: experimental data. Error bars: standard deviation of the experimental datasets. The normalized values are relative to the highest value for that species. ODE, ordinary differential equation; HUVECs, Human Umbilical Vascular Endothelial Cells; pR2, phosphorylated R2; pSrc, phosphorylated Src; pAkt, phosphorylated Akt; peNOS, phosphorylated eNOS; pPLCγ, phosphorylated PLCγ; totR2, total R2; CHX, cycloheximide.

Model fitting: After model construction, we performed sensitivity analysis and identifiability analysis to identify the influential and identifiable parameters to be estimated. We fixed the unknown, unidentifiable parameters based on literature [90,91]. In the full model training, a total of 23 uncorrelated, influential parameters were estimated. We provide the details of the parameter estimation performed during model development in Supplemental Text in the Appendix A, Appendix B, Appendix C, Appendix D and Appendix E. Briefly, we use the least-squares nonlinear regression optimization algorithm *lsqnonlin* function in MATLAB to estimate the unknown parameters. The training data consisted of 14 sets of time-course measurements (a total of 58 datapoints) [67,68,69,70,71,72] (Figure 2a–n). Based on the parameter estimation, 19 sets of estimated parameters with the lowest errors from fitting were selected as the best fit. We report the distribution of these parameter values in Figure S1. The best fit parameter sets were validated using four datasets not used in fitting [68,69] (Figure 2o–r). A list of all model parameters and their sources, including from literature and from the model parameterization, are in Table S1.

### 2.7. Model Perturbations

**Figure 3:**
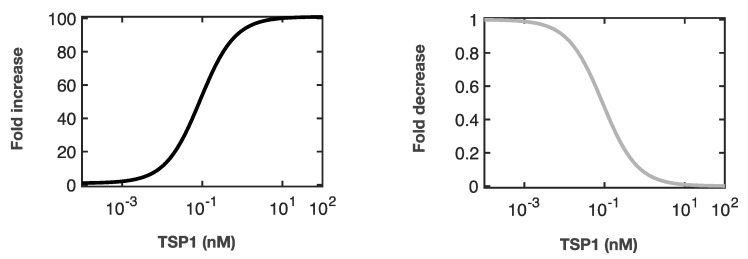
Hill functions of TSP1 concentrations. The model parameter value subject to variation is multiplied by *f_positive_* (**left panel**) or *f_negative_* (**right panel**) to increase or decrease its value, respectively, as a function of the TSP1 concentration.

To investigate the effects of perturbations on each individual parameter that is influential to the model outputs, we used the 19 best fit sets to simulate the model and altered the values of the parameters that were deemed to be influential in each respective condition based on eFAST analysis. We first modified the parameters as a function of TSP1 in each simulation. For these simulations, as the TSP1 level increases, the strengths of the perturbations increase. With these perturbations, we allowed the parameters to vary two orders of magnitude above or below the original value. We used the multiplicative factors *f_positive_* and *f_negative_* to scale up and scale down the value of each specific parameter, respectively. Parameters that would promote overall eNOS signaling were multiplied by *f_negative_*. Conversely, parameters that impede eNOS signaling were multiplied by *f_positive_*. We chose to utilize the Hill equation to implement the effect of TSP1, assuming a classic Michaelis-Menten input-output relationship [92]. Thus, the values of the multiplicative factors are given by a Hill function (Figure 3).

The model simulates the time-course of species’ concentrations. We quantified the fold-change of the area under curve (AUC) for Ca^2+^, NO, and cGMP for the first 30 min, normalized to that of the baseline model. We used NO level as an indicator of eNOS catalytic activity because it is an important vasoregulator. We reported the mean fold-change and the standard deviation across the 19 sets of simulations of each perturbation.

## 3. Results

### 3.1. Model Construction

We have constructed a model of the eNOS signaling pathway in ECs mediated by VEGF and TSP1 (Figure 1). Notably, we trained a detailed, novel module (termed “eNOS module”) to describe the eNOS catalytic activity differentially regulated by Hsp90 and Akt using independent experimental measurements. We adapted the calcium module describing the agonist-induced Ca^2+^ influx and homeostatic mechanisms from several studies [50,83,87], and the sGC module describing the NO/sGC-dependent cGMP synthesis is based on work by Halvey et al. [86]. We included the known function of TSP1 where it binds to VEGF and its receptor CD47, disrupting the coupling between CD47 and R2 [93].

We investigated the sensitivity of several model outputs (species concentration) to the variations in the model inputs (kinetic parameters and initial concentrations). Given a large number of model parameters, we organized the parameters into seven groups. Specifically, we estimated the effects of seven groups of model inputs: (1) receptor module, (2) Src/Akt/Hsp90 module, (3) calcium module, (4) eNOS module (activation), (5) eNOS module (catalytic activity), (6) sGC module, and (7) the initial concentrations of signaling species (Figure S2). The outputs for this sensitivity analysis, namely phosphorylated Akt and eNOS (pAkt and peNOS, respectively), Ca^2+^, NO, and cGMP, were chosen considering that these are the key signaling species throughout the signaling pathway, and model fitting would be conducted using experimental datasets for the dynamics of these species, in addition to data for R2 dynamics. Before model training, we performed the global sensitivity analysis eFAST to reveal the most influential parameters. The eFAST method enabled us to reduce the number of unknown parameters to estimate by fixing the non-influential parameters (see Methods). As a result of this analysis, we identified 34 influential parameters whose values were unknown. Using results from the parameter identifiability test, we further excluded 11 parameters from fitting as they were highly correlated with one or more of the remaining parameters to be estimated.

We trained the model by estimating the 23 unknown but influential model parameter values to match the model-simulated signaling dynamics to experimental data. The trained model recapitulated the experimentally observed signaling dynamics of key species throughout the pathway, such as phosphorylation of R2, phosphorylation of eNOS, NO, and cGMP (Figure 2a–n). Furthermore, the calibrated model generated predictions of signaling dynamics that were validated by four independent datasets not used in training [68,94] (Figure 2o–r). The distributions of all estimated parameter values were within one order of magnitude around their respective baseline values, as shown in Figure S1.

### 3.2. Model Dynamics with Physiological or Exogenous VEGF Levels

**Figure 4:**
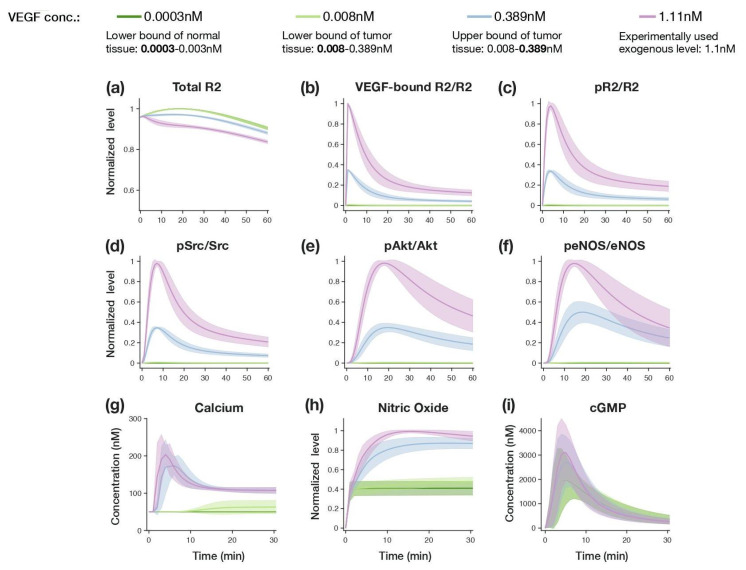
Signaling dynamics predicted by the model for various levels of VEGF stimulation. We compared the levels of (**a**) total R2, normalized species: (**b**) VEGF-bound R2/R2, (**c**) pR2/R2, (**d**) pSrc/Src, (**e**) pAkt/Akt, (**f**) peNOS/eNOS, (**g**) cytosolic Ca^2+^, (**h**) normalized NO, (**i**) cGMP with four different VEGF conditions selected from healthy tissue and tumor tissue measurements, and an experimentally used exogenous VEGF concentration. For (**a–f**,**h**), the species levels were normalized against the highest value across the four sets of simulations. For (**g**,**i**), concentrations of species are shown. Solid line: mean of model predictions using the 19 sets of best fit parameter values. Shaded area: standard deviation of model predictions within each simulation condition. The nomalized values are relative to the total amount of that species.

We first used the calibrated model to simulate the eNOS signaling dynamics stimulated with various VEGF concentrations taken from observed ranges, including concentrations at the lower bound of the range found in healthy tissue (0.0003 nM) [95,96], the lower and upper bounds of the range in tumor tissue (0.008 nM and 0.389 nM, respectively) ([97], and as compiled in [65]); and a concentration of exogenous VEGF concentration frequently used in experimental studies (1.1 nM; 30 ng/mL). Studying this wide range of VEGF stimulation levels allowed us to understand the level of signaling occurring in the ECs in different physiological and pathological conditions. We presented the signaling dynamics within 60 min (Figure 4) at the receptor level: total R2, VEGF-bound R2, and phosphorylated R2 (pR2); for relatively upstream through intermediate signaling: phosphorylated Src, Akt, and eNOS; and three major model outputs intermediate and downstream species: free cytosolic Ca^2+^, NO, and cGMP. With the exception for Ca^2+^ and cGMP, we report the relative species activation normalized to the highest level achieved across the four sets of simulations to make the comparison more direct.

The model simulation revealed that signaling species in the receptor module, Src/Akt/Hsp90 module, and eNOS module are more sensitive to various VEGF levels compared to the signaling species in the calcium module and sGC module. The model predicts that the lower concentration of VEGF from tumor tissue (light green, 0.008 nM VEGF), does not greatly promote eNOS signaling, in comparison to the response to the VEGF level present in healthy tissue (dark green, 0.0003 nM VEGF). On the other hand, the VEGF level present in tumors (blue, 0.389 nM VEGF) elicits a stronger signaling response that is similar to that produced by the exogenous VEGF level (pink, 1.1 nM VEGF). At these higher VEGF levels (0.389 nM and 1.1 nM), a transient increase can be observed in the levels of all the species. Compared to the signaling dynamics with 0.389 nM VEGF, the species activated in the receptor module and Src/Akt/Hsp90 module (e.g., pR2/R2, pSrc/Src, pAkt/Akt) are depleted more rapidly with 1.1 nM VEGF, whereas the Ca^2+^ influx magnitudes remain quite similar between the two cases. This could explain why there is little difference in the levels of downstream NO and cGMP for the two higher levels of VEGF stimulation (Figure 4g–i), even though the magnitude of activation is very different in the receptor module, Src/Akt/Hsp90 module, and eNOS module (Figure 4b–f, blue vs. purple lines). That is, signaling differences promoted by 0.389 and 1.1 nM VEGF are gradually lost as the signal propagates downstream. In addition, the desensitization mechanism of sGC to NO level included in our model (which is based on experimental evidence) also contributes to cGMP’s reduced sensitivity to changes in the VEGF level. Together, these results show that the downstream output cGMP is particularly robust to changes in the input level.

### 3.3. Simulated Effects of TSP1-Mediated Perturbations

The modeling framework allows us to better understand experimental observations that show TSP1 affects eNOS signaling in endothelial cells in different ways. Kaur et al. identified CD47 as an R2-associated protein and showed that CD47 ligation by TSP1 abolishes the constitutive coupling between CD47 and R2 [93]. Bauer et al. showed that TSP1 inhibits basal HUVEC eNOS catalytic activity and agonist-induced Ca^2+^ influx [36]. Isenberg et al. showed that TSP1 inhibits cGMP synthesis in HUVECs both in the basal condition and when stimulated with VEGF or NO donor [72]. Together, these phenomena suggest that TSP1 has multiple inhibition targets in endothelial cell eNOS signaling under the basal and VEGF-stimulated conditions. TSP1′s direct effect at the receptor level has previously been studied in detail using computational modeling; however, the authors focused only on enhanced R2 degradation or dephosphorylation by TSP1 [50]. Thus, it remains unclear how TSP1 inhibits several downstream effectors of eNOS signaling independent of R2 inhibition.

In order to integrate existing knowledge of the eNOS signaling pathway and mechanistically explain the experimentally observed effects of TSP1 under various VEGF-stimulated conditions, we used this model to identify possible intracellular targets of TSP1. Since it is not known how or if TSP1 interacts with the intracellular species, we simulated its effect by directly varying kinetic rate parameters and species’ initial concentrations. Specifically, we simulated and quantified the effects of perturbations to individual reactions in the signaling network and compared them with the experimentally observed TSP1 effects. We chose to use the levels of intracellular Ca^2+^, NO, and cGMP as the main model outputs, as they correspond to the experimental measurements from the TSP1 inhibition experimental studies described above.

To select the perturbation targets, we used the global sensitivity analysis on the calibrated model with the best-fit parameter set to quantify the sensitivity of the three outputs to variations in the model inputs. We define a parameter as influential if its global sensitivity index (*S_ti_*) value is no less than 0.1. In total, in the basal condition (0.0003 nM), 41 parameters are identified as influential (Figure S3a), whereas with a higher VEGF level (0.389 nM), 76 parameters are influential (Figure S3b).

First, we simulated various perturbations in the basal condition by altering (increasing or decreasing) the 41 individual parameters as a function of the TSP1 concentration. Specifically, we used Hill functions to characterize how the parameter value changed depending on the TSP1 level (Figure 3). The TSP1 concentrations used here correspond to those used experimentally in the study by Bauer et al., ranging from 0.00022 nM to 2.2 nM of TSP1. At 2.2 nM TSP1, the fold-change that is applied to alter each parameter value is approximately 100-fold, with the assumption that at such levels of TSP1, its inhibitory action achieves near maximal strength. We quantify the area under curve (AUC) of the time-courses of the three model outputs for the first 30 min, normalized to the AUC for the control condition without any perturbation.

**Figure 5:**
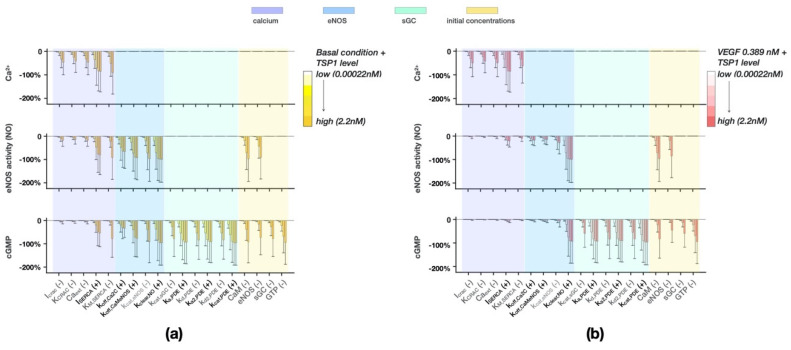
(**a**) Perturbations that are significantly effective in reducing the model output area under curve (AUC) levels by at least 20% compared to the baseline level. VEGF concentration is 0.0003 nM. (**b**) Effects of the same set of perturbations when VEGF is 0.389 nM. Bars: mean percent difference between perturbations and baseline level using the 19 sets of best fit parameter values with six TSP1 levels (0.0002–2.2 nM). Error bars: standard deviation of 19 sets of simulations. X-axis labels with (+) sign with black font: parameter was increased using the left panel Hill function in Figure 3; (-) sign with gray font: parameter was decreased using the right panel Hill function in Figure 3.

We show the perturbations that significantly (*p* < 0.05) reduced the level of any of the three quantified model outputs by at least 20% compared to the unperturbed condition (Figure 5a & Table S2). A total of 19 perturbations met the criteria, and therefore the corresponding mechanisms being perturbed are predicted candidates of TSP1′s intracellular targets of inhibition. To investigate whether these perturbations take effect when VEGF level is high, we also simulated these 19 perturbations with stimulation using the VEGF level found in tumor tissue, 0.389 nM (Figure 5b). Altogether, the results show that the model outputs have dose-dependence on TSP1. We further describe the results below.

For the basal condition, multiple perturbations in the calcium module and the eNOS module reduced both the eNOS catalytic activity level and cGMP (Figure 5a). Perturbing the Ca^2+^ influx mechanism CRAC, governed by parameters *I_CRAC_* and *K_CRAC_*, and the extracellular Ca^2+^ level Ca_ext_, reduced the Ca^2+^ AUC but only minimally affected the downstream activity. Meanwhile, perturbing the Ca^2+^ re-sequestration mechanism governed by parameters *I_SERCA_* and *K_M,SERCA_* achieved strong inhibition effects on all the model outputs. As one might expect, increasing the dissociation rate of Ca^2+^ from the calmodulin C-terminus *k_off,Ca2C_*, the dissociation rate of calmodulin and eNOS *k_off,CaMeNOS_*, and decreasing the catalytic rate of basal eNOS *k_cat.eNOS_* all have a strong effect on both the eNOS catalytic activity and cGMP levels. However, the effects of all the above perturbations on eNOS catalytic activity and cGMP levels are reduced under higher VEGF stimulation (Figure 5b).

On the other hand, several perturbations take effect under both the low and high VEGF conditions. Increasing the clearance rate of NO *k_clear,NO_* resulted in reduced levels of eNOS catalytic activity and cGMP without affecting the Ca^2+^ level. Similar effects are predicted with lowered initial concentrations of CaM and eNOS. Meanwhile, perturbations in the sGC module, including the reduced catalytic rate of sGC (*k_cat,sGC_*), enhanced activation rates of PDE (*k_a,PDE_*, *k_a2,PDE_*), reduced deactivation rates of PDE (*k_d,PDE,_ k_d2,PDE_*), and enhanced PDE catalytic rate (*k_cat,PDE_*), reduced only the cGMP AUC without affecting the upstream eNOS catalytic activity. Similar to these perturbations, lowering the levels of sGC or GTP can strongly reduce cGMP level.

In summary, the simulated perturbations identify several parameters and initial conditions that, depending on the TSP1 concentration, can inhibit different parts of the eNOS signaling pathway. Experimental evidence shows that TSP1 reduces agonist-induced Ca^2+^, basal eNOS catalytic activity, and both basal- and VEGF or NO donor-induced cGMP synthesis [36,72]. Importantly, our model predictions reveal individual mechanisms that, when combined together, could explain these observed effects of TSP1. We present a detailed interpretation of these results in the Discussion section.

### 3.4. Effective Perturbations for High VEGF Condition

In addition to investigating TSP1′s inhibitory mechanisms, we applied the model to identify strategies that selectively target eNOS signaling in cells within the tumor microenvironment, which often exhibit higher levels of pro-angiogenic factors [98,99,100,101]. To do so, we first used the eFAST method to identify parameters that are influential when VEGF level is high (Figure S3b), but not in the basal VEGF condition (Figure S3a). We then simulated the effects of altering those parameters and predicted the responses of the three main model outputs. In this set of simulations, VEGF concentration was 0.389 nM and no TSP1 was given. The perturbation was simulated by scaling the respective parameter values by the same range of values (2- to 100-fold) as used in Figure 5.

**Figure 6:**
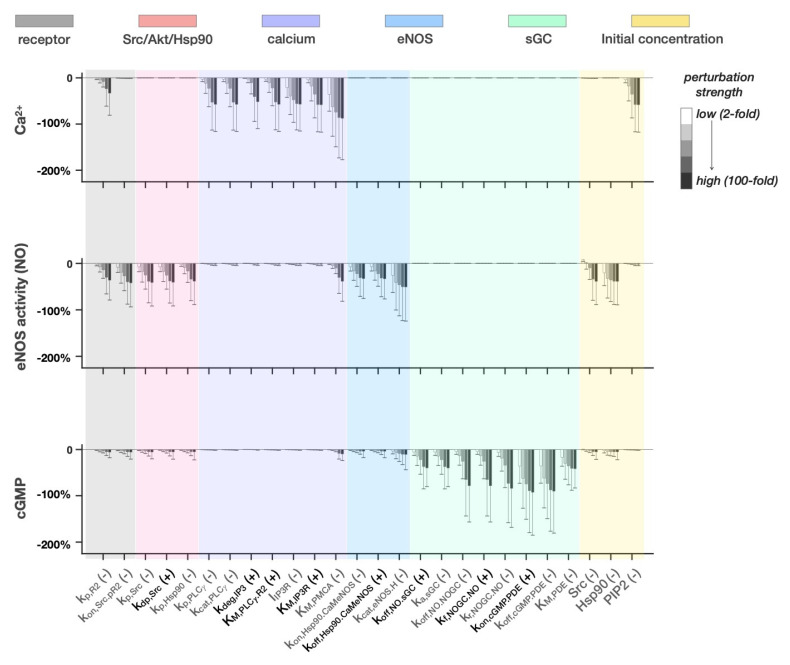
Effective perturbations in high VEGF condition. Perturbations shown are significantly effective in reducing the model output AUC levels by at least 10% compared to the baseline level. VEGF concentration is 0.389 nM and TSP1 concentration is 0 nM in all simulations. Bars: mean of model predictions using the 19 sets of best fit parameter values with six perturbation levels (scaling parameters by 2- to -100-fold). Error bars: standard deviation of 19 sets of simulations. Shaded areas: modules where the perturbed parameter takes effect. (+) sign with black font: parameter was increased using the left panel Hill function in Figure 3; (-) sign with gray font: parameter was decreased using the right panel Hill function in Figure 3.

From this set of simulations (Figure 6), we see that most of the perturbations to parameters in the receptor module and Src/Akt/Hsp90 module reduced eNOS catalytic activity by approximately 50%, yet minimally affected the cGMP level. A similar result is observed for the perturbations in the eNOS module. All of the effective perturbations in the calcium module are related to the PLCγ-dependent IP_3_-induced Ca^2+^ release mechanisms, controlled by parameters *k_p,PLC__γ_*, *k_cat,PLC__γ_*, *k_deg,IP3_*, *I_IP3R_*, *K_M,IP3R_*, with the exception of parameter *K_M,PMCA_*. These perturbations largely reduced the Ca^2+^ level but did not affect the downstream eNOS catalytic activity and cGMP. We found eight perturbations in the sGC module that inhibit the cGMP level. Among those eight parameters, varying the binding activity of NO with sGC’s distal and proximal binding sites (*k_off,NO.NOGC_*, *k_f,NOGC.NO_*, *k_r,NOGC.NO_*) and increasing PDE activation via its association with cGMP (*k_on,cGMP.PDE_*, *k_off,cGMP.PDE_*) most strongly reduced cGMP. In addition, reducing the expression levels of Src or Hsp90 inhibited the eNOS catalytic activity, and reducing PIP_2_ reduced Ca^2+^ level.

Altogether, the model simulations provide detailed insight of VEGF-mediated eNOS signaling and the effects of perturbing the network. The model reveals that the signaling response is sensitive to changing the VEGF levels in certain upstream modules, but this sensitivity is lost as the signal propagates downstream to cGMP synthesis. Using this model, we identified possible mechanisms of TSP1′s inhibitory function on the eNOS signaling pathway in both basal and VEGF-stimulated conditions. Furthermore, the model predicted alternative strategies to inhibit eNOS signaling, highlighting perturbations to the eNOS signaling network that only affect the cells experiencing a high-level VEGF-induced signaling response. Moreover, it is possible to inhibit distinct parts of the signaling pathway. That is, some perturbations will specifically inhibit upstream species (such as Ca^2+^), while not altering downstream species (such as cGMP), or vice versa.

## 4. Discussion

We have developed a molecular-detailed model of the intracellular eNOS signaling pathway in ECs, regulated by two important angiogenic factors: VEGF and TSP1. This model captures the experimentally observed VEGF-induced eNOS signaling dynamics quantitatively (Figure 4). This work is complementary to our previous work, including models that characterize the interactions of angiogenic factors present in the extracellular space with their cell-surface receptors [55,56,57], VEGF-mediated pro-angiogenic signaling through the mitogen-activated protein kinase (MAPK) pathway [46], and TSP1-mediated apoptotic pathway via receptor CD36 [47]. With this model, we are able to investigate the unknown intracellular mechanisms of TSP1 via receptor CD47 and generate insight to help address the problem of systemic hypertension elicited by anti-angiogenic drugs, novel contributions of our work.

This calibrated mechanistic model allows us to efficiently simulate and quantify the signaling dynamics of the eNOS pathway under various stimulation conditions. First, we take advantage of the model to predict the signaling dynamics in response to changes to VEGF levels. We show that the upstream signaling species are more sensitive to different levels of VEGF signaling, while downstream species are more robust to changes in the stimulant (Figure 3). This hypothesis that the model generated can be validated experimentally.

Next, we used the model to hypothesize the details of possible intracellular mechanisms of TSP1 that are currently unknown. These simulations are especially relevant in understanding TSP1′s multiple functions and their relative importance in angiogenic inhibition. Furthermore, as the tumor vasculature is typically comprised of highly proliferative ECs whereas the normal vasculature is quiescent, likely due to the difference in the growth factor levels in their respective environment, we used the model to generate predictions of effective strategies that selectively target only the ECs experiencing high VEGF levels observed in the tumor microenvironment. The model efficiently generates testable predictions for future experimental studies to validate. In the following sections, we discuss these model predictions in more detail.

### 4.1. Model Predicts Intracellular Target Mechanisms of TSP1

We use the calibrated mechanistic model to investigate potential intracellular targets of TSP1, and several predictions agree with the experimental observations. TSP1 has been shown to act through receptor CD47 to reduce the levels of several intracellular signaling species in the eNOS signaling pathway [36,72,93]; it is expected that TSP1 acts through specific mechanisms that produce these observed effects. As TSP1 inhibits eNOS signaling in both the basal condition and agonist-induced conditions, we present the effects of several perturbations that are shown to be influential in the basal condition (Figure 5a) and compare their effects to what happens when VEGF is present (Figure 5b). Note that these perturbations are in addition to two inhibitory functions of TSP1 at the receptor-ligand level: the sequestration of VEGF by TSP1 in the extracellular matrix and the disruption of CD47-R2 coupling once TSP1 binds to CD47.

An experimental study has shown that at the relatively upstream level, TSP1 reduced the sustained level of Ca^2+^ stimulated by ionomycin [36], which acts on internal Ca^2+^ stores [102]. The sustained Ca^2+^ phase is thought to be maintained by an influx through the CRAC channel and balanced by the other homeostatic mechanisms. Our model predicts several mechanisms through which Ca^2+^ influx upon VEGF stimulation can be reduced. Specifically, these mechanisms include perturbing of the CRAC mechanism, decreasing extracellular Ca^2+^ level, enhancing cytosolic Ca^2+^ re-sequestration to the ER (Figure 5b), or sensitizing the PMCA pump which extrudes cellular Ca^2+^ (Figure 5a,b).

TSP1 inhibits basal eNOS catalytic activity in endothelial cells [36]. In the basal condition, the model predicts that promoting the dissociation of Ca^2+^ from CaM, or CaM from eNOS, or decreasing the basal eNOS catalytic rate directly inhibits the basal eNOS catalytic activity (Figure 5a). These three perturbations take effect only in the basal condition and lose their inhibitory strengths when the VEGF level is high (Figure 5b), suggesting that TSP1 can inhibit basal eNOS catalytic activity via these mechanisms but may act through other mechanisms to reduce VEGF-induced eNOS catalytic activity. Additionally, the model predicts that perturbations to the SERCA mechanisms also reduce eNOS catalytic activity and subsequent cGMP synthesis; however, without experimental data it is difficult to know whether TSP1 disrupts Ca^2+^ homeostasis in quiescent ECs.

TSP1 inhibits basal and VEGF-stimulated cGMP synthesis [72]. The model predicts that increasing the NO clearance rate, reducing sGC activity, or enhancing PDE activity achieves cGMP inhibition in both the basal and VEGF-stimulated conditions (Figure 5). Comparing our results to the experimental findings, Isenberg et al. suggest that TSP1 may increase cellular metabolism of NO but does not selectively activate a PDE, which helps us exclude the PDE-related perturbations as TSP1′s function. A study has shown that TSP1 inhibits sGC activity induced by sGC activators besides NO [103]. This implies that the inhibition of sGC activation is not simply due to oxidation of the sGC heme, which agrees with our finding. Together, we have used the model to hypothesize that TSP1 enhances the clearance rate of NO and suppresses sGC activity independent of NO or PDE, in both the basal and VEGF-stimulated conditions. These model-generated hypotheses can be used to guide experimentation to further investigate TSP1′s effects.

### 4.2. Relevant Insights for Selectively Targeting VEGF-Stimulated Signaling

In addition to investigating the intracellular targets of TSP1, we explored alternative perturbation strategies that can affect VEGF-stimulated eNOS signaling. We sorted the effective strategies according to which aspect of the network they influence. Importantly, the perturbations that strongly reduce cGMP in this condition can serve as strategies to selectively inhibit eNOS signaling promoted by VEGF, potentially eliminating the hypertensive effects that occur from applying an anti-angiogenic drug that universally inhibits eNOS catalytic activity. Below, we discuss in detail these model predictions and what they contribute to the field of knowledge in terms of the following aspects: (1) predictions that have been pursued in experimental studies, (2) predictions that explain the mechanisms of agents being investigated and therefore complement existing and ongoing studies, and (3) predictions that reveal new strategies that have not yet been pursued.

#### 4.2.1. Predictions That Have Been Pursued in Experimental Studies

From the simulated perturbation results, we observe that the regulation of the eNOS catalytic activity is rather independent of the VEGF-induced Ca^2+^ response through PLCγ activation and IP_3_ generation, but heavily relies on the Akt-Hsp90 pathway. Along this axis, inhibition of the Src and Hsp90 chaperon protein activity prominently reduced eNOS catalytic activity but did not affect the cGMP level. In addition, the model predicts that lowering the levels of Src or Hsp90 also results in similar inhibitory effects (Figure 6). Clinically, both Src and Hsp90 inhibitors are being investigated as therapeutic agents to treat various types of cancer in clinical trials [43,104,105], although the focus lies in their functions in cancer cells. When considering this purpose, it is worth noting that these perturbations do not affect the basal eNOS catalytic activity and therefore should not induce harmful effects in vasoregulation for patients receiving these drugs.

On the other hand, individually blocking IP3R channel (*I_IP3R_* and *K_M,IP3R_*) or the CRAC flux (*I_crac_*), reduced VEGF-induced Ca^2+^ influx but did not affect NO or cGMP (Figure 5b, Figure 6), and similar results were observed when combining those perturbations (data not shown). This is likely because, as mentioned above, the eNOS catalytic activity is regulated by the presence of Akt and Hsp90, both of which enhance eNOS electron flow and sensitivity to CaM binding. In other words, the basal Ca^2+^ level is sufficient in supporting the activation of VEGF-induced eNOS catalytic activity without the external Ca^2+^ influx. Meanwhile, an experimental study showed that HUVECs pretreated with carboxyamidotriazole (CAI), an inhibitor of non-voltage operated Ca^2+^ channels (cancer.gov), showed a decrease in the cGMP in response to VEGF stimulation within two minutes [71]. However, it is possible that pretreatment with CAI disrupted the Ca^2+^ equilibrium, making direct comparison of the model simulations with the experimental results unsuitable. In fact, for some parameter sets, our model simulations suggest that the VEGF-stimulated eNOS catalytic activity can be inhibited by mechanisms that reduce the intracellular Ca^2+^ to below the basal level (50 nM) (data not shown).

The model predicts that in addition to TSP1′s possible mechanism of directly reducing the catalytic rate of sGC, several perturbations can reduce the VEGF-stimulated cGMP downstream of eNOS catalytic activity. These include enhancing the dissociation of NO from the distal heme activation site of sGC (increasing *k_off,NO.sGC_*) or reducing the activation rate of NO-bound sGC (decreasing *k_a,sGC_*) (Figure 6). Additionally, directly reducing the sGC level can inhibit cGMP in both the basal and VEGF-stimulated conditions (Figure 5). These results agree with experimental studies using the sGC inhibitors, 1H-(1,2,4) oxadiazolo (4,3-a) quinoxalin-1-one (ODQ) and NS2028, which are effective both in vitro and in vivo [106]. These inhibitors decrease sGC activity by oxidizing the heme cofactor in its regulatory heme-nitric oxide/oxygen binding (H-NOX) domain, potentially resulting in heme loss and prohibiting NO from binding to sGC.

#### 4.2.2. Predictions That Complement Existing/Ongoing Studies

The model predicts that although various perturbations to the Ca^2+^ homeostatic mechanisms can reduce the VEGF-induced Ca^2+^ response (Figure 5b, Figure 6), only perturbing the PMCA mechanism through sensitization (decreasing *K_M,PMCA_*) substantially reduced the eNOS catalytic activity. This finding is complementary to those from an experimental study [107], showing that substantially enhancing clearance of the intracellular Ca^2+^ by PMCA does inhibit eNOS catalytic activity. In the future, researchers can expand our model by including the binding event between PMCA and eNOS to investigate the specific mechanism and relative significance of PMCA’s negative regulatory role via its association with eNOS.

The model predicts that inhibiting the association of Hsp90 with CaM-bound eNOS (decreasing *k_on,Hsp90.CaMeNOS_* or increasing *k_off,Hsp90.CaMeNOS_*), or decreasing specifically the catalytic rate of Hsp90-bound eNOS can reduce the NO level, while still only minimally affecting the downstream cGMP level. In addition, directly reducing CaM availability or eNOS itself strongly reduced both eNOS catalytic activity and cGMP. Experimentally, there are a large number of existing compounds that inhibit eNOS catalytic activity through various mechanisms [108]: compounds that compete with the substrate arginine or the tetrahydrobiopterin (BH4) cofactor (not depicted in our model); inhibitors interacting directly with the heme to prevent eNOS dimerization (reducing the available eNOS level); and inhibitors that interact with CaM. Interestingly, our model predicts that in the VEGF-stimulated condition, individually altering the influential binding rates between Ca^2+^ and the two sites on CaM, between various forms of CaM and eNOS, or between various forms of eNOS with arginine did not significantly affect eNOS catalytic activity (data not shown). However, when we universally inhibit the binding events between Ca^2+^ with all sites on CaM, binding of CaM with all forms of eNOS, binding of all forms of eNOS with arginine, or catalytic activity of all forms of eNOS by 10-fold, these result in a strong reduction in the VEGF-stimulated eNOS catalytic activity and cGMP (Figure S4). Furthermore, these sets of universal perturbations also achieved maximal inhibition of eNOS catalytic activity and cGMP in the basal condition (data not shown), pointing to the potential side effect of the use of generic CaM or eNOS inhibiting compounds as they can affect the endothelium in both basal and high VEGF environments.

#### 4.2.3. Predictions That Reveal Strategies That Have Not Yet Been Pursued

Model predictions show that enhanced re-sequestration of intracellular Ca^2+^ (increasing *I_SERCA_* or decreasing *K_M,SERCA_*) reduces the intracellular Ca^2+^ levels in both basal and VEGF-stimulated conditions (Figure 5). It may be possible to implement this perturbation experimentally, as a study found that the upregulation of TMTC, a novel ER-resident adapter protein that associates with SERCA2B, could reduce the Ca^2+^ release from the ER [109], although the study was done with different cell types (human embryonic kidney HEK 293T and COS).

In addition to reducing NO-dependent sGC activation as a perturbation strategy, our model predicts that enhancing the desensitization of sGC via binding of NO on the proximal heme site (decreasing *k_off,NO.NOGC_*) and enhancing the dissociation of NO from the distal heme activation site on sGC (increasing *k_f,NOGC.NO_* or decreasing *k_r,NOGC.NO_*) (Figure 6) also achieves inhibition of cGMP, and only takes effect in the VEGF-stimulated condition. On the other hand, sGC downregulation takes effect in both the basal and VEGF-stimulated conditions, making healthy endothelial cells susceptible. Therefore, these perturbations may serve as effective candidate strategies for selectively targeting eNOS signaling in the high VEGF environment.

Finally, the model predicts that enhancement of PDE activity through various mechanisms can be effective in reducing cGMP. These include strategies that directly enhance the activation of PDE (increasing *k_a,PDE_* or *k_a2,PDE_*, or decreasing *k_d,PDE_* or *k_d2,PDE_*) or its catalytic activity (increasing *k_cat,PDE_*), which take effect in both the basal and VEGF-stimulated conditions (Figure 5), and strategies that sensitize PDE to cGMP (increasing *k_on,cGMP.PDE_*, decreasing *k_off,cGMP.PDE_*, or decreasing *K_M,PDE_*), which are only effective in the VEGF-stimulated condition (Figure 6). There are several existing non-specific and selective PDE inhibitors, including Theophylline and Sildenafil [110]. However, the limitation of using small molecule sGC inhibitors or PDE activators is that it is unclear whether these agents could systemically affect cGMP synthesis in other cell types, including vascular smooth muscle cells. Our model is able to differentiate between the effects of these specific mechanisms of action, and the model reveals that sensitizing PDE to cGMP may serve as a more attractive strategy for cGMP inhibition as it only affects cells in the high VEGF environment.

### 4.3. Model Limitations

We acknowledge that the results from our work are subject to some limitations. One limitation of this study is that model parameterization is difficult due to a lack of quantitative measurements of the kinetic parameter. In the absence of kinetic parameter values and additional data to calibrate the model, we have to rely on parameters from existing models. Furthermore, we did not include all the signaling events that are related to the VEGF signaling pathway. For example, DAG is also activated by PLCγ, and it activates the Ras-Raf-MEK-ERK pathway, but we do not consider this particular signaling pathway in this model in order to focus on the eNOS catalytic activity. Future work can be done to expand and integrate the pathway described in the current study with other models of the VEGF signaling pathways [46]. Another example is that not all binding partners of eNOS are taken into account. However, a recent model of competitive eNOS tuning [85] showed that NOS binding is the same under isolated or competitive conditions. This supports our model assumption that eNOS-CaM binding can be isolated in the model without adding in competitive binding of other CaM binding partners. We also acknowledge that in our model, the cGMP dynamics are loosely constrained due to the lack of quantitative longitudinal data of cGMP response to VEGF signaling. As additional data becomes available, we can incorporate cGMP and downstream ERK response dynamics into the model in order to investigate the long-term proliferative signaling in more detail.

Despite these limitations, our model provides a framework that offers mechanistic insight into the eNOS signaling pathway that is mediated by VEGF and TSP1. Future experimental studies can be used to verify the findings of this computational study. Ultimately, this work complements the models of the VEGF signaling pathway and will aid in our systematic understanding of the angiogenic regulation.

## 5. Conclusions

In summary, we have constructed and calibrated a mechanistic model that quantitatively describes the VEGF-induced eNOS signaling pathway in ECs. This model provides mechanistic insight as for how TSP1 inhibits eNOS signaling at the intracellular level. This is an aspect of TSP1′s multiple inhibitory functions that has been observed experimentally but has not been previously studied in detail. Additionally, we proposed alternative strategies that selectively inhibit the eNOS-dependent proliferative signaling in ECs experiencing a higher VEGF level that is associated with the tumor microenvironment. Therefore, this work contributes to answering a long-standing question for angiogenesis-based therapies, where systemic hypertension is often a side effect of these treatments.

## Figures and Tables

**Figure A1:**
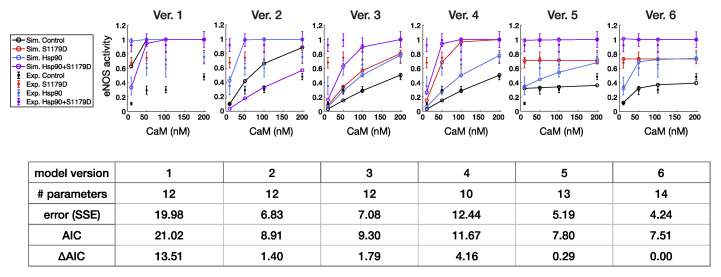
eNOS module fitting and AIC test. Each version of the eNOS model was trained using the *lsqnonlin* algorithm to match with four sets of experimental data of eNOS activity. The AIC score was computed taking into account the total number of parameters in fitting and the sum of squared error (SSE) for each model version. The difference of each model AIC score compared to the final version, ΔAIC, was computed. Hsp90, Heat Shock Protein 90; eNOS, Endothelial Nitric Oxide Synthase; CaM, Calmodulin; AIC, Akaike Information Criterion.

## Supplementary Materials

The following are available online at https://www.mdpi.com/2077-0383/9/5/1255/s1, Figure S1: Distribution of estimated parameters, Figure S2: Sensitivity analysis to inform model fitting, Figure S3: Sensitivity analyses to inform perturbation simulations, Figure S4: Model predicted dynamics with the high VEGF level (0.389 nM) (light blue), with universal perturbations on specific sets of biochemical reactions (dark grey), compared to perturbing only one reaction (light grey). (a) Decreasing Ca^2+^ binding to calmodulin (CaM) on both N- and C-terminus (decreasing both *k_on,Ca2N_* and *k_on,Ca2C_*), versus only decreasing *k_on,Ca2C_*. (b) Decreasing CaM binding to all endothelial nitric oxide synthase (eNOS) forms, including eNOS (*k_on,CaM.eNOS_*), phosphorylated eNOS (peNOS) (*k_on,CaM.peNOS_*), eNOS:Hsp90 complex (*k_on,CaM.eNOS.H_*), and peNOS:Hsp90 complex (*k_on,CaM.peNOS.H_*), versus only decreasing *k_on,CaM.eNOS_*. (c) Decreasing Arginine binding to all forms of eNOS (*k_on,eNOS.Arg_*, *k_on,peNOS.Arg_*, *k_on,eNOS.Arg.H_*, *k_on,peNOS.Arg.H_*) versus only decreasing *k_on,eNOS.Arg_*. (d) Decreasing eNOS catalytic activity (*k_cat,eNOS_*, *k_cat,peNOS_*, *k_cat,eNOS.H_*, *k_cat,peNOS.H_*) versus only decreasing *k_cat,eNOS_*; Table S1: List of model parameters, Table S2: List of perturbations; File S1: Model implementation in BioNetGen, File S2: SBML model file, File S3: List of ODEs.

## Appendix A. Supplemental Text: Model Development

### A.1. Generation of the Reaction Network Using BioNetGen

We used BioNetGen (bionetgen.org, University of Pittsburgh, Pittsburgh, PA, USA), a rule-based approach to formulate the reaction network. BioNetGen enables automated generation of the reaction network using a set of defined reaction rules [66]. This approach is particularly useful in models that involve dynamic assembly of multi-protein complexes, which occurs in our model, for example, in the case of complexes involving calcium, calmodulin, eNOS, Akt, and Hsp90. The baseline model includes 18 molecule types that can participate in 102 reaction rules, and 734 reactions were generated based on the reaction rules to form a total of 160 molecular species. The generated model is comprised of 160 non-linear ordinary differential equations (ODEs) that describes the changes in the species’ concentrations over time. The equations were implemented in MATLAB (The MathWorks, Natick, MA, USA). The model files are provided in Files S1–S3.

### A.2. Receptor Quantification

We quantified the CD47 receptor number on the surface of human microvascular endothelial cells (HMVECs) using the quantitative flow cytometry (qFLOW) technique previously described [80]. Briefly, HMVECs from Lonza (Basel, Switzerland) were cultured in flasks and maintained in Endothelial Cell Growth Media-2 (EGM-2) supplemented by the EGM-2 Single Quot Kit (Lonza). Cells were maintained at 37 °C in 95% air and 5% CO_2_, and we only use cells at passage numbers 2–4. To dissociate cells from the culture plate, cells were incubated with a non-enzymatic cell dissociation solution, CellStripper (Corning, New York, NY, USA), for 5 min at 37 °C. Cells were centrifuged at 500× *g* for 5 min to obtain a final concentration of 4 × 10^6^ cells/mL in stain buffer (PBS, bovine serum albumin, and sodium azide). Aliquots of cells (25 µL, ~10^5^ cells) were labeled with phycoerythrin (PE)-conjugated monoclonal antibodies (Biolegend, San Diego, CA, USA) and prepared for single-cell analysis via flow cytometry.

Flow cytometry was performed on a MACSQuant flow cytometer (Miltenyi, Bergisch Gladbach, Germany), and FlowJo (BD Biosciences, San Jose, CA, USA) software was used to analyze the data. To determine the number of receptors per cell, the fluorescence of Quantibrite PE beads (BD Biosciences) was measured. We followed the procedure as specified by the manufacturer and constructed a calibration curve to convert the geometric mean of PE fluorescence to the number of bound molecules. The average number of receptors on a cell in the population was then calculated. For each experiment, two biological replicates were used, and the experiments were repeated for 3–4 times. We report an average of 120,000 CD47 receptors/cell, which converts to a concentration of 199.336 nM assuming a total cell volume of 1 pL.

### A.3. Ligand-Receptor Interactions

R2 concentration is calculated based on the receptor number measured in HUVECs [80], assuming they are all active dimers. R2 dimerization has been omitted for simplicity. Therefore, we assume binding between VEGF and R2 dimers follows a 1:1 stoichiometry. The dissociation constant between VEGF and R2 is set to be *K_d_* of 0.150 nM (150 pM) based on experimental measurements [81], and assuming the association rate, *k_on,R2_* is 0.6/nM-min.

For TSP1-CD47 binding, it is assumed that TSP1 only binds to CD47 that is not coupled with R2. The association rate, *k_on,TSP1CD47_* is taken from our previous model 0.03/nM-min [56], and the dissociation rate *k_off,TSP1CD47_* is calculated to be 0.0003/min based on measured *K_d_* of 0.01 nM (10 pM) [37].

### A.4. Ligand-Ligand Binding

As in our previous tissue model [56], TSP1 binds to free VEGF, and the TSP1:VEGF complex has a degradation rate of 0.01158/min (0.000193/s). In the present model, the synthesis and degradation of the exogenous ligands (TSP1 and VEGF) are neglected, as was assumed in previous modeling works [50,52].

### A.5. Receptor Coupling

As mentioned above, TSP1 only binds to CD47 that is uncoupled with R2. Once ligated, the CD47 receptor cannot couple with R2. But, the VEGF:R2 complex can still couple with free CD47 receptor. Our handling of CD47-R2 coupling is different from Li and Finley, where no coupling is allowed for any ligated receptor. We adjusted this assumption based on the evidence [93] that TSP1 disrupts R2 and CD47 coupling, and that in the absence of TSP1, coupling between R2 and CD47 should be undisrupted upon R2 ligation.

To exclude the possibility that TSP1 only takes effect by directly forming a complex with VEGF:R2:CD47, we derived another model version where TSP1 and VEGF can both bind to their respective receptors even when CD47 and R2 are coupled. We then altered the trafficking parameters for the TSP1-containing R2 complexes. However, the model simulation showed that varying these parameters did not influence the VEGFR2 signaling dynamics, and the TSP1-containing R2 complexes are of negligible level (data not shown). Therefore, we assume that TSP1′s inhibitory function via receptor CD47 does not solely depend on its direct involvement with the ligated R2 species.

### A.6. R2 Phosphorylation

In this model, we assume R2 phosphorylation upon ligation occurs for both cell surface and internalized receptors. Since it is possible that CD47 coupling may stabilize R2 signaling in the absence of TSP1 [93], we assume the dephosphorylation rate of the CD47-coupled pR2 is slower than that of the uncoupled pR2 (0.01/min vs. 0.06/min).

### A.7. Receptor Dynamics

Internalization: free CD47 has its own internalization rate (k_inter,CD47_). TSP1:CD47 complex has its own internalization rate *k_inter,TSP1bd_*; however, due to lack of data, we set the baseline value of *k_inter,TSP1bd_* to be the same as *k_inter,CD47_* and investigated the effect of altering its value in the perturbation simulations. Receptor R2 internalization depends on its activation (phosphorylation) status and whether it has coupled with CD47. We distinguish the internalization rates of inactive R2 (*k_inter,R2_*), active R2 (*k_inter,pR2_*), inactive R2:CD47 complex (*k_inter,R2CD47_*), and active R2:CD47 complex (*k_inter,pR2CD47_*). This distinction is different from the model by Bazzazi et al. [50], where the R2 internalization rate depends on the presence of NRP1 but not CD47.

Experimental data show that there is a decrease in the receptor levels over time in the control condition (without ligand stimulation). To account for this, since only the internalized receptors are degraded, we assume that inactive R2 is also internalized. All of the aforementioned internalization rates are estimated in model fitting. All parameter estimation in this study is done using the least squares nonlinear (lsqnonlin) optimization protocol implemented in MATLAB, which minimizes the sum squared error (SSR) of the model predicted dynamics compared to the experimental data.

Degradation rate parameters: For simplicity, the unbound ligands, TSP1 and VEGF, are not subject to degradation. Instead, their disappearance occurs due to being bound to receptors that can be internalized (same as in the Bazzazi model [50,52]). Internalized (cytosolic) receptors, free CD47, the TSP1:CD47 complex, and the inactive R2:CD47 complex are assumed to have the same degradation rate *k_deg,CD47_*, which is the lowest among all the degradation rates. We estimate the value of *k_deg,CD47_* by fitting to the total R2 data in the Cycloheximide (CHX) treatment condition [67] (see below). For other R2 species, we again assign individual degradation rates based on the R2 activation status and whether it is coupled with CD47.

Rate parameters for receptor internalization and synthesis: Receptor synthesis is implemented by specifying the rate at which new receptors appear at the cell membrane. First, the values of receptor internalization (*k_inter,R2_*, *k_inter,CD47_*, *k_inter,R2:CD47_*, *k_deg,R2_* and *k_deg,CD47_*) were estimated in model fitting in order to match with CHX treatment data, where CHX blocks cellular protein synthesis) [67]. Then, the synthesis rates of R2 and CD47 were estimated to match control data (no CHX). Lastly, the degradation rates of pR2 species were estimated to fit to the datasets with VEGF stimulation (with or without CHX) while the previously estimated parameter values were fixed.

In parameter estimation, we allow the internalization rates or the degradation rates to vary across the same ranges respectively. We observed from the estimated values that *k_inter,pR2CD47_* > *k_inter,CD47_* (=*k_inter,TSP1bd_*) > *k_inter,pR2_* > *k_inter,R2_* > *k_inter,R2CD47_*, suggesting that the CD47-coupled pR2 internalizes the fastest while the CD47-coupled inactive R2 internalizes the slowest. In addition, parameter fitting showed that *k_deg,pR2_* > *k_deg,pR2CD47_*> *k_deg,R2_* > *k_deg,CD47_* (for both CD47 and CD47:R2). This trend is in line with the observation that R2 level decreases faster upon VEGF stimulation than the control [67]. The R2:CD47 degrade more slowly than their CD47-free R2 counterparts. This agrees with the hypothesis that CD47 facilitates the maintenance of active R2 signaling [93]. Importantly, we note that we did not impose that the values of the degradation and internalization parameters follow a specific order; rather, this result naturally emerged from the model optimization.

## Appendix B. Src-Hsp90-Akt Activation

VEGF activates c-Src (herein abbreviated as Src) in a T cell-specific adaptor molecule (TSAd)-dependent manner [9]. Active Src (pSrc) engages the receptor tyrosine kinase Axl, which promotes association with PI3K and activation of Akt [69]. Additionally, c-Src phosphorylates Hsp90 on Tyrosine 300 in response to R2 activation [12].

To simplify this activation process, we model that pR2 engages and activates Src, which then binds and activates Akt and Hsp90. However, as a chaperone protein, Hsp90 is assumed to bind to its partners, including Src, at a much faster rate (0.5/nM-min) than the assumed generic binding rate for most signaling proteins (0.06/nM-min). This assumption is based on the evidence that Hsp90 acts as a chaperone protein and may facilitate protein interactions by bringing multiple signaling proteins into close vicinity [111]. Additionally, large binding rates are possible [90]. Both Hsp90 and Akt are activated through phosphorylation by active Src. The kinetic rate parameters governing the activation and binding reactions of Src, Akt, and Hsp90 were specified based on existing models from literature [46,54,112] to match with experimental data.

## Appendix C. R2-Induced Ca^2+^ Influx

R2 stimulates phosphoinositol metabolism through their activation of phospholipase C-γ (PLCγ) and phosphoinositide-3 kinase (PI3K). Activation of PLCγ results in hydrolysis of phosphatidylinositol biphosphate (PIP2) to form diacylglycerol (DAG) and 1,4,5-trisphosphate (IP3), which promote protein kinase C (PKC) and Ca^2+^ signaling, respectively, to their downstream cellular targets [113]. In this study, we focus on PLCγ activation by pR2 and IP3 formation and its effect on eNOS signaling, omitting the dynamics of DAG and its downstream effect in the PKC/ERK pathway. We assign a synthesis rate of PIP2 (10/min), as PIP2 synthesis can be upregulated to compensate for its hydrolysis [114].

The IP3 can be degraded by phosphatases and kinases (the degradation rate, *k_deg,IP3_*, is assumed to be 0.06/min), and binds to its receptor IP3R on the endoplasmic reticulum (ER) membrane [114], triggering the IP3R channel to open. A slower inactivation process also pertains to this channel. As Ca^2+^ level in the ER depletes, store-operated Ca^2+^ entry (SOCE) occurs through the store-operated Ca^2+^ release-activated Ca^2+^ (CRAC) channels (reviewed in Shim 2015 and refs). Finally, Ca^2+^ is pumped back into the ER by the sarco-endoplasmic reticulum Ca^2+^ATPase (SERCA) pump. Ca^2+^can also be extruded from the cell by a number of exchange and pump, which we modeled as the plasma membrane Ca^2+^ ATPase (PMCA) pump. In addition, a passive leak of Ca^2+^ from the ER to the cytosol is included, balanced by SERCA for Ca^2+^ equilibrium in the unstimulated cells. The rate equation to calculate the intracellular and ER Ca^2+^ levels based on the fluxes through these channels and pumps are adapted from Wiesner et al. [83] and Bazzazi et al. [52].

Because it has been shown that the elevated Ca^2+^ plateau phase post VEGF-stimulation is dependent on the presence of extracellular Ca^2+^ [21], we modified the established Ca^2+^ model to include the dependence of the CRAC influx on the dynamic extracellular Ca^2+^ level. We describe the flux of Ca^2+^ from the extracellular space into the cytosol using the following Equations (A1) and (A2):

(A1) $$\frac{dJ_{CRAC}}{dt}=\frac{I_{CRAC}-J_{CRAC}}{\tau_{CRAC}}$$

(A2) $$I_{CRAC}=(Ca_{ext}*\frac{Vol_{ext}}{Vol_{cyt}}-Ca_{cyt})*\frac{I_{CRAC}K_{CRAC}^{\;n_{CRAC}}}{K_{CRAC}^{n_{CRAC}}+Ca_{ER}^{n_{CRAC}}}$$

where *J_CRAC_* is the Ca^2+^ flux across the cell membrane through the CRAC channel. *I_CRAC_* is the CRAC influx, *τ_CRAC_* is a time delay parameter. *Ca_ext_* is the extracellular Ca^2+^ concentration, *Vol_ext_* and *Vol_cyt_* represent the volumes of the extracellular space and the cytosol, respectively, *Ca_cyt_* is the cytosolic Ca^2+^ concentration, *K_CRAC_* is the Michaelis-Menten constant for CRAC influx; *n_CRAC_* is the hill coefficient, and *Ca_ER_* is the Ca^2+^ concentration in the ER.

Consequently, we estimated the values of *n_CRAC_*, *I_CRAC_*, and *K_CRAC_* after this modification. Several parameters were calculated to balance the fluxes across the cell and ER membranes for steady state Ca^2+^ equilibrium. For example, the rate of Ca^2+^ leak from the ER into the cytosol is balanced by the rate of SERCA resequestration of Ca^2+^ from cytosol back into the ER. We tuned and estimated the unknown model parameters in this module by fitting the simulated IP3 activation and intracellular Ca^2+^ dynamics upon VEGF stimulation to the datasets of HUVEC cells from Faehling et al. [21]. As the single cell datasets from this study exhibit large cell to cell heterogeneity, as is the case with other papers presenting EC Ca^2+^ influx data [115,116], we estimated the averaged levels of Ca^2+^ and IP3 from the datasets, representing an average cell, and used these values in model training. We assume the basal level of intracellular Ca^2+^ to be 50 nM [21,116]. For IP3 levels, we used data from Faehling et. al. Figure 4 [21], taking the average value at each timepoint for all three sets of single cell data.

## Appendix D. eNOS Module

### D.1. Ca-CaM-eNOS Interactions

Free intracellular Ca^2+^ binds to and activates CaM. We adapt a four-state Ca^2+^-CaM binding model from previous models [85,117], in which it is assumed that binding of two Ca^2+^ at each CaM terminus can be treated as a single event due to the highly cooperative binding of Ca^2+^ at each terminus. The Ca^2+^-saturated or partially saturated CaM binds and activates eNOS, and no eNOS-CaM binding was observed in Ca^2+^-free solution (EDTA) [118]. Based on values from both the experimental study and computational models, we set the forward binding rate of eNOS with the C-terminus saturated CaM and fully saturated CaM to be 0.078/nM-min, the binding rate of eNOS with the N-terminus saturated CaM to be 0.0081/nM-min, and the dissociation rate of eNOS from all forms of CaM to be 0.6/min (0.01/s). Additionally, a 2017 study by Chen et al. revealed that different conformational edits to the eNOS can enhance its affinity with CaM [82].

Not all binding partners of eNOS are taken into account. However, a recent model of competitive eNOS tuning [85] showed that NOS binding is the same under isolated or competitive conditions. This supports our model assumption that eNOS-CaM binding can be isolated in the model without adding in competitive binding of other CaM binding partners.

### D.2. eNOS Catalytic Activity

Compelling evidence shows that both Akt and Hsp90 regulate eNOS catalytic activity. A study by McGabe et al. suggests that eNOS phosphorylation at the S1179 site by Akt decreases the dissociation of CaM from eNOS and increases the eNOS catalytic activity [119]. Another study showed that as a scaffolding protein, Hsp90 facilitates CaM binding to eNOS, and a synergistic effect on eNOS activation where Hsp90 enhances the extent of eNOS phosphorylation by Akt [120]. Furthermore, Chen et al. demonstrated that eNOS predominantly exists in the dimeric form and is stabilized by Hsp90, whereas the eNOS monomer is less stable and is subject to ubiquitination [121]. The dimeric eNOS form is responsive to regulation (such as phosphorylation) on its activity. In addition, destabilization of eNOS dimers results in eNOS degradation by proteasome, accompanied by its dephosphorylation. For simplicity, we assume that all eNOS in this model is in the form of a dimer. To account for the function of Hsp90 on maintaining the eNOS level, we assign a non-zero degradation rate for peNOS (phosphorylated eNOS dimer) when it is unassociated with Hsp90.

eNOS binds to its substrate arginine in a reversible manner. Kinetic studies of eNOS binding with its substrates [122] reported the binding rate of eNOS and arginine to be 0.012–0.048/nM-min, and dissociation rate 4.8–96/min (as summarized in an article by Chen and Popel [123]). Meanwhile, the eNOS catalytic rate varies largely across experimental and modeling studies [122,123,124]. Therefore, we fit the catalytic rates by fitting to experimental data.

Given the evidence that Ca-CaM, Akt, and Hsp90 can influence the affinity of eNOS with its substrate and eNOS catalytic activity differently, we used an iterative approach in constructing and training this model. The experimental data that we used for training this minimal model include a dataset from Takahashi and Mendelsohn [120] showing Hsp90 influencing eNOS phosphorylation by Akt, and a dataset from Chen et al. showing eNOS catalytic activity dependence on CaM, Hsp90, and its phosphorylation status [82]. We started with a relatively simple system, where the various forms of the eNOS species share the same affinity with the substrate arginine, only distinguishing the catalytic rates for basal eNOS, phosphorylated eNOS, and Hsp90-bound eNOS. As fitting results reveal inadequacy of the module structure, we modified the module several times to incorporate differences in the eNOS catalytic activity levels for the various species (Figure A1).

Briefly, we first tested Michaelis-Menten kinetics for the biochemical reactions in module version 1, and then tested mass-action reaction kinetics in module version 2. Based on the model fitting and analysis, we found that the various forms of eNOS must have both different affinities and catalytic rates in order for the predicted eNOS activities to match the experimental data. We further evaluated the adequacy of the model structure by seeing which structure could achieve the observed dynamics from a separate dataset. We determined that Hsp90′s effect on eNOS catalytic activity includes not only a faster reaction for Akt-mediated phosphorylation of eNOS via increased affinity between eNOS and CaM (version 3), but also another layer of enhanced activity. Therefore, we further altered the model structure by assigning separate catalytic rates for the Hsp90-bound eNOS forms (version 4). In version 5, in order to match with the datapoints with the lowest CaM level, we let peNOS be active independently from CaM. When we discovered that only assigning a different catalytic rate is not sufficient to separate the two initial data points (control vs. Hsp90-present) in the Chen dataset, we further assigned different affinities to the Hsp90-bound eNOS with L-Arginine to allow the model to fit to the two different levels of eNOS catalytic activity used experimentally (version 6).

Since we trained the eNOS module using the experimental datasets from purified protein mixture studies [82,120], we assumed that phosphatases, ubiquitin, and proteasomes do not exist in the mixture. Therefore, the dephosphorylation and degradation rates were set as zero. We then estimated their values during full model training using in vitro cell culture datasets.

### D.3. AIC Test

We compared the quality of the various eNOS module versions using the Akaike Information Criterion (AIC), which measures the quality of a model to represent a given set of data while accounting for the number of parameters estimated [125] (Figure A1). Specifically, we calculate the AIC score for each best fitted eNOS module version using the following Equation (A3), as used in a previous study [55].

(A3) $$AIC=n\times log\left(\frac{SSE}{n}\right)+2k$$

where *n* is the number of data points, *SSE* is the sum of the squared error between the data and the model predictions, and *k* is the number of parameters used to fit the model. The module version with the smallest AIC is assumed to be the best version. We also calculate and report the change in the AIC value for each module version, relative to the version with the smallest AIC. This provides insight into how changing the complexity of the module and the number of fitted parameters influences the fit.

## Appendix E. sGC-PDE Activity

We adapted the model by Halvey et al. for downstream sGC activity [86]. This model describes the kinetic of NO-cGMP signaling in rat platelets and was adapted for rat cerebella cells as well. sGC is activated by NO binding distally to the heme prosthetic group, and then the proximal coordinating histidine bond breaks, resulting in an sGC conformational change. A second NO can bind to the vacant proximal heme site with a very low dissociation rate, desensitizing sGC to the NO level (*k_on,NO.NOGC_*). When the distal NO dissociates from sGC (*k_f,NOGC.NO_*), this results in a NO-bound inactive state of sGC. Active sGC generates cGMP from GTP, which binds to PDE on the agonist site and PDE hydrolyzes cGMP at its catalytic site. All forms of PDE, the free form and two cGMP-bound states are assumed to hydrolyze cGMP, as depicted in the Halvey model [86].

In this part of the signaling pathway, we must also account for NO clearance. The half-life of NO is 30 s in physiological buffers (corresponding to a decay constant of 1.38/min) [126]. However, NO has a much shorter half-life (~4 s) under physiological conditions (corresponding to a decay constant of 10.4/min) [127]. In a more recent study, the clearance rate of NO under flow condition was estimated to be 20–30/min [87]. Therefore, we allow the NO clearance rate parameter *k_clearNO_* to vary within the range of 5–15 min in training the full model.

To match model simulations with the endothelial cell data, we estimated two unknown model parameters, the catalytic rate of activated sGC, *k_cat,sGC_*, and the catalytic rate of activated PDE5, *k_cat,PDE5a_*, by fitting to experimental data of cGMP level at 10 min in HUVECs stimulated by 30 ng/mL VEGF [72]. Our model-simulated cGMP synthesis exhibits a transient increase, similar to that observed in a previous study using VEGF-stimulated bovine aortic endothelial cells [16].
