# Supplementary material for: Mathematical Model Predicts Effective Strategies to Inhibit VEGF-eNOS Signaling
Source: J Clin Med. 2020 Apr 26;9(5):1255. doi: 10.3390/jcm9051255 (PMC7287924; doi:10.3390/jcm9051255)
Supplement: Supplementary file 1 [file jcm-09-01255-s001.zip › jcm-727669-US-SI-supplementary-for conversion 2/File S1 Model implementation in BioNetGen.pdf]

```
begin model
#all units in nM/min
begin molecule types

Trash()
null()
R2(VEGF,CD47,Y1175~U~P,loc~mem~cyt,Src)
CD47(TSP1,R2,loc~mem~cyt)
TSP1(CD47,VEGF)
VEGF(R2,TSP1)
Src(R2,Hsp90,Y~U~P,Akt)
Hsp90(Src,Akt,Y300~U~P,eNOS)
Akt(Hsp90,eNOS,T~U~P,Src)
eNOS(CaM,Hsp90,S1177~U~P,Arg,Akt)
Arg(eNOS)
NO()
Cit()
PI(PIsite~3P~4P)
PLCg(Yplc~Y~P)
Istim()
Ca(CaM)
CaM(C,N,eNOS)
IP3()
Castore()
NObuffer()
sGC_b()
sGC_6c()
sGC_5c()
sGC_5c_NO()
sGC_NO()
sGC()
GTP()
cGMP()
PDE()
cGMP_PDE()
cGMP_PDE_a()
GMP()

end molecule types

################################################################################################
################################################################################################
################################################################################################

begin observables

Molecules       Trash_tot       Trash
Molecules       null_tot        null
Molecules       totR2           R2
Molecules       totR2_mem       R2(loc~mem)
Molecules       totR2_cyt       R2(loc~cyt)
Molecules       totpR2          R2(Y1175~P)
Molecules       tot_R2_bound    R2(VEGF!1).VEGF(R2!1)
Molecules       CD47TSP1        CD47(R2,TSP1!2).TSP1(CD47!2)
Molecules       totCD47         CD47()
Molecules       totSrc          Src()
Molecules       pSrc            Src(Y~P)
Molecules       totAkt          Akt()
```

```
Molecules          pAkt            Akt(T~P)
Molecules          toteNOS         eNOS()
Molecules          peNOS           eNOS(S1177~P)
Molecules          yPLCg           PLCg(Yplc~Y)
Molecules          Iopenstim       Istim()
Molecules          freepip2        PI(PIsite~3P)
Molecules          tot_Cac         Ca()
Molecules          freeCac         Ca(CaM)
Molecules          IP3_ob          IP3()
Molecules          CaER            Castore
Molecules          Cit_prod        Cit()
Molecules          NO_ob           NO()
Molecules          active_sGC      sGC_5c()
Molecules          free_GTP        GTP()
Molecules          cGMP_ob         cGMP()
Molecules          PDE_ob          PDE()
Molecules          cGMP_PDE_ob     cGMP_PDE()
Molecules          cGMP_PDE_a_ob   cGMP_PDE_a()
Molecules          totPLCg         PLCg()
Molecules          pPLCg           PLCg(Yplc~P)

end observables
###############################################################################################################
###############################################################################################################
###############################################################################################################

begin parameters
################################ TSP1-VEGF  ################################
kon_TSP1_VEGF        0.03      #/nM-min
koff_TSP1_VEGF       0.3       #/min
kdeg_VEGF            0.0116    #/min TSP1:VEGF degradation
konR2                0.6       # /nM-min
koffR2               0.09      # /min
konTSP1CD47          0.03      #
koffTSP1CD47         0.0003
konR2CD47            0.0011
koffR2CD47           0.06
kinterR2             0.0426
kinterpR2            4.7682
kinterCD47           0.5692
kinterR2_CD47        0.0085
kinterpR2_CD47       23.8410
kinterTSP1bd         0.5692
kpR2                 0.6
kdpR2                0.06
kdpR2CD47bd          0.01
ksynR2               0.0482
ksynCD47             0.0899
kdegR2               0.0126
kdegpR2              0.2219
kdegpR2_CD47         0.0168
kdegCD47             0.0063
kdegR2TSP1bd         0.0168
konSrc_pR2           0.06
koffSrc_pR2          25
kp_Src               20
kdp_Src              0.4
konSrc_Hsp90         0.5
```

koffSrc_Hsp90	0.5	
kp_Hsp90	30	
kdp_Hsp90	0.01	
konSrc_Akt	0.06	
koffSrc_Akt	30	
kp_Akt	3	
kdp_Akt	0.1	
kpPLCg	10	
kdpPLCg	0.3	
kmPIP2PLCg	193.585826	
nIP3	1	
kcatPLCgDAG	0.3	
kdeg_ip3	0.02	
kPIP2gen	10	
ICrac	1.5e-9	
Kcrac	2492.5441	
tau_stim	1	
kmPLCgR2	8e3	
ncrac	0.6580	
Iip3Ramp	0.3910	
KmIP3R	20000	
I_PMCA	220418.474925993	
KmPMCA	260	
Caext	2000e3	
I_SERCA	435320.590000000	
KleakER	6.8022e-9	
KmSERCA	0.15e3	
KiCa	1e3	
CSQN_total	15000e3	
KCSQN	800e3	
Volcyto	9.12E-13	
Volext	0.0005	
VolER	3.35E-13	
konCa2C	0.240	#/(nM*min)
koffCa2C	555	#/min
konCa2N	6	#/(nM*min)
koffCa2N	4.5e4	#/min
kon2NeNOS	0.0081	
kon2CeNOS	0.078	
kon4eNOS	0.078	
koffCaMeNOS	0.6	
kon2NeNOS_H	0.0081/0.249	
kon2CeNOS_H	0.078/0.249	
kon4eNOS_H	0.078/0.249	
koffCaMeNOS_H	0.6	
kon2NpeNOS	0.0081/0.12	
kon2CpeNOS	0.078/0.12	
kon4peNOS	0.078/0.12	
koffCaMpeNOS	0.6	
kon2NpeNOS_H	0.0081/0.07	
kon2CpeNOS_H	0.078/0.07	
kon4peNOS_H	0.078/0.07	
koffCaMpeNOS_H	0.6	
konAkt_eNOS	0.02	
koffAkt_eNOS	4.038	
konpAkt_pHsp90	0.5	
koffpAkt_pHsp90	10.2826	

konHsp90_eNOS	0.5
koffHsp90_eNOS	5.7327
konHsp90_CaMeNOS	0.5
koffHsp90_CaMeNOS	10.0976
kcat_pAkt	3
kcat_pAkt_H	10
kdp_eNOS	3.5
kdeg_eNOS	0.02
kon_beNOS_Arg	0.048
koff_beNOS_Arg	96
kon_eNOS_Arg	0.048
koff_eNOS_Arg	2.9023
kon_peNOS_Arg	0.048
koff_peNOS_Arg	4.9891
kon_eNOS_Arg_H	0.048
koff_eNOS_Arg_H	6.987
kon_peNOS_Arg_H	0.048
koff_peNOS_Arg_H	10.8397
kcat_eNOS	1.49
kcat_peNOS	2.6820
kcat_eNOS_H	3.132
kcat_peNOS_H	4.22
kclearNO	10
kon1	18
koff1	360
kon2	1680
koff2	1680
kon3	0.24
koff3	60000
kon4	120000
koff4	0.108
kon5	24
koff5	0.044
kon6	60
koff6	0.06
kcat_sGC	31.5
kp1	0.001
kdp1	7.752
kp2	18
kdp2	7.2
Kp1	4e3
Vp2	0.2925
Kp2	1e3
TSP1_0	0
VEGF_0	0
R2_0	8.971
CD47_0	199.336
Src_0	344
Ca_0	50
Ca_store_0	2e6
CaM_0	30
eNOS_0	100
Arg_0	100000
Hsp90_0	500
Akt_0	800
sGC_b_0	10
GTP_0	500000

```
PIP2_0                          200000
PLCg_0                          500
Istim0                          19990.3745
PDE_0                           5e5

######################
RprodGMP            Vp2*0.05*(PDE_ob+cGMP_PDE_ob)/(Kp1+cGMP_ob) + Vp2*(cGMP_PDE_a_ob)/(Kp2+cGMP_ob)  #
###################
RF1                 kpPLCg/(kmPLCgR2+yPLCg)
RF2         kcatPLCgDAG*freepip2^(nIP3)/(kmPIP2PLCg^nIP3+freepip2^nIP3)
RF3                 ((Volext/Volcyto)*Caext-freeCac)*ICrac*(Kcrac^ncrac/(Kcrac^ncrac+CaER^ncrac))/tau_stim-
Iopenstim/tau_stim # #added (Caext-freeCac)
RF4                 (VolER/Volcyto)*Iip3Ramp*(CaER-
freeCac)*((IP3_ob)^3.8/((IP3_ob)^3.8+KmIP3R^3.8))*(KiCa^3.8/(KiCa^3.8+freeCac^3.8))
RF5         -I_PMCA*freeCac^1.4/(KmPMCA^1.4+freeCac^1.4) + Iopenstim
RF6                 -I_SERCA*(freeCac/(KmSERCA+freeCac))^2 + KleakER*(CaER-freeCac)^2 # SERCA pump
RF7                 -Iip3Ramp*(CaER-freeCac)*(IP3_ob^3.8/(IP3_ob^3.8+KmIP3R^3.8))*(KiCa^3.8/(KiCa^3.8+freeCac^3.8))*
( 1/(1+CSQN_total/(KCSQN+CaER)^2) )  # CICR. CSQN: calsequestration (ER calcium buffer)
RF8                 I_SERCA*(freeCac/(KmSERCA+freeCac))^2*(Volcyto/VolER)*(1/(1+CSQN_total/(KCSQN+CaER)^2)) # SERCA
pump
RF9                 -KleakER*(Volcyto/VolER)*((CaER-freeCac)^2)*(1/(1+CSQN_total/(KCSQN+CaER)^2))

end parameters
##############################################################################################
##############################################################################################
##############################################################################################

begin seed species

#dummy variable
Trash()                                         0
null()                                          1

TSP1(CD47,VEGF)                                 TSP1_0
VEGF(R2,TSP1)                                   VEGF_0
R2(VEGF,CD47,Y1175~U,loc~mem,Src)               R2_0
CD47(TSP1,R2,loc~mem)                           CD47_0
Src(R2,Hsp90,Y~U,Akt)                           Src_0
Hsp90(Src,Akt,Y300~U,eNOS)                      Hsp90_0
Akt(Hsp90,T~U,eNOS,Src)                         Akt_0
eNOS(CaM,Hsp90,S1177~U,Arg,Akt)                 eNOS_0
Ca(CaM)                                         Ca_0
CaM(C,N,eNOS)                                   CaM_0
Arg(eNOS)                                       Arg_0
sGC_b()                                         sGC_b_0
GTP()                                           GTP_0
NObuffer()                                      0
PI(PIsite~3P)                                   PIP2_0
PLCg(Yplc~Y)                                    PLCg_0
Istim()                                         Istim0
Castore()                                       Ca_store_0
PDE()                                           PDE_0

end seed species

##############################################################################################
##############################################################################################
##############################################################################################
```

```
begin reaction rules
################### START receptor MODULE   #################

### TSP1 VEGF binding, VEGF can bind to TSP1
TSP1(CD47,VEGF) + VEGF(R2,TSP1) <->  TSP1(CD47,VEGF!1).VEGF(TSP1!1,R2) kon_TSP1_VEGF, koff_TSP1_VEGF
TSP1(CD47,VEGF!1).VEGF(TSP1!1,R2) -> Trash() kdeg_VEGF #from Li and Finley

### receptor-ligand binding
VEGF(R2,TSP1) + R2(VEGF,Y1175~U,loc~mem,Src,CD47) <-> VEGF(R2!1,TSP1).R2(VEGF!1,Y1175~U,loc~mem,Src,CD47)
        konR2,koffR2
VEGF(R2,TSP1) + R2(VEGF,Y1175~U,loc~mem,Src,CD47!1).CD47(TSP1,R2!1) <->
VEGF(R2!2,TSP1).R2(VEGF!2,Y1175~U,loc~mem,Src,CD47!1).CD47(TSP1,R2!1)          konR2,koffR2

# TSP1 only binds to CD47 not bound to R2
TSP1(CD47,VEGF) + CD47(TSP1,loc~mem,R2) <-> TSP1(CD47!1,VEGF).CD47(TSP1!1,loc~mem,R2)
        konTSP1CD47,koffTSP1CD47

### receptor coupling
# R2 only couples with CD47 without TSP1
R2(CD47,loc~mem) + CD47(R2,loc~mem,TSP1) <-> R2(CD47!2,loc~mem).CD47(R2!2,loc~mem,TSP1)
        konR2CD47,koffR2CD47

### receptor internalization - depends on R2 phosphorylation and TSP1 binding only.
R2(CD47,Y1175~U,loc~mem,Src) -> R2(CD47,Y1175~U,loc~cyt,Src)    kinterR2
R2(CD47,Y1175~P,loc~mem,Src) -> R2(CD47,Y1175~P,loc~cyt,Src)    kinterpR2
R2(CD47!1,Y1175~U,loc~mem,Src).CD47(TSP1,R2!1,loc~mem) -> R2(CD47!1,Y1175~U,loc~cyt,Src).CD47(TSP1,R2!1,loc~cyt)
        kinterR2_CD47
R2(CD47!1,Y1175~P,loc~mem,Src).CD47(TSP1,R2!1,loc~mem) -> R2(CD47!1,Y1175~P,loc~cyt,Src).CD47(TSP1,R2!1,loc~cyt)
        kinterpR2_CD47

CD47(TSP1,R2,loc~mem) -> CD47(TSP1,R2,loc~cyt)        kinterCD47
CD47(TSP1!1,R2,loc~mem).TSP1(CD47!1,VEGF) -> CD47(TSP1!1,R2,loc~cyt).TSP1(CD47!1,VEGF)     kinterTSP1bd

### R2 phosphorylation and dephosphorylation
VEGF(R2!1,TSP1).R2(VEGF!1,CD47,Y1175~U,Src) <-> VEGF(R2!1,TSP1).R2(VEGF!1,CD47,Y1175~P,Src)        kpR2,kdpR2
VEGF(R2!1,TSP1).R2(VEGF!1,CD47!2,Y1175~U,Src).CD47(TSP1,R2!2) <->
VEGF(R2!1,TSP1).R2(VEGF!1,CD47!2,Y1175~P,Src).CD47(TSP1,R2!2)  kpR2,kdpR2CD47bd

### secretion/degradation - degradation depends on whether VEGF, CD47, or TSP1 is in complex - assuming phosphorylation
affects degradation (Nguyen 2013)
null -> null + R2(VEGF,CD47,Y1175~U,loc~mem,Src)        ksynR2
null -> null + CD47(TSP1,R2,loc~mem)                    ksynCD47

R2(VEGF,CD47,loc~cyt,Src,Y1175~U) -> Trash()                        kdegR2
VEGF(R2!1,TSP1).R2(VEGF!1,CD47,loc~cyt,Src) -> Trash()              kdegR2 #VEGF:R2 or VEGF:pR2
R2(VEGF,CD47,loc~cyt,Src,Y1175~P) -> Trash()                        kdegpR2 #VEGF:R2 or VEGF:pR2
VEGF(R2!1,TSP1).R2(VEGF!1,CD47,loc~cyt,Src,Y1175~P) -> Trash()      kdegpR2 #VEGF:R2 or VEGF:pR2
R2(CD47!2,loc~cyt,Src,Y1175~P).CD47(TSP1,R2!2,loc~cyt) -> Trash()   kdegpR2_CD47 #VEGF:R2:CD47 or VEGF:pR2:CD47
CD47(R2,loc~cyt) -> Trash()                                                 kdegCD47
R2(CD47!2,loc~cyt,Src,Y1175~U).CD47(TSP1,R2!2,loc~cyt) -> Trash()           kdegCD47

###############  END receptor MODULE #################
################   START UPSTREAM MODULE   ##############
##internalized pR2 binds to uSrc
R2(Y1175~P,Src,loc~cyt) + Src(R2,Y~U) <-> R2(Y1175~P,Src!1,loc~cyt).Src(R2!1,Y~U)       konSrc_pR2,koffSrc_pR2
Src(R2!1,Y~U).R2(Y1175~P,Src!1,loc~cyt) -> Src(R2,Y~P) + R2(Y1175~P,Src,loc~cyt)        kp_Src
Src(Y~P) -> Src(Y~U) kdp_Src
```

```
#Hsp90 can associate with Src (whether Src is associated with R2 and phosphorylated)
Src(R2,Hsp90,Y~?) + Hsp90(Src,Akt,Y300~U,eNOS) <-> Src(R2,Hsp90!1,Y~?).Hsp90(Src!1,Akt,Y300~U,eNOS)
        konSrc_Hsp90,koffSrc_Hsp90
Src(R2,Hsp90!1,Y~P).Hsp90(Src!1,Akt,Y300~U,eNOS) -> Src(R2,Hsp90,Y~P) + Hsp90(Src,Akt,Y300~P,eNOS)    kp_Hsp90
Hsp90(Y300~P,eNOS) -> Hsp90(Y300~U,eNOS)    kdp_Hsp90

#free Akt is phosphorylated by Src
Src(R2,Hsp90,Y~?,Akt) + Akt(Hsp90,T~U,Src,eNOS) <-> Src(R2,Hsp90,Y~?,Akt!1).Akt(Hsp90,T~U,Src!1,eNOS)
        konSrc_Akt,koffSrc_Akt
Src(R2,Hsp90,Y~P,Akt!1).Akt(Hsp90,T~U,Src!1,eNOS) -> Src(R2,Hsp90,Y~P,Akt) + Akt(Hsp90,T~P,Src,eNOS)  kp_Akt
Akt(T~P,eNOS) -> Akt(T~U,eNOS)        kdp_Akt

#################  END upstream module addition    ############################
####################  START Ca module ######################################

PLCg(Yplc~Y) + R2(Y1175~P,loc~mem) -> PLCg(Yplc~P) + R2(Y1175~P,loc~mem)            RF1
PLCg(Yplc~Y) + R2(Y1175~P,loc~cyt) -> PLCg(Yplc~P) + R2(Y1175~P,loc~cyt)     RF1
PLCg(Yplc~P) -> PLCg(Yplc~Y)                                              kdpPLCg
PLCg(Yplc~P) + PI(PIsite~3P) -> IP3() + PLCg(Yplc~P)                          RF2
null-> null+ PI(PIsite~3P)                                                kPIP2gen
IP3() -> Trash()                                                          kdeg_ip3
null-> null+ Istim()         RF3
null-> null+ Ca(CaM)         RF4
null-> null+ Ca(CaM)         RF5
null-> null+ Ca(CaM)         RF6
null-> null+ Castore()       RF7
null-> null+ Castore()       RF8
null-> null+ Castore()       RF9


#################### END Ca module #########################################
#################### Start Hsp90 module ######################################
## M4 edit: allow CaM to bind to Arg-bound eNOS and Akt-bound eNOS
#### Ca binding to CaM, CaM activating eNOS
Ca(CaM) + Ca(CaM) + CaM(C,N,eNOS) <-> Ca(CaM!1).CaM(C!1,N,eNOS)        konCa2C,koffCa2C
Ca(CaM) + Ca(CaM) + CaM(C,N,eNOS) <-> Ca(CaM!1).CaM(C,N!1,eNOS)        konCa2N,koffCa2N

Ca(CaM) + Ca(CaM) + Ca(CaM!1).CaM(C,N!1,eNOS!?) <-> Ca(CaM!2).Ca(CaM!1).CaM(C!2,N!1,eNOS!?)        konCa2C,koffCa2C
Ca(CaM) + Ca(CaM) + Ca(CaM!1).CaM(C!1,N,eNOS!?) <-> Ca(CaM!2).Ca(CaM!1).CaM(C!1,N!2,eNOS!?)        konCa2N,koffCa2N

##this assumes only Ca-bound CaM can bind to eNOS
Ca(CaM!1).CaM(C!1,N,eNOS) + eNOS(CaM,Hsp90,S1177~U,Akt) <->
Ca(CaM!1).CaM(C!1,N,eNOS!2).eNOS(CaM!2,Hsp90,S1177~U,Akt)    kon2CeNOS,koffCaMeNOS
Ca(CaM!1).CaM(C,N!1,eNOS) + eNOS(CaM,Hsp90,S1177~U,Akt) <->
Ca(CaM!1).CaM(C,N!1,eNOS!2).eNOS(CaM!2,Hsp90,S1177~U,Akt)    kon2NeNOS,koffCaMeNOS
Ca(CaM!1).Ca(CaM!2).CaM(C!1,N!2,eNOS) + eNOS(CaM,Hsp90,S1177~U,Akt) <->
Ca(CaM!1).Ca(CaM!2).CaM(C!1,N!2,eNOS!3).eNOS(CaM!3,Hsp90,S1177~U,Akt)           kon4eNOS,koffCaMeNOS

Ca(CaM!1).CaM(C!1,N,eNOS) + eNOS(CaM,Hsp90!3,S1177~U,Akt).Hsp90(eNOS!3) <->
Ca(CaM!1).CaM(C!1,N,eNOS!2).eNOS(CaM!2,Hsp90!3,S1177~U,Akt).Hsp90(eNOS!3)      kon2CeNOS_H,koffCaMeNOS_H
Ca(CaM!1).CaM(C,N!1,eNOS) + eNOS(CaM,Hsp90!3,S1177~U,Akt).Hsp90(eNOS!3) <->
Ca(CaM!1).CaM(C,N!1,eNOS!2).eNOS(CaM!2,Hsp90!3,S1177~U,Akt).Hsp90(eNOS!3)      kon2NeNOS_H,koffCaMeNOS_H
Ca(CaM!1).Ca(CaM!2).CaM(C!1,N!2,eNOS) + eNOS(CaM,Hsp90!3,S1177~U,Akt).Hsp90(eNOS!3) <->
Ca(CaM!1).Ca(CaM!2).CaM(C!1,N!2,eNOS!3).eNOS(CaM!3,Hsp90!4,S1177~U,Akt).Hsp90(eNOS!4)
        kon4eNOS_H,koffCaMeNOS_H
```

Ca(CaM!1).CaM(C!1,N,eNOS) + eNOS(CaM,Hsp90,S1177~P,Akt) <->
Ca(CaM!1).CaM(C!1,N,eNOS!2).eNOS(CaM!2,Hsp90,S1177~P,Akt) kon2CpeNOS,koffCaMpeNOS
Ca(CaM!1).CaM(C,N!1,eNOS) + eNOS(CaM,Hsp90,S1177~P,Akt) <->
Ca(CaM!1).CaM(C,N!1,eNOS!2).eNOS(CaM!2,Hsp90,S1177~P,Akt) kon2NpeNOS,koffCaMpeNOS
Ca(CaM!1).Ca(CaM!2).CaM(C!1,N!2,eNOS) + eNOS(CaM,Hsp90,S1177~P,Akt) <->
Ca(CaM!1).Ca(CaM!2).CaM(C!1,N!2,eNOS!3).eNOS(CaM!3,Hsp90,S1177~P,Akt) kon4peNOS,koffCaMpeNOS

Ca(CaM!1).CaM(C!1,N,eNOS) + eNOS(CaM,Hsp90!3,S1177~P,Akt).Hsp90(eNOS!3) <->
Ca(CaM!1).CaM(C!1,N,eNOS!2).eNOS(CaM!2,Hsp90!3,S1177~P,Akt).Hsp90(eNOS!3) kon2CpeNOS_H,koffCaMpeNOS_H
Ca(CaM!1).CaM(C,N!1,eNOS) + eNOS(CaM,Hsp90!3,S1177~P,Akt).Hsp90(eNOS!3) <->
Ca(CaM!1).CaM(C,N!1,eNOS!2).eNOS(CaM!2,Hsp90!3,S1177~P,Akt).Hsp90(eNOS!3) kon2NpeNOS_H,koffCaMpeNOS_H
Ca(CaM!1).Ca(CaM!2).CaM(C!1,N!2,eNOS) + eNOS(CaM,Hsp90!3,S1177~P,Akt).Hsp90(eNOS!3) <->
Ca(CaM!1).Ca(CaM!2).CaM(C!1,N!2,eNOS!3).eNOS(CaM!3,Hsp90!4,S1177~P,Akt).Hsp90(eNOS!4)
kon4peNOS_H,koffCaMpeNOS_H

Ca(CaM!1).CaM(C!1,N,eNOS) + eNOS(CaM,Akt!1,Hsp90,S1177~U).Akt(eNOS!1,Hsp90) <->
Ca(CaM!1).CaM(C!1,N,eNOS!2).eNOS(CaM!2,Akt!3,Hsp90,S1177~U).Akt(eNOS!3,Hsp90) kon2CpeNOS,koffCaMpeNOS
Ca(CaM!1).CaM(C,N!1,eNOS) + eNOS(CaM,Akt!1,Hsp90,S1177~U).Akt(eNOS!1,Hsp90) <->
Ca(CaM!1).CaM(C,N!1,eNOS!2).eNOS(CaM!2,Akt!3,Hsp90,S1177~U).Akt(eNOS!3,Hsp90) kon2NpeNOS,koffCaMpeNOS
Ca(CaM!1).Ca(CaM!2).CaM(C!1,N!2,eNOS) + eNOS(CaM,Akt!1,Hsp90,S1177~U).Akt(eNOS!1,Hsp90) <->
Ca(CaM!1).Ca(CaM!2).CaM(C!1,N!2,eNOS!3).eNOS(CaM!3,Akt!4,Hsp90,S1177~U).Akt(eNOS!4,Hsp90)
kon4peNOS,koffCaMpeNOS

Ca(CaM!1).CaM(C!1,N,eNOS) + eNOS(CaM,Akt,Hsp90!1,S1177~U).Hsp90(eNOS!1,Akt!2,Y300~P).Akt(Hsp90!2,eNOS) <->
Ca(CaM!1).CaM(C!1,N,eNOS!2).eNOS(CaM!2,Akt,Hsp90!3,S1177~U).Hsp90(eNOS!3,Akt!4,Y300~P).Akt(Hsp90!4,eNOS)
kon2CpeNOS_H,koffCaMpeNOS_H
Ca(CaM!1).CaM(C,N!1,eNOS) + eNOS(CaM,Akt,Hsp90!1,S1177~U).Hsp90(eNOS!1,Akt!2,Y300~P).Akt(Hsp90!2,eNOS) <->
Ca(CaM!1).CaM(C,N!1,eNOS!2).eNOS(CaM!2,Akt,Hsp90!3,S1177~U).Hsp90(eNOS!3,Akt!4,Y300~P).Akt(Hsp90!4,eNOS)
kon2NpeNOS_H,koffCaMpeNOS_H
Ca(CaM!1).Ca(CaM!2).CaM(C!1,N!2,eNOS) +
eNOS(CaM,Akt,Hsp90!1,S1177~U).Hsp90(eNOS!1,Akt!2,Y300~P).Akt(Hsp90!2,eNOS) <->
Ca(CaM!1).Ca(CaM!2).CaM(C!1,N!2,eNOS!3).eNOS(CaM!3,Akt,Hsp90!4,S1177~U).Hsp90(eNOS!4,Akt!5,Y300~P).Akt(Hsp90!5,eN
OS) kon4peNOS_H,koffCaMpeNOS_H

both Akt and eNOS preferentially binds to Hsp90. Hsp90 binding docks Akt and eNOS; increases CaM affinity to eNOS
#if peNOS will not bind to Akt again
eNOS(CaM,Akt,Hsp90) + Hsp90(Akt,eNOS,Y300~P) <-> eNOS(CaM,Akt,Hsp90!1).Hsp90(Akt,eNOS!1,Y300~P)
konHsp90_eNOS,koffHsp90_eNOS
CaM(eNOS!1).eNOS(CaM!1,Akt,Hsp90) + Hsp90(Akt,eNOS,Y300~P) <->
CaM(eNOS!1).eNOS(CaM!1,Akt,Hsp90!2).Hsp90(Akt,eNOS!2,Y300~P)
konHsp90_CaMeNOS,koffHsp90_CaMeNOS
eNOS(Akt,Hsp90,S1177~U) + Akt(Hsp90,eNOS,T~P)<-> eNOS(Akt!1,Hsp90,S1177~U).Akt(eNOS!1,Hsp90,T~P)
konAkt_eNOS,koffAkt_eNOS
Akt(Hsp90,eNOS,T~P) + Hsp90(Akt,eNOS,Y300~P) <-> Akt(Hsp90!1,eNOS,T~P).Hsp90(Akt!1,eNOS,Y300~P)
konpAkt_pHsp90,koffpAkt_pHsp90

###assume Akt:Hsp90 complex is active (Akt and Hsp90 can be unphosphorylated)
eNOS(CaM,Akt,Hsp90,S1177~U) + Akt(Hsp90!1,eNOS).Hsp90(Akt!1,eNOS) <->
eNOS(CaM,Akt,Hsp90!1,S1177~U).Hsp90(eNOS!1,Akt!2).Akt(Hsp90!2,eNOS) konHsp90_eNOS,koffHsp90_eNOS
eNOS(Akt,Hsp90!1,S1177~U).Hsp90(eNOS!1,Akt) + Akt(Hsp90,eNOS,T~P) <->
eNOS(Akt,Hsp90!1,S1177~U).Hsp90(eNOS!1,Akt!2).Akt(Hsp90!2,eNOS,T~P) konpAkt_pHsp90,koffpAkt_pHsp90
eNOS(CaM,Akt!1,Hsp90,S1177~U).Akt(eNOS!1,Hsp90) + Hsp90(Akt,eNOS,Y300~P) <->
eNOS(CaM,Akt,Hsp90!1,S1177~U).Hsp90(eNOS!1,Akt!2,Y300~P).Akt(Hsp90!2,eNOS) konHsp90_eNOS,koffHsp90_eNOS

CaM(eNOS!3).eNOS(CaM!3,Akt!1,Hsp90,S1177~U).Akt(eNOS!1,Hsp90) + Hsp90(Akt,eNOS,Y300~P) <->
CaM(eNOS!3).eNOS(CaM!3,Akt,Hsp90!1,S1177~U).Hsp90(eNOS!1,Akt!2,Y300~P).Akt(Hsp90!2,eNOS)
konHsp90_CaMeNOS,koffHsp90_CaMeNOS

```
CaM(eNOS!3).eNOS(CaM!3,Akt,Hsp90,S1177~U) + Akt(Hsp90!1,eNOS).Hsp90(Akt!1,eNOS) <->
CaM(eNOS!3).eNOS(CaM!3,Akt,Hsp90!1,S1177~U).Hsp90(eNOS!1,Akt!2).Akt(Hsp90!2,eNOS)
        konHsp90_CaMeNOS,koffHsp90_CaMeNOS

#only CaM-bound eNOS can be phosphorylated
CaM(eNOS!1).eNOS(CaM!1,S1177~U,Hsp90,Akt!2).Akt(Hsp90,eNOS!2,T~P) -> CaM(eNOS!1).eNOS(CaM!1,S1177~P,Hsp90,Akt) +
Akt(Hsp90,eNOS,T~P)        kcat_pAkt
CaM(eNOS!1).eNOS(CaM!1,S1177~U,Hsp90!2,Akt).Akt(Hsp90!3,eNOS,T~P).Hsp90(eNOS!2,Akt!3,Y300~P) ->
CaM(eNOS!1).eNOS(CaM!1,S1177~P,Hsp90!2,Akt).Hsp90(eNOS!2,Akt,Y300~P) + Akt(Hsp90,eNOS,T~P)
        kcat_pAkt_H

# all peNOS forms have a dephosphorylation rate
eNOS(S1177~P) -> eNOS(S1177~U)      kdp_eNOS

# without Hsp90 present, peNOS is also subject to a degradation rate.
# eNOS unphosphorylated does not have a kdeg bc it doesnt have a ksyn. implicit balance b/w deg and syn here.
eNOS(S1177~P,Hsp90,Arg,Akt) -> Trash()                  kdeg_eNOS

Arg(eNOS) + eNOS(CaM,S1177~U,Arg) <-> eNOS(CaM,S1177~U,Arg!3).Arg(eNOS!3)       kon_beNOS_Arg,koff_beNOS_Arg
Arg(eNOS) + CaM(eNOS!2).eNOS(CaM!2,S1177~U,Arg,Hsp90) <->
CaM(eNOS!2).eNOS(CaM!2,S1177~U,Arg!3,Hsp90).Arg(eNOS!3)       kon_eNOS_Arg,koff_eNOS_Arg
CaM(eNOS!2).eNOS(CaM!2,S1177~U,Arg!3,Hsp90).Arg(eNOS!3) -> NO + Cit + CaM(eNOS!2).eNOS(CaM!2,S1177~U,Arg,Hsp90)
        kcat_eNOS

Arg(eNOS) + eNOS(S1177~P,Arg,Hsp90,Akt) <-> eNOS(S1177~P,Arg!3,Hsp90,Akt).Arg(eNOS!3)
kon_peNOS_Arg,koff_peNOS_Arg
eNOS(CaM,S1177~P,Arg!3,Hsp90,Akt).Arg(eNOS!3) -> NO + Cit + eNOS(CaM,S1177~P,Arg,Hsp90,Akt)
        kcat_peNOS
CaM(eNOS!1).eNOS(CaM!1,S1177~P,Arg!3,Hsp90,Akt).Arg(eNOS!3) -> NO + Cit +
CaM(eNOS!1).eNOS(CaM!1,S1177~P,Arg,Hsp90,Akt)         kcat_peNOS

Arg(eNOS) + CaM(eNOS!2).eNOS(CaM!2,S1177~U,Arg,Hsp90!1,Akt).Hsp90(eNOS!1) <->
CaM(eNOS!2).eNOS(CaM!2,S1177~U,Arg!3,Hsp90!1,Akt).Hsp90(eNOS!1).Arg(eNOS!3)     kon_eNOS_Arg_H,koff_eNOS_Arg_H
CaM(eNOS!2).eNOS(CaM!2,S1177~U,Arg!3,Hsp90!1,Akt).Hsp90(eNOS!1).Arg(eNOS!3) -> NO + Cit +
CaM(eNOS!2).eNOS(CaM!2,S1177~U,Arg,Hsp90!1,Akt).Hsp90(eNOS!1)                   kcat_eNOS_H

Arg(eNOS) + eNOS(S1177~P,Arg,Hsp90!1,Akt).Hsp90(eNOS!1) <->
eNOS(S1177~P,Arg!3,Hsp90!1,Akt).Hsp90(eNOS!1).Arg(eNOS!3) kon_peNOS_Arg_H,koff_peNOS_Arg_H
eNOS(CaM,S1177~P,Arg!3,Hsp90!1,Akt).Hsp90(eNOS!1).Arg(eNOS!3) -> NO + Cit +
eNOS(CaM,S1177~P,Arg,Hsp90!1,Akt).Hsp90(eNOS!1)                         kcat_peNOS_H
CaM(eNOS!2).eNOS(CaM!2,S1177~P,Arg!3,Hsp90!1,Akt).Hsp90(eNOS!1).Arg(eNOS!3) -> NO + Cit +
CaM(eNOS!2).eNOS(CaM!2,S1177~P,Arg,Hsp90!1,Akt).Hsp90(eNOS!1)                   kcat_peNOS_H

#################### END Hsp90 module #####################################
#################### START NO module #####################################
# NO activating sGC
sGC_b + NO <-> sGC_6c               kon1,koff1
sGC_6c <-> sGC_5c                   kon2,koff2
sGC_5c + NO <-> sGC_5c_NO           kon3,koff3
sGC_5c_NO <-> sGC_NO + NO                     kon4,koff4
sGC_NO <-> sGC + NO                 koff5,kon5
sGC <-> sGC_b                       koff6,kon6

NO -> NObuffer()                    kclearNO

GTP + sGC_5c -> cGMP + sGC_5c       kcat_sGC
cGMP+PDE <-> cGMP_PDE               kp1,kdp1
cGMP_PDE <-> cGMP_PDE_a             kp2,kdp2
```

```
cGMP -> GMP                                  RprodGMP
#################### END NO module ####################################


end reaction rules

########################################################################################################
########################################################################################################
########################################################################################################


end model

generate_network({overwrite=>1});
writeMfile({});
writeSBML({});
```
