# Supplementary material for: Mathematical Model Predicts Effective Strategies to Inhibit VEGF-eNOS Signaling
Source: J Clin Med. 2020 Apr 26;9(5):1255. doi: 10.3390/jcm9051255 (PMC7287924; doi:10.3390/jcm9051255)
Supplement: Supplementary file 1 [file jcm-09-01255-s001.zip › jcm-727669-US-SI-supplementary-for conversion 2/File S3 List of ODEs.pdf]

List of ODEs (160 total)

Biochemical species (158 total)

d(TSP1)/dt = - konTSP1VEGF*TSP1*VEGF - konTSP1CD47*TSP1*CD47mem + koffTSP1VEGF*TSP1:VEGF + koffTSP1CD47*TSP1:CD47mem ;

d(VEGF)/dt = - konTSP1VEGF*TSP1*VEGF - konVEGFR2*VEGF*R2mem + koffTSP1VEGF*TSP1:VEGF + koffVEGFR2*VEGF:R2mem - konVEGFR2*VEGF*CD47:R2mem + koffVEGFR2*VEGF:R2:CD47mem ;

d(R2mem)/dt = - konVEGFR2*VEGF*R2mem - konR2CD47*R2mem*CD47mem - kinterR2*R2mem + ksynR2*null + koffVEGFR2*VEGF:R2mem + koffR2CD47*CD47:R2mem ;

d(CD47mem)/dt = - konTSP1CD47*TSP1*CD47mem - konR2CD47*R2mem*CD47mem - kinterCD47*CD47mem + ksynCD47*null + koffTSP1CD47*TSP1:CD47mem - konR2CD47*VEGF:R2mem*CD47mem + koffR2CD47*CD47:R2mem - konR2CD47*VEGF:pR2mem*CD47mem + koffR2CD47*VEGF:R2:CD47mem + koffR2CD47*VEGF:pR2:CD47mem ;

d(Src)/dt = - konSrcHsp90*Src*Hsp90 - konSrcAkt*Src*Akt + koffSrcHsp90*Hsp90:Src + koffSrcAkt*Akt:Src - konSrcpR2*VEGF:pR2cyt*Src - konSrcpR2*VEGF:pR2:CD47cyt*Src + koffSrcpR2*VEGF:pR2cyt:Src + koffSrcpR2*VEGF:pR2cyt:CD47:Src + kdpSrc*pSrc ;

d(Hsp90)/dt = - konSrcHsp90*Src*Hsp90 - konSrcHsp90*Akt:Src*Hsp90 + koffSrcHsp90*Hsp90:Src + koffSrcHsp90*Akt:Hsp90:Src - konSrcHsp90*pSrc*Hsp90 - konSrcHsp90*pSrc:Akt*Hsp90 + koffSrcHsp90*pSrc:Hsp90 + koffSrcHsp90*pSrc:Hsp90:Akt + kdpHsp90*pHsp90 ;

d(Akt)/dt = - konSrcAkt*Src*Akt + koffSrcAkt*Akt:Src - konSrcAkt*pSrc*Akt + koffSrcAkt*pSrc:Akt + kdpAkt*pAkt ;

d(eNOS)/dt = - konbeNOSArg*Arg*eNOS - kon2CeNOS*C:CaM*eNOS - kon2NeNOS*N:CaM*eNOS + koffbeNOSArg*Arg:eNOS + koffCaMeNOS*C:CaM:eNOS + koffCaMeNOS*N:CaM:eNOS - kon4eNOS*CN:CaM*eNOS + koffCaMeNOS*CN:CaM:eNOS - konHsp90eNOS*eNOS*pHsp90 - konAkteNOS*eNOS*pAkt + koffHsp90eNOS*eNOS:pHsp90 + koffAkteNOS*eNOS:pAkt - konHsp90eNOS*eNOS*pAkt:pHsp90 - konHsp90eNOS*eNOS*pAkt:Hsp90 - konHsp90eNOS*eNOS*Akt:pHsp90 + koffHsp90eNOS*eNOS:pHsp90:pAkt - konHsp90eNOS*eNOS*Akt:Hsp90 + koffHsp90eNOS*eNOS:pHsp90:Akt + koffHsp90eNOS*eNOS:Hsp90:pAkt + kdpeNOS*peNOS + koffHsp90eNOS*eNOS:Hsp90:Akt ;

d(Ca)/dt = (((((VolER/Volcyto)*I_IP3R)*(Castore-Ca))*((IP3^3.8)/((IP3^3.8)+(KM_IP3R^3.8))))*((KiCa^3.8)/((KiCa^3.8)+(Ca^3.8))))*null + ((((-I_PMCA)*(Ca^1.4))/((KM_PMCA^1.4)+(Ca^1.4)))+Istim)*null + (((-I_SERCA)*((Ca/(KM_SERCA+Ca))^2))+(kleakER*((Castore-Ca)^2)))*null -2.0* konCa2C*Ca*Ca*CaM -2.0* konCa2N*Ca*Ca*CaM +2.0* koffCa2C*C:CaM +2.0* koffCa2N*N:CaM -2.0* konCa2C*Ca*Ca*N:CaM -2.0* konCa2N*Ca*Ca*C:CaM -2.0* konCa2C*Ca*Ca*C:CaM:eNOS -2.0* konCa2C*Ca*Ca*Arg:N:CaM:eNOS +2.0* koffCa2C*CN:CaM -2.0* konCa2N*Ca*Ca*C:CaM:eNOS -2.0* konCa2N*Ca*Ca*Arg:C:CaM:eNOS +2.0* koffCa2N*CN:CaM +2.0* koffCa2C*CN:CaM:eNOS +2.0* koffCa2C*Arg:CN:CaM:eNOS +2.0* koffCa2N*CN:CaM:eNOS +2.0* koffCa2N*Arg:CN:CaM:eNOS -2.0* konCa2C*Ca*Ca*N:CaM:eNOS:pHsp90 -2.0* konCa2C*Ca*Ca*Arg:N:CaM:eNOS:pHsp90 -2.0* konCa2C*Ca*Ca*N:CaM:eNOS:pAkt -2.0* konCa2C*Ca*Ca*Arg:N:CaM:eNOS:pAkt +2.0* koffCa2C*CN:CaM:eNOS:pHsp90 +2.0* koffCa2C*Arg:CN:CaM:eNOS:pHsp90 +2.0* koffCa2C*CN:CaM:eNOS:pAkt +2.0* koffCa2C*Arg:CN:CaM:eNOS:pAkt -2.0* konCa2N*Ca*Ca*C:CaM:eNOS:pHsp90 -2.0* konCa2N*Ca*Ca*Arg:C:CaM:eNOS:pHsp90 -2.0* konCa2N*Ca*Ca*C:CaM:eNOS:pAkt -2.0* konCa2N*Ca*Ca*Arg:C:CaM:eNOS:pAkt +2.0* koffCa2N*CN:CaM:eNOS:pHsp90 +2.0* koffCa2N*Arg:CN:CaM:eNOS:pHsp90 +2.0* koffCa2N*CN:CaM:eNOS:pAkt +2.0* koffCa2N*Arg:CN:CaM:eNOS:pAkt -2.0* konCa2C*Ca*Ca*N:CaM:eNOS:pHsp90:pAkt -2.0* konCa2C*Ca*Ca*Arg:N:CaM:eNOS:pHsp90:pAkt -2.0* konCa2C*Ca*Ca*N:CaM:peNOS -2.0* konCa2C*Ca*Ca*Arg:N:CaM:peNOS +2.0* koffCa2C*CN:CaM:eNOS:pHsp90:pAkt +2.0* koffCa2C*Arg:CN:CaM:eNOS:pHsp90:pAkt +2.0* koffCa2C*CN:CaM:peNOS +2.0* koffCa2C*Arg:CN:CaM:peNOS -2.0* konCa2N*Ca*Ca*C:CaM:eNOS:pHsp90:pAkt -2.0* konCa2N*Ca*Ca*Arg:C:CaM:eNOS:pHsp90:pAkt -2.0* konCa2N*Ca*Ca*C:CaM:peNOS -2.0* konCa2N*Ca*Ca*Arg:C:CaM:peNOS +2.0* koffCa2N*CN:CaM:eNOS:pHsp90:pAkt +2.0* koffCa2N*Arg:CN:CaM:eNOS:pHsp90:pAkt +2.0* koffCa2N*CN:CaM:peNOS +2.0* koffCa2N*Arg:CN:CaM:peNOS -2.0* konCa2C*Ca*Ca*N:CaM:eNOS:pHsp90:Akt -2.0* konCa2C*Ca*Ca*Arg:N:CaM:eNOS:pHsp90:Akt -2.0* konCa2C*Ca*Ca*N:CaM:peNOS:pHsp90 -2.0* konCa2C*Ca*Ca*Arg:N:CaM:peNOS:pHsp90 -2.0* konCa2C*Ca*Ca*N:CaM:eNOS:Hsp90:pAkt -2.0* konCa2C*Ca*Ca*Arg:N:CaM:eNOS:Hsp90:pAkt +2.0* koffCa2C*CN:CaM:eNOS:pHsp90:Akt +2.0*

koffCa2C*Arg:CN:CaM:eNOS:pHsp90:Akt +2.0* koffCa2C*CN:CaM:peNOS:pHsp90 +2.0* koffCa2C*Arg:CN:CaM:peNOS:pHsp90 +2.0* koffCa2C*CN:CaM:eNOS:Hsp90:pAkt +2.0* koffCa2C*Arg:CN:CaM:eNOS:Hsp90:pAkt -2.0* konCa2N*Ca*Ca*C:CaM:eNOS:pHsp90:Akt -2.0* konCa2N*Ca*Ca*Arg:C:CaM:eNOS:pHsp90:Akt -2.0* konCa2N*Ca*Ca*C:CaM:peNOS:pHsp90 -2.0* konCa2N*Ca*Ca*Arg:C:CaM:peNOS:pHsp90 -2.0* konCa2N*Ca*Ca*C:CaM:eNOS:Hsp90:pAkt -2.0* konCa2N*Ca*Ca*Arg:C:CaM:eNOS:Hsp90:pAkt +2.0* koffCa2N*CN:CaM:eNOS:pHsp90:Akt +2.0* koffCa2N*Arg:CN:CaM:eNOS:pHsp90:Akt +2.0* koffCa2N*CN:CaM:peNOS:pHsp90 +2.0* koffCa2N*Arg:CN:CaM:peNOS:pHsp90 +2.0* koffCa2N*CN:CaM:eNOS:Hsp90:pAkt +2.0* koffCa2N*Arg:CN:CaM:eNOS:Hsp90:pAkt -2.0* konCa2C*Ca*Ca*N:CaM:eNOS:Hsp90:Akt -2.0* konCa2C*Ca*Ca*Arg:N:CaM:eNOS:Hsp90:Akt -2.0* konCa2C*Ca*Ca*N:CaM:eNOS:Hsp90 -2.0* konCa2C*Ca*Ca*Arg:N:CaM:eNOS:Hsp90 -2.0* konCa2C*Ca*Ca*N:CaM:eNOS:Akt -2.0* konCa2C*Ca*Ca*Arg:N:CaM:eNOS:Akt +2.0* koffCa2C*CN:CaM:eNOS:Hsp90:Akt +2.0* koffCa2C*Arg:CN:CaM:eNOS:Hsp90:Akt +2.0* koffCa2C*CN:CaM:eNOS:Hsp90 +2.0* koffCa2C*Arg:CN:CaM:eNOS:Hsp90 +2.0* koffCa2C*CN:CaM:eNOS:Hsp90:Akt +2.0* koffCa2C*Arg:CN:CaM:eNOS:Hsp90:Akt -2.0* konCa2N*Ca*Ca*C:CaM:eNOS:Hsp90:Akt -2.0* konCa2N*Ca*Ca*Arg:C:CaM:eNOS:Hsp90:Akt -2.0* konCa2N*Ca*Ca*C:CaM:eNOS:Hsp90 -2.0* konCa2N*Ca*Ca*Arg:C:CaM:eNOS:Hsp90 -2.0* konCa2N*Ca*Ca*C:CaM:eNOS:Akt -2.0* konCa2N*Ca*Ca*Arg:C:CaM:eNOS:Akt +2.0* koffCa2N*CN:CaM:eNOS:Hsp90:Akt +2.0* koffCa2N*Arg:CN:CaM:eNOS:Hsp90:Akt +2.0* koffCa2N*CN:CaM:eNOS:Hsp90 +2.0* koffCa2N*Arg:CN:CaM:eNOS:Hsp90 +2.0* koffCa2N*CN:CaM:eNOS:Hsp90:Akt +2.0* koffCa2N*Arg:CN:CaM:eNOS:Hsp90:Akt ;

d(CaM)/dt = - konCa2C*Ca*Ca*CaM - konCa2N*Ca*Ca*CaM + koffCa2C*C:CaM + koffCa2N*N:CaM ;

d(Arg)/dt = - konbeNOSArg*Arg*eNOS + koffbeNOSArg*Arg:eNOS - koneNOSArg*Arg*C:CaM:eNOS - koneNOSArg*Arg*N:CaM:eNOS + koffeNOSArg*Arg:C:CaM:eNOS + koffeNOSArg*Arg:N:CaM:eNOS - koneNOSArg*Arg*CN:CaM:eNOS + koffeNOSArg*Arg:CN:CaM:eNOS - konbeNOSArg*Arg*eNOS:pHsp90 - konbeNOSArg*Arg*eNOS:pAkt + koffbeNOSArg*Arg:eNOS:pHsp90 + koffbeNOSArg*Arg:eNOS:pAkt - koneNOSArg*Arg*C:CaM:eNOS:pAkt - koneNOSArg*Arg*N:CaM:eNOS:pAkt - koneNOSArg*Arg*CN:CaM:eNOS:pAkt + koffeNOSArg*Arg:C:CaM:eNOS:pAkt + koffeNOSArg*Arg:N:CaM:eNOS:pAkt + koffeNOSArg*Arg:CN:CaM:eNOS:pAkt - koneNOSArg_H*Arg*C:CaM:eNOS:pHsp90 - koneNOSArg_H*Arg*N:CaM:eNOS:pHsp90 - koneNOSArg_H*Arg*CN:CaM:eNOS:pHsp90 + koffeNOSArg_H*Arg:C:CaM:eNOS:pHsp90 + koffeNOSArg_H*Arg:N:CaM:eNOS:pHsp90 + koffeNOSArg_H*Arg:CN:CaM:eNOS:pHsp90 - konbeNOSArg*Arg*eNOS:pHsp90:pAkt + koffbeNOSArg*Arg:eNOS:pHsp90:pAkt - konpeNOSArg*Arg*C:CaM:peNOS - konpeNOSArg*Arg*N:CaM:peNOS - konpeNOSArg*Arg*CN:CaM:peNOS + koffpeNOSArg*Arg:C:CaM:peNOS + koffpeNOSArg*Arg:N:CaM:peNOS + koffpeNOSArg*Arg:CN:CaM:peNOS - koneNOSArg_H*Arg*C:CaM:eNOS:pHsp90:pAkt - koneNOSArg_H*Arg*N:CaM:eNOS:pHsp90:pAkt - koneNOSArg_H*Arg*CN:CaM:eNOS:pHsp90:pAkt + koffeNOSArg_H*Arg:C:CaM:eNOS:pHsp90:pAkt + koffeNOSArg_H*Arg:N:CaM:eNOS:pHsp90:pAkt + koffeNOSArg_H*Arg:CN:CaM:eNOS:pHsp90:pAkt - konbeNOSArg*Arg*eNOS:pHsp90:Akt - konbeNOSArg*Arg*eNOS:Hsp90:pAkt + koffbeNOSArg*Arg:eNOS:pHsp90:Akt + koffbeNOSArg*Arg:eNOS:Hsp90:pAkt - konpeNOSArg*Arg*peNOS + koffpeNOSArg*Arg:peNOS - koneNOSArg_H*Arg*C:CaM:eNOS:pHsp90:Akt - koneNOSArg_H*Arg*N:CaM:eNOS:pHsp90:Akt - koneNOSArg_H*Arg*CN:CaM:eNOS:pHsp90:Akt - koneNOSArg_H*Arg*C:CaM:eNOS:Hsp90:pAkt - koneNOSArg_H*Arg*N:CaM:eNOS:Hsp90:pAkt - koneNOSArg_H*Arg*CN:CaM:eNOS:Hsp90:pAkt + koffeNOSArg_H*Arg:C:CaM:eNOS:pHsp90:Akt + koffeNOSArg_H*Arg:N:CaM:eNOS:pHsp90:Akt + koffeNOSArg_H*Arg:CN:CaM:eNOS:pHsp90:Akt + koffeNOSArg_H*Arg:C:CaM:eNOS:Hsp90:pAkt + koffeNOSArg_H*Arg:N:CaM:eNOS:Hsp90:pAkt + koffeNOSArg_H*Arg:CN:CaM:eNOS:Hsp90:pAkt - konpeNOSArg_H*Arg*C:CaM:peNOS:pHsp90 - konpeNOSArg_H*Arg*N:CaM:peNOS:pHsp90 - konpeNOSArg_H*Arg*CN:CaM:peNOS:pHsp90 + koffpeNOSArg_H*Arg:C:CaM:peNOS:pHsp90 + koffpeNOSArg_H*Arg:N:CaM:peNOS:pHsp90 + koffpeNOSArg_H*Arg:CN:CaM:peNOS:pHsp90 - konbeNOSArg*Arg*eNOS:Hsp90:Akt - konbeNOSArg*Arg*eNOS:Hsp90 - konbeNOSArg*Arg*Akt:eNOS + koffbeNOSArg*Arg:eNOS:Hsp90:Akt + koffbeNOSArg*Arg:eNOS:Hsp90 + koffbeNOSArg*Arg:Akt:eNOS - koneNOSArg*Arg*C:CaM:eNOS:Akt - koneNOSArg*Arg*N:CaM:eNOS:Akt - koneNOSArg*Arg*CN:CaM:eNOS:Hsp90:Akt + koffeNOSArg*Arg:C:CaM:eNOS:Akt + koffeNOSArg*Arg:N:CaM:eNOS:Akt + koffeNOSArg*Arg:CN:CaM:eNOS:Hsp90:Akt - koneNOSArg_H*Arg*C:CaM:eNOS:Hsp90:Akt - koneNOSArg_H*Arg*N:CaM:eNOS:Hsp90:Akt - koneNOSArg_H*Arg*CN:CaM:eNOS:Hsp90:Akt - koneNOSArg_H*Arg*C:CaM:eNOS:Hsp90 - koneNOSArg_H*Arg*N:CaM:eNOS:Hsp90 - koneNOSArg_H*Arg*CN:CaM:eNOS:Hsp90 +

koffeNOSArg_H*Arg:C:CaM:eNOS:Hsp90:Akt + koffeNOSArg_H*Arg:N:CaM:eNOS:Hsp90:Akt + koffeNOSArg_H*Arg:CN:CaM:eNOS:Hsp90:Akt + koffeNOSArg_H*Arg:C:CaM:eNOS:Hsp90 + koffeNOSArg_H*Arg:N:CaM:eNOS:Hsp90 + koffeNOSArg_H*Arg:CN:CaM:eNOS:Hsp90 - konpeNOSArg_H*Arg*peNOS:pHsp90 + koffpeNOSArg_H*Arg:peNOS:pHsp90 ;

d(sGC_b)/dt = - kf6*sGC_b + kr6*sGC - konNOsGC*sGC_b*NO + koffNOsGC*sGC_6c ;

d(GTP)/dt = - kcatsGC*GTP*sGC_5c ;

d(NObuffer)/dt = kclearNO*NO ;

d(PIP2)/dt = ksynPIP2*null - RF2(parameters,observables)*pPLCg*PIP2 ;

d(PLCg)/dt = - (kpPLCg/(KM_PLCgR2+PLCg))*PLCg*VEGF:pR2mem - (kpPLCg/(KM_PLCgR2+PLCg))*PLCg*VEGF:pR2:CD47mem - (kpPLCg/(KM_PLCgR2+PLCg))*PLCg*VEGF:pR2cyt + kdpPLCg*pPLCg - (kpPLCg/(KM_PLCgR2+PLCg))*PLCg*VEGF:pR2:CD47cyt - (kpPLCg/(KM_PLCgR2+PLCg))*PLCg*VEGF:pR2cyt:Src - (kpPLCg/(KM_PLCgR2+PLCg))*PLCg*VEGF:pR2cyt:Src:Hsp90 - (kpPLCg/(KM_PLCgR2+PLCg))*PLCg*VEGF:pR2cyt:Src:Akt - (kpPLCg/(KM_PLCgR2+PLCg))*PLCg*VEGF:pR2cyt:Src:Akt:Hsp90 - (kpPLCg/(KM_PLCgR2+PLCg))*PLCg*VEGF:pR2cyt:CD47:Src - (kpPLCg/(KM_PLCgR2+PLCg))*PLCg*VEGF:pR2cyt:CD47:Src:Hsp90 - (kpPLCg/(KM_PLCgR2+PLCg))*PLCg*VEGF:pR2cyt:CD47:Src:Akt - (kpPLCg/(KM_PLCgR2+PLCg))*PLCg*VEGF:pR2cyt:CD47:Src:Akt:Hsp90 ;

d(Istim)/dt = (((((((Volext/Volcyto)*Ca_ext)-Ca)*ICRAC)*((KCRAC^n_CRAC)/((KCRAC^n_CRAC)+(Castore^n_CRAC))))/t_stim)-(Istim/t_stim))*null ;

d(Castore)/dt = (((((-I_IP3R)*(Castore-Ca))*((IP3^3.8)/((IP3^3.8)+(KM_IP3R^3.8))))*((KiCa^3.8)/((KiCa^3.8)+(Ca^3.8))))*(1/(1+(CSQN/((K_CSQN+Castore)^2)))))*null + (((I_SERCA*((Ca/(KM_SERCA+Ca))^2))*(Volcyto/VolER))*(1/(1+(CSQN/((K_CSQN+Castore)^2))))); *null + ((((-kleakER)*(Volcyto/VolER))*((Castore-Ca)^2))*(1/(1+(CSQN/((K_CSQN+Castore)^2))))); *null ;

d(PDE)/dt = - koncGMPPDE*cGMP*PDE + koffcGMPPDE*cGMP:PDE ;

d(TSP1:VEGF)/dt = konTSP1VEGF*TSP1*VEGF - koffTSP1VEGF*TSP1:VEGF - kdegVEGF*TSP1:VEGF ;

d(VEGF:R2mem)/dt = konVEGFR2*VEGF*R2mem - koffVEGFR2*VEGF:R2mem - konR2CD47*VEGF:R2mem*CD47mem - kinterR2*VEGF:R2mem - kpR2*VEGF:R2mem + koffR2CD47*VEGF:R2:CD47mem + kdpR2*VEGF:pR2mem ;

d(TSP1:CD47mem)/dt = konTSP1CD47*TSP1*CD47mem - koffTSP1CD47*TSP1:CD47mem - kinterTSP1bd*TSP1:CD47mem ;

d(CD47:R2mem)/dt = konR2CD47*R2mem*CD47mem - konVEGFR2*VEGF*CD47:R2mem - koffR2CD47*CD47:R2mem - kinterR2CD47*CD47:R2mem + koffVEGFR2*VEGF:R2:CD47mem ;

d(R2cyt)/dt = kinterR2*R2mem - kdegR2*R2cyt ;

d(CD47cyt)/dt = kinterCD47*CD47mem - kdegCD47*CD47cyt ;

d(Hsp90:Src)/dt = konSrcHsp90*Src*Hsp90 - koffSrcHsp90*Hsp90:Src - konSrcpR2*VEGF:pR2cyt*Hsp90:Src - konSrcpR2*VEGF:pR2:CD47cyt*Hsp90:Src + koffSrcpR2*VEGF:pR2cyt:Src:Hsp90 + koffSrcpR2*VEGF:pR2cyt:CD47:Src:Hsp90 + kdpSrc*pSrc:Hsp90 ;

d(Akt:Src)/dt = konSrcAkt*Src*Akt - konSrcHsp90*Akt:Src*Hsp90 - koffSrcAkt*Akt:Src + koffSrcHsp90*Akt:Hsp90:Src - konSrcpR2*VEGF:pR2cyt*Akt:Src - konSrcpR2*VEGF:pR2:CD47cyt*Akt:Src + koffSrcpR2*VEGF:pR2cyt:Src:Akt + koffSrcpR2*VEGF:pR2cyt:CD47:Src:Akt + kdpSrc*pSrc:Akt ;

d(C:CaM)/dt = konCa2C*Ca*Ca*CaM - koffCa2C*C:CaM - konCa2N*Ca*Ca*C:CaM - kon2CeNOS*C:CaM*eNOS - kon2CeNOS*C:CaM*Arg:eNOS + koffCa2N*CN:CaM + koffCaMeNOS*C:CaM:eNOS + koffCaMeNOS*Arg:C:CaM:eNOS - kon2CeNOS_H*C:CaM*eNOS:pHsp90 - kon2CeNOS_H*C:CaM*Arg:eNOS:pHsp90 + koffCaMeNOS_H*C:CaM:eNOS:pHsp90 + koffCaMeNOS_H*Arg:C:CaM:eNOS:pHsp90 - kon2CpeNOS*C:CaM*eNOS:pAkt - kon2CpeNOS*C:CaM*Arg:eNOS:pAkt + koffCaMpeNOS*C:CaM:eNOS:pAkt + koffCaMpeNOS*Arg:C:CaM:eNOS:pAkt - kon2CeNOS_H*C:CaM*eNOS:pHsp90:pAkt - kon2CeNOS_H*C:CaM*Arg:eNOS:pHsp90:pAkt + koffCaMeNOS_H*C:CaM:eNOS:pHsp90:pAkt + koffCaMeNOS_H*Arg:C:CaM:eNOS:pHsp90:pAkt + koffCaMpeNOS*C:CaM:peNOS + koffCaMpeNOS*Arg:C:CaM:peNOS - kon2CpeNOS_H*C:CaM*eNOS:pHsp90:pAkt - kon2CpeNOS_H*C:CaM*Arg:eNOS:pHsp90:pAkt + koffCaMpeNOS_H*C:CaM:eNOS:pHsp90:pAkt +

koffCaMpeNOS_H*Arg:C:CaM:eNOS:pHsp90:pAkt - kon2CeNOS_H*C:CaM*eNOS:pHsp90:Akt -
kon2CeNOS_H*C:CaM*Arg:eNOS:pHsp90:Akt - kon2CeNOS_H*C:CaM*eNOS:Hsp90:pAkt -
kon2CeNOS_H*C:CaM*Arg:eNOS:Hsp90:pAkt + koffCaMeNOS_H*C:CaM:eNOS:pHsp90:Akt +
koffCaMeNOS_H*Arg:C:CaM:eNOS:pHsp90:Akt + koffCaMeNOS_H*C:CaM:eNOS:Hsp90:pAkt +
koffCaMeNOS_H*Arg:C:CaM:eNOS:Hsp90:pAkt - kon2CpeNOS*C:CaM*peNOS - kon2CpeNOS*C:CaM*Arg:peNOS +
koffCaMpeNOS_H*C:CaM:peNOS:pHsp90 + koffCaMpeNOS_H*Arg:C:CaM:peNOS:pHsp90 -
kon2CpeNOS_H*C:CaM*eNOS:pHsp90:Akt - kon2CpeNOS_H*C:CaM*Arg:eNOS:pHsp90:Akt +
koffCaMpeNOS_H*C:CaM:eNOS:pHsp90:Akt + koffCaMpeNOS_H*Arg:C:CaM:eNOS:pHsp90:Akt -
kon2CeNOS_H*C:CaM*eNOS:Hsp90:Akt - kon2CeNOS_H*C:CaM*Arg:eNOS:Hsp90:Akt -
kon2CeNOS_H*C:CaM*eNOS:Hsp90 - kon2CeNOS_H*C:CaM*Arg:eNOS:Hsp90 +
koffCaMeNOS_H*C:CaM:eNOS:Hsp90:Akt + koffCaMeNOS_H*Arg:C:CaM:eNOS:Hsp90:Akt +
koffCaMeNOS_H*C:CaM:eNOS:Hsp90 + koffCaMeNOS_H*Arg:C:CaM:eNOS:Hsp90 -
kon2CpeNOS_H*C:CaM*peNOS:pHsp90 - kon2CpeNOS_H*C:CaM*Arg:peNOS:pHsp90 -
kon2CpeNOS*C:CaM*Akt:eNOS - kon2CpeNOS*C:CaM*Arg:Akt:eNOS + koffCaMpeNOS*C:CaM:eNOS:Akt +
koffCaMpeNOS*Arg:C:CaM:eNOS:Akt ;

d(N:CaM)/dt = konCa2N*Ca*Ca*CaM - koffCa2N*N:CaM - konCa2C*Ca*Ca*N:CaM - kon2NeNOS*N:CaM*eNOS -
kon2NeNOS*N:CaM*Arg:eNOS + koffCa2C*CN:CaM + koffCaMeNOS*C:CaM:eNOS +
koffCaMeNOS*Arg:N:CaM:eNOS - kon2NeNOS_H*N:CaM*eNOS:pHsp90 - kon2NeNOS_H*N:CaM*Arg:eNOS:pHsp90
+ koffCaMeNOS_H*N:CaM:eNOS:pHsp90 + koffCaMeNOS_H*Arg:N:CaM:eNOS:pHsp90 -
kon2NpeNOS*N:CaM*eNOS:pAkt - kon2NpeNOS*N:CaM*Arg:eNOS:pAkt + koffCaMpeNOS*N:CaM:eNOS:pAkt +
koffCaMpeNOS*Arg:N:CaM:eNOS:pAkt - kon2NeNOS_H*N:CaM*eNOS:pHsp90:pAkt -
kon2NeNOS_H*N:CaM*Arg:eNOS:pHsp90:pAkt + kon2NeNOS_H*N:CaM*Arg:eNOS:pHsp90:pAkt +
koffCaMeNOS_H*Arg:N:CaM:eNOS:pHsp90:pAkt + koffCaMpeNOS*N:CaM:peNOS +
koffCaMpeNOS*Arg:N:CaM:peNOS - kon2NpeNOS_H*N:CaM*eNOS:pHsp90:pAkt -
kon2NpeNOS_H*N:CaM*Arg:eNOS:pHsp90:pAkt + koffCaMpeNOS_H*N:CaM:eNOS:pHsp90:pAkt +
koffCaMpeNOS_H*Arg:N:CaM:eNOS:pHsp90:pAkt - kon2NeNOS_H*N:CaM*eNOS:pHsp90:Akt -
kon2NeNOS_H*N:CaM*Arg:eNOS:pHsp90:Akt - kon2NeNOS_H*N:CaM*eNOS:Hsp90:pAkt -
kon2NeNOS_H*N:CaM*Arg:eNOS:Hsp90:pAkt + koffCaMeNOS_H*N:CaM:eNOS:pHsp90:Akt +
koffCaMeNOS_H*Arg:N:CaM:eNOS:pHsp90:Akt + koffCaMeNOS_H*N:CaM:eNOS:Hsp90:pAkt +
koffCaMeNOS_H*Arg:N:CaM:eNOS:Hsp90:pAkt - kon2NpeNOS*N:CaM*peNOS - kon2NpeNOS*N:CaM*Arg:peNOS +
koffCaMpeNOS_H*N:CaM:peNOS:pHsp90 + koffCaMpeNOS_H*Arg:N:CaM:peNOS:pHsp90 -
kon2NpeNOS_H*N:CaM*eNOS:pHsp90:Akt - kon2NpeNOS_H*N:CaM*Arg:eNOS:pHsp90:Akt +
koffCaMpeNOS_H*N:CaM:eNOS:pHsp90:Akt + koffCaMpeNOS_H*Arg:N:CaM:eNOS:pHsp90:Akt -
kon2NeNOS_H*N:CaM*eNOS:Hsp90:Akt - kon2NeNOS_H*N:CaM*Arg:eNOS:Hsp90:Akt -
kon2NeNOS_H*N:CaM*eNOS:Hsp90 - kon2NeNOS_H*N:CaM*Arg:eNOS:Hsp90 +
koffCaMeNOS_H*N:CaM:eNOS:Hsp90:Akt + koffCaMeNOS_H*Arg:N:CaM:eNOS:Hsp90:Akt +
koffCaMeNOS_H*N:CaM:eNOS:Hsp90 + koffCaMeNOS_H*Arg:N:CaM:eNOS:Hsp90 -
kon2NpeNOS_H*N:CaM*peNOS:pHsp90 - kon2NpeNOS_H*N:CaM*Arg:peNOS:pHsp90 -
kon2NpeNOS*N:CaM*Akt:eNOS - kon2NpeNOS*N:CaM*Arg:Akt:eNOS + koffCaMpeNOS*N:CaM:eNOS:Akt +
koffCaMpeNOS*Arg:N:CaM:eNOS:Akt ;

d(Arg:eNOS)/dt = konbeNOSArg*Arg*eNOS - kon2CeNOS*C:CaM*Arg:eNOS - kon2NeNOS*N:CaM*Arg:eNOS -
koffbeNOSArg*Arg:eNOS + koffCaMeNOS*Arg:C:CaM:eNOS + koffCaMeNOS*Arg:N:CaM:eNOS -
kon4eNOS*CN:CaM*Arg:eNOS + koffCaMeNOS*Arg:CN:CaM:eNOS - konHsp90eNOS*Arg:eNOS*pHsp90 -
konAkteNOS*Arg:eNOS*pAkt + koffHsp90eNOS*Arg:eNOS:pHsp90 + koffAkteNOS*Arg:eNOS:pAkt -
konHsp90eNOS*Arg:eNOS*pAkt:pHsp90 - konHsp90eNOS*Arg:eNOS*pAkt:Hsp90 -
konHsp90eNOS*Arg:eNOS*Akt:pHsp90 + koffHsp90eNOS*Arg:eNOS:pHsp90:pAkt -
konHsp90eNOS*Arg:eNOS*Akt:Hsp90 + koffHsp90eNOS*Arg:eNOS:pHsp90:Akt +
koffHsp90eNOS*Arg:eNOS:Hsp90:pAkt + kdpeNOS*Arg:peNOS + koffHsp90eNOS*Arg:eNOS:Hsp90:Akt ;

d(sGC)/dt = kf6*sGC_b - kr6*sGC - kf_GC_NO*sGC*NO + kr_GC_NO*sGC:NO ;

d(VEGF:R2:CD47mem)/dt = konVEGFR2*VEGF*CD47:R2mem + konR2CD47*VEGF:R2mem*CD47mem -
koffVEGFR2*VEGF:R2:CD47mem - koffR2CD47*VEGF:R2:CD47mem - kinterR2CD47*VEGF:R2:CD47mem -
kpR2*VEGF:R2:CD47mem + kdpR2CD47bd*VEGF:pR2:CD47mem ;

d(VEGF:R2cyt)/dt = kinterR2*VEGF:R2mem - kpR2*VEGF:R2cyt - kdegR2*VEGF:R2cyt + kdpR2*VEGF:pR2cyt ;

d(CD47:R2cyt)/dt = kinterR2CD47*CD47:R2mem - kdegCD47*CD47:R2cyt ;

d(TSP1:CD47cyt)/dt = kinterTSP1bd*TSP1:CD47mem - kdegCD47*TSP1:CD47cyt ;

d(VEGF:pR2mem)/dt = kpR2*VEGF:R2mem - konR2CD47*VEGF:pR2mem*CD47mem - kinterpR2*VEGF:pR2mem - kdpR2*VEGF:pR2mem + koffR2CD47*VEGF:pR2:CD47mem ;

d(Akt:Hsp90:Src)/dt = konSrcHsp90*Akt:Src*Hsp90 - koffSrcHsp90*Akt:Hsp90:Src - konSrcpR2*VEGF:pR2cyt*Akt:Hsp90:Src - konSrcpR2*VEGF:pR2:CD47cyt*Akt:Hsp90:Src + koffSrcpR2*VEGF:pR2cyt:Src:Akt:Hsp90 + koffSrcpR2*VEGF:pR2cyt:CD47:Src:Akt:Hsp90 + kdpSrc*pSrc:Hsp90:Akt ;

d(CN:CaM)/dt = konCa2C*Ca*Ca*N:CaM + konCa2N*Ca*Ca*C:CaM - koffCa2C*CN:CaM - koffCa2N*CN:CaM - kon4eNOS*CN:CaM*eNOS - kon4eNOS*CN:CaM*Arg:eNOS + koffCaMeNOS*CN:CaM:eNOS + koffCaMeNOS*Arg:CN:CaM:eNOS - kon4eNOS_H*CN:CaM*eNOS:pHsp90 - kon4eNOS_H*CN:CaM*Arg:eNOS:pHsp90 + koffCaMeNOS_H*CN:CaM:eNOS:pHsp90 + koffCaMeNOS_H*Arg:CN:CaM:eNOS:pHsp90 - kon4peNOS*CN:CaM*eNOS:pAkt - kon4peNOS*CN:CaM*Arg:eNOS:pAkt + koffCaMpeNOS*CN:CaM:eNOS:pAkt + koffCaMpeNOS*Arg:CN:CaM:eNOS:pAkt - kon4eNOS_H*CN:CaM*eNOS:pHsp90:pAkt - kon4eNOS_H*CN:CaM*Arg:eNOS:pHsp90:pAkt + koffCaMeNOS_H*CN:CaM:eNOS:pHsp90:pAkt + koffCaMeNOS_H*Arg:CN:CaM:eNOS:pHsp90:pAkt + koffCaMpeNOS*CN:CaM:peNOS + koffCaMpeNOS*CN:CaM:peNOS - kon4peNOS_H*CN:CaM*eNOS:pHsp90:pAkt - kon4peNOS_H*CN:CaM*Arg:eNOS:pHsp90:pAkt + koffCaMpeNOS_H*CN:CaM:eNOS:pHsp90:pAkt + koffCaMpeNOS_H*Arg:CN:CaM:eNOS:pHsp90:pAkt - kon4eNOS_H*CN:CaM*eNOS:pHsp90:Akt - kon4eNOS_H*CN:CaM*Arg:eNOS:pHsp90:Akt - kon4eNOS_H*CN:CaM*eNOS:Hsp90:pAkt - kon4eNOS_H*CN:CaM*Arg:eNOS:Hsp90:pAkt + koffCaMeNOS_H*CN:CaM:eNOS:pHsp90:Akt + koffCaMeNOS_H*Arg:CN:CaM:eNOS:pHsp90:Akt + koffCaMeNOS_H*CN:CaM:eNOS:Hsp90:pAkt + koffCaMeNOS_H*Arg:CN:CaM:eNOS:Hsp90:pAkt - kon4peNOS*CN:CaM*peNOS - kon4peNOS*CN:CaM*Arg:peNOS + koffCaMpeNOS_H*CN:CaM:peNOS:pHsp90 + koffCaMpeNOS_H*Arg:CN:CaM:peNOS:pHsp90 - kon4peNOS_H*CN:CaM*eNOS:pHsp90:Akt - kon4peNOS_H*CN:CaM*Arg:eNOS:pHsp90:Akt + koffCaMpeNOS_H*CN:CaM:eNOS:pHsp90:Akt + koffCaMpeNOS_H*Arg:CN:CaM:eNOS:pHsp90:Akt - kon4eNOS_H*CN:CaM*eNOS:Hsp90:Akt - kon4eNOS_H*CN:CaM*Arg:eNOS:Hsp90:Akt - kon4eNOS_H*CN:CaM*eNOS:Hsp90 - kon4eNOS_H*CN:CaM*Arg:eNOS:Hsp90 + koffCaMeNOS_H*CN:CaM:eNOS:Hsp90:Akt + koffCaMeNOS_H*Arg:CN:CaM:eNOS:Hsp90:Akt + koffCaMeNOS_H*CN:CaM:eNOS:Hsp90 + koffCaMeNOS_H*Arg:CN:CaM:eNOS:Hsp90 - kon4peNOS_H*CN:CaM*peNOS:pHsp90 - kon4peNOS_H*CN:CaM*Arg:peNOS:pHsp90 - kon4peNOS*CN:CaM*Akt:eNOS - kon4peNOS*CN:CaM*Arg:Akt:eNOS + koffCaMpeNOS*CN:CaM:eNOS:Hsp90:Akt + koffCaMpeNOS*Arg:CN:CaM:eNOS:Hsp90:Akt ;

d(C:CaM:eNOS)/dt = kon2CeNOS*C:CaM*eNOS - konCa2N*Ca*Ca*C:CaM:eNOS - koffCaMeNOS*C:CaM:eNOS - koneNOSArg*Arg*C:CaM:eNOS + koffeNOSArg*Arg:C:CaM:eNOS + kcateNOS*Arg:C:CaM:eNOS + koffCa2N*CN:CaM:eNOS - konHsp90_CaMeNOS*C:CaM:eNOS*pHsp90 - konAkteNOS*C:CaM:eNOS*pAkt + koffHsp90_CaMeNOS*C:CaM:eNOS:pHsp90 + koffAkteNOS*C:CaM:eNOS:pAkt - konHsp90_CaMeNOS*C:CaM:eNOS*pAkt:pHsp90 - konHsp90_CaMeNOS*C:CaM:eNOS*pAkt:Hsp90 - konHsp90_CaMeNOS*C:CaM:eNOS*Akt:pHsp90 + koffHsp90_CaMeNOS*C:CaM:eNOS:pHsp90:pAkt + kdpeNOS*C:CaM:peNOS - konHsp90_CaMeNOS*C:CaM:eNOS*Akt:Hsp90 + koffHsp90_CaMeNOS*C:CaM:eNOS:pHsp90:Akt + koffHsp90_CaMeNOS*C:CaM:eNOS:Hsp90:pAkt + koffHsp90_CaMeNOS*C:CaM:eNOS:Hsp90:Akt ;

d(Arg:C:CaM:eNOS)/dt = kon2CeNOS*C:CaM*Arg:eNOS - konCa2N*Ca*Ca*Arg:C:CaM:eNOS - koffCaMeNOS*Arg:C:CaM:eNOS + koneNOSArg*Arg*C:CaM:eNOS - koffeNOSArg*Arg:C:CaM:eNOS - kcateNOS*Arg:C:CaM:eNOS + koffCa2N*Arg:CN:CaM:eNOS - konHsp90_CaMeNOS*Arg:C:CaM:eNOS*pHsp90 - konAkteNOS*Arg:C:CaM:eNOS*pAkt + koffHsp90_CaMeNOS*Arg:C:CaM:eNOS:pHsp90 + koffAkteNOS*Arg:C:CaM:eNOS:pAkt - konHsp90_CaMeNOS*Arg:C:CaM:eNOS*pAkt:pHsp90 - konHsp90_CaMeNOS*Arg:C:CaM:eNOS*pAkt:Hsp90 - konHsp90_CaMeNOS*Arg:C:CaM:eNOS*Akt:pHsp90 + koffHsp90_CaMeNOS*Arg:C:CaM:eNOS:pHsp90:pAkt + kdpeNOS*Arg:C:CaM:peNOS - konHsp90_CaMeNOS*Arg:C:CaM:eNOS*Akt:Hsp90 + koffHsp90_CaMeNOS*Arg:C:CaM:eNOS:pHsp90:Akt + koffHsp90_CaMeNOS*Arg:C:CaM:eNOS:Hsp90:pAkt + koffHsp90_CaMeNOS*Arg:C:CaM:eNOS:Hsp90:Akt ;

d(C:CaM:eNOS)/dt = kon2NeNOS*N:CaM*eNOS - konCa2C*Ca*Ca*C:CaM:eNOS - koffCaMeNOS*C:CaM:eNOS - koneNOSArg*Arg*N:CaM:eNOS + koffeNOSArg*Arg:N:CaM:eNOS + kcateNOS*Arg:N:CaM:eNOS + koffCa2C*CN:CaM:eNOS - konHsp90_CaMeNOS*N:CaM:eNOS*pHsp90 - konAkteNOS*N:CaM:eNOS*pAkt + koffHsp90_CaMeNOS*N:CaM:eNOS:pHsp90 + koffAkteNOS*N:CaM:eNOS:pAkt - konHsp90_CaMeNOS*N:CaM:eNOS*pAkt:pHsp90 - konHsp90_CaMeNOS*N:CaM:eNOS*pAkt:Hsp90 - konHsp90_CaMeNOS*N:CaM:eNOS*Akt:pHsp90 + koffHsp90_CaMeNOS*N:CaM:eNOS:pHsp90:pAkt + kdpeNOS*N:CaM:peNOS - konHsp90_CaMeNOS*N:CaM:eNOS*Akt:Hsp90 +

koffHsp90_CaMeNOS*N:CaM:eNOS:pHsp90:Akt + koffHsp90_CaMeNOS*N:CaM:eNOS:Hsp90:pAkt +
koffHsp90_CaMeNOS*N:CaM:eNOS:Hsp90:Akt ;

d(Arg:N:CaM:eNOS)/dt = kon2NeNOS*N:CaM*Arg:eNOS - konCa2C*Ca*Ca*Arg:N:CaM:eNOS -
koffCaMeNOS*Arg:N:CaM:eNOS + koneNOSArg*Arg*N:CaM:eNOS - koffeNOSArg*Arg:N:CaM:eNOS -
kcateNOS*Arg:N:CaM:eNOS + koffCa2C*Arg:CN:CaM:eNOS - konHsp90_CaMeNOS*Arg:N:CaM:eNOS*pHsp90 -
konAkteNOS*Arg:N:CaM:eNOS*pAkt + koffHsp90_CaMeNOS*Arg:N:CaM:eNOS:pHsp90 +
koffAkteNOS*Arg:N:CaM:eNOS:pAkt - konHsp90_CaMeNOS*Arg:N:CaM:eNOS*pAkt:pHsp90 -
konHsp90_CaMeNOS*Arg:N:CaM:eNOS*pAkt:Hsp90 - konHsp90_CaMeNOS*Arg:N:CaM:eNOS*Akt:pHsp90 +
koffHsp90_CaMeNOS*Arg:N:CaM:eNOS:pHsp90:pAkt + kdpeNOS*Arg:N:CaM:peNOS -
konHsp90_CaMeNOS*Arg:N:CaM:eNOS*Akt:Hsp90 + koffHsp90_CaMeNOS*Arg:N:CaM:eNOS:pHsp90:Akt +
koffHsp90_CaMeNOS*Arg:N:CaM:eNOS:Hsp90:pAkt + koffHsp90_CaMeNOS*Arg:N:CaM:eNOS:Hsp90:Akt ;

d(VEGF:pR2:CD47mem)/dt = konR2CD47*VEGF:pR2mem*CD47mem + kpR2*VEGF:R2:CD47mem -
koffR2CD47*VEGF:pR2:CD47mem - kinterpR2CD47*VEGF:pR2:CD47mem - kdpR2CD47bd*VEGF:pR2:CD47mem ;

d(VEGF:pR2cyt)/dt = kinterpR2*VEGF:pR2mem + kpR2*VEGF:R2cyt - kdpR2*VEGF:pR2cyt - kdegR2*VEGF:pR2cyt -
kdegpR2*VEGF:pR2cyt - konSrcpR2*VEGF:pR2cyt*Src - konSrcpR2*VEGF:pR2cyt*Hsp90:Src -
konSrcpR2*VEGF:pR2cyt*Akt:Src - konSrcpR2*VEGF:pR2cyt*Akt:Hsp90:Src + koffSrcpR2*VEGF:pR2cyt:Src +
koffSrcpR2*VEGF:pR2cyt:Src:Hsp90 + koffSrcpR2*VEGF:pR2cyt:Src:Akt + koffSrcpR2*VEGF:pR2cyt:Src:Akt:Hsp90 +
kpSrc*VEGF:pR2cyt:Src + kpSrc*VEGF:pR2cyt:Src:Hsp90 + kpSrc*VEGF:pR2cyt:Src:Akt +
kpSrc*VEGF:pR2cyt:Src:Akt:Hsp90 ;

d(VEGF:R2.CD47cyt)/dt = kinterR2CD47*VEGF:R2:CD47mem - kpR2*VEGF:R2.CD47cyt - kdegCD47*VEGF:R2.CD47cyt +
kdpR2CD47bd*VEGF:pR2:CD47cyt ;

d(pPLCg)/dt = (kpPLCg/(KM_PLCgR2+PLCg))*PLCg*VEGF:pR2mem + (kpPLCg/(KM_PLCgR2+PLCg))*PLCg*VEGF:pR2:CD47mem
+ (kpPLCg/(KM_PLCgR2+PLCg))*PLCg*VEGF:pR2cyt - kdpPLCg*pPLCg +
(kpPLCg/(KM_PLCgR2+PLCg))*PLCg*VEGF:pR2:CD47cyt + (kpPLCg/(KM_PLCgR2+PLCg))*PLCg*VEGF:pR2cyt:Src +
(kpPLCg/(KM_PLCgR2+PLCg))*PLCg*VEGF:pR2cyt:Src:Hsp90 +
(kpPLCg/(KM_PLCgR2+PLCg))*PLCg*VEGF:pR2cyt:Src:Akt +
(kpPLCg/(KM_PLCgR2+PLCg))*PLCg*VEGF:pR2cyt:Src:Akt:Hsp90 +
(kpPLCg/(KM_PLCgR2+PLCg))*PLCg*VEGF:pR2cyt:CD47:Src +
(kpPLCg/(KM_PLCgR2+PLCg))*PLCg*VEGF:pR2cyt:CD47:Src:Hsp90 +
(kpPLCg/(KM_PLCgR2+PLCg))*PLCg*VEGF:pR2cyt:CD47:Src:Akt +
(kpPLCg/(KM_PLCgR2+PLCg))*PLCg*VEGF:pR2cyt:CD47:Src:Akt:Hsp90 ;

d(CN:CaM:eNOS)/dt = konCa2C*Ca*Ca*C:CaM:eNOS + konCa2N*Ca*Ca*C:CaM:eNOS + kon4eNOS*CN:CaM*eNOS -
koffCa2C*CN:CaM:eNOS - koffCa2N*CN:CaM:eNOS - koffCaMeNOS*CN:CaM:eNOS -
koneNOSArg*Arg*CN:CaM:eNOS + koffeNOSArg*Arg:CN:CaM:eNOS + kcateNOS*Arg:CN:CaM:eNOS -
konHsp90_CaMeNOS*CN:CaM:eNOS*pHsp90 - konAkteNOS*CN:CaM:eNOS*pAkt +
koffHsp90_CaMeNOS*CN:CaM:eNOS:pHsp90 + koffAkteNOS*CN:CaM:eNOS:pAkt -
konHsp90_CaMeNOS*CN:CaM:eNOS*pAkt:pHsp90 - konHsp90_CaMeNOS*CN:CaM:eNOS*pAkt:Hsp90 -
konHsp90_CaMeNOS*CN:CaM:eNOS*Akt:pHsp90 + koffHsp90_CaMeNOS*CN:CaM:eNOS:pHsp90:pAkt +
kdpeNOS*CN:CaM:peNOS - konHsp90_CaMeNOS*CN:CaM:eNOS*Akt:Hsp90 +
koffHsp90_CaMeNOS*CN:CaM:eNOS:pHsp90:Akt + koffHsp90_CaMeNOS*CN:CaM:eNOS:Hsp90:pAkt +
koffHsp90_CaMeNOS*CN:CaM:eNOS:Hsp90:Akt ;

d(Arg:CN:CaM:eNOS)/dt = konCa2C*Ca*Ca*Arg:N:CaM:eNOS + konCa2N*Ca*Ca*Arg:C:CaM:eNOS +
kon4eNOS*CN:CaM*Arg:eNOS - koffCa2C*Arg:CN:CaM:eNOS - koffCa2N*Arg:CN:CaM:eNOS -
koffCaMeNOS*Arg:CN:CaM:eNOS + koneNOSArg*Arg*CN:CaM:eNOS - koffeNOSArg*Arg:CN:CaM:eNOS -
kcateNOS*Arg:CN:CaM:eNOS - konHsp90_CaMeNOS*Arg:CN:CaM:eNOS*pHsp90 -
konAkteNOS*Arg:CN:CaM:eNOS*pAkt + koffHsp90_CaMeNOS*Arg:CN:CaM:eNOS:pHsp90 +
koffAkteNOS*Arg:CN:CaM:eNOS:pAkt - konHsp90_CaMeNOS*Arg:CN:CaM:eNOS*pAkt:pHsp90 -
konHsp90_CaMeNOS*Arg:CN:CaM:eNOS*pAkt:Hsp90 - konHsp90_CaMeNOS*Arg:CN:CaM:eNOS*Akt:pHsp90
+koffHsp90_CaMeNOS*Arg:CN:CaM:eNOS:pHsp90:pAkt + kdpeNOS*Arg:CN:CaM:peNOS -
konHsp90_CaMeNOS*Arg:CN:CaM:eNOS*Akt:Hsp90 + koffHsp90_CaMeNOS*Arg:CN:CaM:eNOS:pHsp90:Akt +
koffHsp90_CaMeNOS*Arg:CN:CaM:eNOS:Hsp90:pAkt + koffHsp90_CaMeNOS*Arg:CN:CaM:eNOS:Hsp90:Akt ;

d(NO)/dt = kcateNOS*Arg:C:CaM:eNOS + kcateNOS*Arg:N:CaM:eNOS + kcateNOS*Arg:CN:CaM:eNOS -
konNOsGC*sGC_b*NO - kf_GC_NO*sGC*NO - kclearNO*NO + koffNOsGC*sGC_6c - kr_NOGC_NO*sGC:NO*NO +
kr_GC_NO*sGC:NO - konNO_NOGC*sGC_5c*NO + koffNO_NOGC*sGC_5c:NO + kf_NOGC_NO*sGC_5c:NO +

kcateNOS*Arg:C:CaM:eNOS:pAkt + kcateNOS*Arg:N:CaM:eNOS:pAkt + kcateNOS*Arg:CN:CaM:eNOS:pAkt + kcateNOS_H*Arg:C:CaM:eNOS:pHsp90 + kcateNOS_H*Arg:N:CaM:eNOS:pHsp90 + kcateNOS_H*Arg:CN:CaM:eNOS:pHsp90 + kcatpeNOS*Arg:C:CaM:peNOS + kcatpeNOS*Arg:N:CaM:peNOS + kcatpeNOS*Arg:CN:CaM:peNOS + kcateNOS_H*Arg:C:CaM:eNOS:pHsp90:pAkt + kcateNOS_H*Arg:N:CaM:eNOS:pHsp90:pAkt + kcateNOS_H*Arg:CN:CaM:eNOS:pHsp90:pAkt + kcatpeNOS*Arg:peNOS + kcateNOS_H*Arg:C:CaM:eNOS:pHsp90:Akt + kcateNOS_H*Arg:N:CaM:eNOS:pHsp90:Akt + kcateNOS_H*Arg:CN:CaM:eNOS:pHsp90:Akt + kcateNOS_H*Arg:C:CaM:eNOS:Hsp90:pAkt + kcateNOS_H*Arg:N:CaM:eNOS:Hsp90:pAkt + kcateNOS_H*Arg:CN:CaM:eNOS:Hsp90:pAkt + kcatpeNOS_H*Arg:C:CaM:peNOS:pHsp90 + kcatpeNOS_H*Arg:N:CaM:peNOS:pHsp90 + kcatpeNOS_H*Arg:CN:CaM:peNOS:pHsp90 + kcateNOS*Arg:C:CaM:eNOS:Akt + kcateNOS*Arg:N:CaM:eNOS:Akt + kcateNOS*Arg:CN:CaM:eNOS:Hsp90:Akt + kcateNOS_H*Arg:C:CaM:eNOS:Hsp90:Akt + kcateNOS_H*Arg:N:CaM:eNOS:Hsp90:Akt + kcateNOS_H*Arg:CN:CaM:eNOS:Hsp90:Akt + kcateNOS_H*Arg:C:CaM:eNOS:Hsp90 + kcateNOS_H*Arg:N:CaM:eNOS:Hsp90 + kcateNOS_H*Arg:CN:CaM:eNOS:Hsp90 + kcatpeNOS_H*Arg:peNOS:pHsp90 ;

d(Cit)/dt = kcateNOS*Arg:C:CaM:eNOS + kcateNOS*Arg:N:CaM:eNOS + kcateNOS*Arg:CN:CaM:eNOS + kcateNOS*Arg:C:CaM:eNOS:pAkt + kcateNOS*Arg:N:CaM:eNOS:pAkt + kcateNOS*Arg:CN:CaM:eNOS:pAkt + kcateNOS_H*Arg:C:CaM:eNOS:pHsp90 + kcateNOS_H*Arg:N:CaM:eNOS:pHsp90 + kcateNOS_H*Arg:CN:CaM:eNOS:pHsp90 + kcatpeNOS*Arg:C:CaM:peNOS + kcatpeNOS*Arg:N:CaM:peNOS + kcatpeNOS*Arg:CN:CaM:peNOS + kcateNOS_H*Arg:C:CaM:eNOS:pHsp90:pAkt + kcateNOS_H*Arg:N:CaM:eNOS:pHsp90:pAkt + kcateNOS_H*Arg:CN:CaM:eNOS:pHsp90:pAkt + kcatpeNOS*Arg:peNOS + kcateNOS_H*Arg:C:CaM:eNOS:pHsp90:Akt + kcateNOS_H*Arg:N:CaM:eNOS:pHsp90:Akt + kcateNOS_H*Arg:CN:CaM:eNOS:pHsp90:Akt + kcateNOS_H*Arg:C:CaM:eNOS:Hsp90:pAkt + kcateNOS_H*Arg:N:CaM:eNOS:Hsp90:pAkt + kcateNOS_H*Arg:CN:CaM:eNOS:Hsp90:pAkt + kcatpeNOS_H*Arg:C:CaM:peNOS:pHsp90 + kcatpeNOS_H*Arg:N:CaM:peNOS:pHsp90 + kcatpeNOS_H*Arg:CN:CaM:peNOS:pHsp90 + kcateNOS*Arg:C:CaM:eNOS:Akt + kcateNOS*Arg:N:CaM:eNOS:Akt + kcateNOS*Arg:CN:CaM:eNOS:Hsp90:Akt + kcateNOS_H*Arg:C:CaM:eNOS:Hsp90:Akt + kcateNOS_H*Arg:N:CaM:eNOS:Hsp90:Akt + kcateNOS_H*Arg:CN:CaM:eNOS:Hsp90:Akt + kcateNOS_H*Arg:C:CaM:eNOS:Hsp90 + kcateNOS_H*Arg:N:CaM:eNOS:Hsp90 + kcateNOS_H*Arg:CN:CaM:eNOS:Hsp90 + kcatpeNOS_H*Arg:peNOS:pHsp90 ;

d(VEGF:pR2:CD47cyt)/dt = kinterpR2CD47*VEGF:pR2:CD47mem + kpR2*VEGF:R2.CD47cyt - kdpR2CD47bd*VEGF:pR2:CD47cyt - kdegpR2CD47*VEGF:pR2:CD47cyt - konSrcpR2*VEGF:pR2:CD47cyt*Src - konSrcpR2*VEGF:pR2:CD47cyt*Hsp90:Src - konSrcpR2*VEGF:pR2:CD47cyt*Akt:Src - konSrcpR2*VEGF:pR2:CD47cyt*Akt:Hsp90:Src + koffSrcpR2*VEGF:pR2cyt:CD47:Src + koffSrcpR2*VEGF:pR2cyt:CD47:Src:Hsp90 + koffSrcpR2*VEGF:pR2cyt:CD47:Src:Akt + koffSrcpR2*VEGF:pR2cyt:CD47:Src:Akt:Hsp90 + kpSrc*VEGF:pR2cyt:CD47:Src + kpSrc*VEGF:pR2cyt:CD47:Src:Hsp90 + kpSrc*VEGF:pR2cyt:CD47:Src:Akt + kpSrc*VEGF:pR2cyt:CD47:Src:Akt:Hsp90 ;

d(VEGF:pR2cyt:Src)/dt = konSrcpR2*VEGF:pR2cyt*Src - koffSrcpR2*VEGF:pR2cyt:Src - kpSrc*VEGF:pR2cyt:Src ;

d(VEGF:pR2cyt:Src:Hsp90)/dt = konSrcpR2*VEGF:pR2cyt*Hsp90:Src - koffSrcpR2*VEGF:pR2cyt:Src:Hsp90 - kpSrc*VEGF:pR2cyt:Src:Hsp90 ;

d(VEGF:pR2cyt:Src:Akt)/dt = konSrcpR2*VEGF:pR2cyt*Akt:Src - koffSrcpR2*VEGF:pR2cyt:Src:Akt - kpSrc*VEGF:pR2cyt:Src:Akt ;

d(VEGF:pR2cyt:Src:Akt:Hsp90)/dt = konSrcpR2*VEGF:pR2cyt*Akt:Hsp90:Src - koffSrcpR2*VEGF:pR2cyt:Src:Akt:Hsp90 - kpSrc*VEGF:pR2cyt:Src:Akt:Hsp90 ;

d(IP3)/dt = RF2(parameters,observables)*pPLCg*PIP2 - kdegIP3*IP3 ;

d(sGC_6c)/dt = konNOsGC*sGC_b*NO - koffNOsGC*sGC_6c - ka_sGC*sGC_6c + kd_sGC*sGC_5c ;

d(sGC:NO)/dt = kf_GC_NO*sGC*NO - kr_NOGC_NO*sGC:NO*NO - kr_GC_NO*sGC:NO + kf_NOGC_NO*sGC_5c:NO ;

d(VEGF:pR2cyt:CD47:Src)/dt = konSrcpR2*VEGF:pR2:CD47cyt*Src - koffSrcpR2*VEGF:pR2cyt:CD47:Src - kpSrc*VEGF:pR2cyt:CD47:Src ;

d(VEGF:pR2cyt:CD47:Src:Hsp90)/dt = konSrcpR2*VEGF:pR2:CD47cyt*Hsp90:Src - koffSrcpR2*VEGF:pR2cyt:CD47:Src:Hsp90 - kpSrc*VEGF:pR2cyt:CD47:Src:Hsp90 ;

d(VEGF:pR2cyt:CD47:Src:Akt)/dt = konSrcpR2*VEGF:pR2:CD47cyt*Akt:Src - koffSrcpR2*VEGF:pR2cyt:CD47:Src:Akt - kpSrc*VEGF:pR2cyt:CD47:Src:Akt ;

d(VEGF:pR2cyt:CD47:Src:Akt:Hsp90)/dt = konSrcpR2*VEGF:pR2:CD47cyt*Akt:Hsp90:Src - koffSrcpR2*VEGF:pR2cyt:CD47:Src:Akt:Hsp90 - kpSrc*VEGF:pR2cyt:CD47:Src:Akt:Hsp90 ;

d(pSrc)/dt = kpSrc*VEGF:pR2cyt:Src + kpSrc*VEGF:pR2cyt:CD47:Src - kdpSrc*pSrc - konSrcHsp90*pSrc*Hsp90 + koffSrcHsp90*pSrc:Hsp90 + kpHsp90*pSrc:Hsp90 - konSrcAkt*pSrc*Akt + koffSrcAkt*pSrc:Akt + kpAkt*pSrc:Akt ;

d(pSrc:Hsp90)/dt = kpSrc*VEGF:pR2cyt:Src:Hsp90 + kpSrc*VEGF:pR2cyt:CD47:Src:Hsp90 - kdpSrc*pSrc:Hsp90 + konSrcHsp90*pSrc*Hsp90 - koffSrcHsp90*pSrc:Hsp90 - kpHsp90*pSrc:Hsp90 ;

d(pSrc:Akt)/dt = kpSrc*VEGF:pR2cyt:Src:Akt + kpSrc*VEGF:pR2cyt:CD47:Src:Akt - kdpSrc*pSrc:Akt - konSrcHsp90*pSrc:Akt*Hsp90 + koffSrcHsp90*pSrc:Hsp90:Akt + kpHsp90*pSrc:Hsp90:Akt + konSrcAkt*pSrc*Akt - koffSrcAkt*pSrc:Akt - kpAkt*pSrc:Akt ;

d(pSrc:Hsp90:Akt)/dt = kpSrc*VEGF:pR2cyt:Src:Akt:Hsp90 + kpSrc*VEGF:pR2cyt:CD47:Src:Akt:Hsp90 - kdpSrc*pSrc:Hsp90:Akt + konSrcHsp90*pSrc:Akt*Hsp90 - koffSrcHsp90*pSrc:Hsp90:Akt - kpHsp90*pSrc:Hsp90:Akt ;

d(sGC_5c)/dt = ka_sGC*sGC_6c - kd_sGC*sGC_5c - konNO_NOGC*sGC_5c*NO + koffNO_NOGC*sGC_5c:NO ;

d(sGC_5c:NO)/dt = kr_NOGC_NO*sGC:NO*NO + konNO_NOGC*sGC_5c*NO - koffNO_NOGC*sGC_5c:NO - kf_NOGC_NO*sGC_5c:NO ;

d(pHsp90)/dt = kpHsp90*pSrc:Hsp90 + kpHsp90*pSrc:Hsp90:Akt - kdpHsp90*pHsp90 - konHsp90eNOS*eNOS*pHsp90 - konHsp90eNOS*Arg:eNOS*pHsp90 - konHsp90_CaMeNOS*C:CaM:eNOS*pHsp90 - konHsp90_CaMeNOS*Arg:C:CaM:eNOS*pHsp90 - konHsp90_CaMeNOS*N:CaM:eNOS*pHsp90 - konHsp90_CaMeNOS*Arg:N:CaM:eNOS*pHsp90 - konHsp90_CaMeNOS*CN:CaM:eNOS*pHsp90 - konHsp90_CaMeNOS*Arg:CN:CaM:eNOS*pHsp90 - konpAktHsp90*pAkt*pHsp90 + koffHsp90eNOS*eNOS:pHsp90 + koffHsp90eNOS*Arg:eNOS:pHsp90 + koffHsp90_CaMeNOS*C:CaM:eNOS:pHsp90 + koffHsp90_CaMeNOS*Arg:C:CaM:eNOS:pHsp90 + koffHsp90_CaMeNOS*N:CaM:eNOS:pHsp90 + koffHsp90_CaMeNOS*Arg:N:CaM:eNOS:pHsp90 + koffHsp90_CaMeNOS*CN:CaM:eNOS:pHsp90 + koffHsp90_CaMeNOS*Arg:CN:CaM:eNOS:pHsp90 + koffpAktHsp90*pAkt:pHsp90 - konHsp90eNOS*eNOS:pAkt*pHsp90 - konHsp90eNOS*Arg:eNOS:pAkt*pHsp90 - konHsp90_CaMeNOS*C:CaM:eNOS:pAkt*pHsp90 - konHsp90_CaMeNOS*Arg:C:CaM:eNOS:pAkt*pHsp90 - konHsp90_CaMeNOS*N:CaM:eNOS:pAkt*pHsp90 - konHsp90_CaMeNOS*Arg:N:CaM:eNOS:pAkt*pHsp90 - konHsp90_CaMeNOS*CN:CaM:eNOS:pAkt*pHsp90 - konHsp90_CaMeNOS*Arg:CN:CaM:eNOS:pAkt*pHsp90 - konHsp90_CaMeNOS*C:CaM:peNOS*pHsp90 - konHsp90_CaMeNOS*Arg:C:CaM:peNOS*pHsp90 - konHsp90_CaMeNOS*N:CaM:peNOS*pHsp90 - konHsp90_CaMeNOS*Arg:N:CaM:peNOS*pHsp90 - konHsp90_CaMeNOS*CN:CaM:peNOS*pHsp90 - konHsp90_CaMeNOS*Arg:CN:CaM:peNOS*pHsp90 + koffHsp90eNOS*eNOS:pHsp90:pAkt + koffHsp90eNOS*Arg:eNOS:pHsp90:pAkt + koffHsp90_CaMeNOS*C:CaM:eNOS:pHsp90:pAkt + koffHsp90_CaMeNOS*Arg:C:CaM:eNOS:pHsp90:pAkt + koffHsp90_CaMeNOS*N:CaM:eNOS:pHsp90:pAkt + koffHsp90_CaMeNOS*Arg:N:CaM:eNOS:pHsp90:pAkt + koffHsp90_CaMeNOS*CN:CaM:eNOS:pHsp90:pAkt + koffHsp90_CaMeNOS*Arg:CN:CaM:eNOS:pHsp90:pAkt - konHsp90eNOS*peNOS*pHsp90 - konHsp90eNOS*Arg:peNOS*pHsp90 + koffHsp90_CaMeNOS*C:CaM:peNOS:pHsp90 + koffHsp90_CaMeNOS*Arg:C:CaM:peNOS:pHsp90 + koffHsp90_CaMeNOS*N:CaM:peNOS:pHsp90 + koffHsp90_CaMeNOS*N:CaM:peNOS:pHsp90 + koffHsp90_CaMeNOS*CN:CaM:peNOS:pHsp90 + koffHsp90_CaMeNOS*Arg:CN:CaM:peNOS:pHsp90 + koffHsp90eNOS*eNOS:pHsp90:Akt + koffHsp90eNOS*Arg:eNOS:pHsp90:Akt + koffHsp90_CaMeNOS*C:CaM:eNOS:pHsp90:Akt + koffHsp90_CaMeNOS*Arg:C:CaM:eNOS:pHsp90:Akt + koffHsp90_CaMeNOS*N:CaM:eNOS:pHsp90:Akt + koffHsp90_CaMeNOS*Arg:N:CaM:eNOS:pHsp90:Akt + koffHsp90_CaMeNOS*CN:CaM:eNOS:pHsp90:Akt + koffHsp90_CaMeNOS*Arg:CN:CaM:eNOS:pHsp90:Akt + koffHsp90eNOS*peNOS:pHsp90 + koffHsp90eNOS*Arg:peNOS:pHsp90 - konHsp90eNOS*Akt:eNOS*pHsp90 - konHsp90eNOS*Arg:Akt:eNOS*pHsp90 - konHsp90_CaMeNOS*C:CaM:eNOS:Akt*pHsp90 - konHsp90_CaMeNOS*Arg:C:CaM:eNOS:Akt*pHsp90 - konHsp90_CaMeNOS*N:CaM:eNOS:Akt*pHsp90 - konHsp90_CaMeNOS*Arg:N:CaM:eNOS:Akt*pHsp90 - konHsp90_CaMeNOS*CN:CaM:eNOS:Hsp90:Akt*pHsp90 - konHsp90_CaMeNOS*Arg:CN:CaM:eNOS:Hsp90:Akt*pHsp90 ;

d(pAkt)/dt = kpAkt*pSrc:Akt - kdpAkt*pAkt - konAkteNOS*eNOS*pAkt - konAkteNOS*Arg:eNOS*pAkt - konAkteNOS*C:CaM:eNOS*pAkt - konAkteNOS*Arg:C:CaM:eNOS*pAkt - konAkteNOS*N:CaM:eNOS*pAkt - konAkteNOS*Arg:N:CaM:eNOS*pAkt - konAkteNOS*CN:CaM:eNOS*pAkt - konAkteNOS*Arg:CN:CaM:eNOS*pAkt - konpAktHsp90*pAkt*pHsp90 + koffAkteNOS*eNOS:pAkt + koffAkteNOS*Arg:eNOS:pAkt + koffAkteNOS*C:CaM:eNOS:pAkt + koffAkteNOS*Arg:C:CaM:eNOS:pAkt + koffAkteNOS*N:CaM:eNOS:pAkt + koffAkteNOS*Arg:N:CaM:eNOS:pAkt + koffAkteNOS*CN:CaM:eNOS:pAkt + koffAkteNOS*Arg:CN:CaM:eNOS:pAkt + koffpAktHsp90*pAkt:pHsp90 - konpAktHsp90*eNOS:pHsp90*pAkt - konpAktHsp90*Arg:eNOS:pHsp90*pAkt -

konpAktHsp90*C:CaM:eNOS:pHsp90*pAkt - konpAktHsp90*Arg:C:CaM:eNOS:pHsp90*pAkt -
konpAktHsp90*N:CaM:eNOS:pHsp90*pAkt - konpAktHsp90*Arg:N:CaM:eNOS:pHsp90*pAkt -
konpAktHsp90*CN:CaM:eNOS:pHsp90*pAkt - konpAktHsp90*Arg:CN:CaM:eNOS:pHsp90*pAkt +
kcatpAkt*C:CaM:eNOS:pAkt + kcatpAkt*Arg:C:CaM:eNOS:pAkt + kcatpAkt*N:CaM:eNOS:pAkt +
kcatpAkt*Arg:N:CaM:eNOS:pAkt + kcatpAkt*CN:CaM:eNOS:pAkt + kcatpAkt*Arg:CN:CaM:eNOS:pAkt +
koffpAktHsp90*eNOS:pHsp90:pAkt + koffpAktHsp90*Arg:eNOS:pHsp90:pAkt +
koffpAktHsp90*C:CaM:eNOS:pHsp90:pAkt + koffpAktHsp90*Arg:C:CaM:eNOS:pHsp90:pAkt +
koffpAktHsp90*N:CaM:eNOS:pHsp90:pAkt + koffpAktHsp90*Arg:N:CaM:eNOS:pHsp90:pAkt +
koffpAktHsp90*CN:CaM:eNOS:pHsp90:pAkt + koffpAktHsp90*Arg:CN:CaM:eNOS:pHsp90:pAkt +
kcatpAkt_H*C:CaM:eNOS:pHsp90:pAkt + kcatpAkt_H*Arg:C:CaM:eNOS:pHsp90:pAkt +
kcatpAkt_H*N:CaM:eNOS:pHsp90:pAkt + kcatpAkt_H*Arg:N:CaM:eNOS:pHsp90:pAkt +
kcatpAkt_H*CN:CaM:eNOS:pHsp90:pAkt + kcatpAkt_H*Arg:CN:CaM:eNOS:pHsp90:pAkt +
koffpAktHsp90*eNOS:Hsp90:pAkt + koffpAktHsp90*Arg:eNOS:Hsp90:pAkt +
koffpAktHsp90*C:CaM:eNOS:Hsp90:pAkt + koffpAktHsp90*Arg:C:CaM:eNOS:Hsp90:pAkt +
koffpAktHsp90*N:CaM:eNOS:Hsp90:pAkt + koffpAktHsp90*Arg:N:CaM:eNOS:Hsp90:pAkt +
koffpAktHsp90*CN:CaM:eNOS:Hsp90:pAkt + koffpAktHsp90*Arg:CN:CaM:eNOS:Hsp90:pAkt -
konpAktHsp90*eNOS:Hsp90*pAkt - konpAktHsp90*Arg:eNOS:Hsp90*pAkt -
konpAktHsp90*C:CaM:eNOS:Hsp90*pAkt - konpAktHsp90*Arg:C:CaM:eNOS:Hsp90*pAkt -
konpAktHsp90*N:CaM:eNOS:Hsp90*pAkt - konpAktHsp90*Arg:N:CaM:eNOS:Hsp90*pAkt -
konpAktHsp90*CN:CaM:eNOS:Hsp90*pAkt - konpAktHsp90*Arg:CN:CaM:eNOS:Hsp90*pAkt ;

d(cGMP)/dt = kcatsGC*GTP*sGC_5c - koncGMPPDE*cGMP*PDE -
((((kcatPDE*0.05)*(PDE+cGMP:PDE))/(KM_PDE+cGMP))+((kcatPDE*cGMP:PDE_a)/(KM_PDE+cGMP)))*cGMP +
koffcGMPPDE*cGMP:PDE ;

d(eNOS:pHsp90)/dt = konHsp90eNOS*eNOS*pHsp90 - kon2CeNOS_H*C:CaM*eNOS:pHsp90 +
koffCaMeNOS_H*C:CaM:eNOS:pHsp90 - kon2NeNOS_H*N:CaM*eNOS:pHsp90 +
koffCaMeNOS_H*N:CaM:eNOS:pHsp90 - kon4eNOS_H*CN:CaM*eNOS:pHsp90 +
koffCaMeNOS_H*CN:CaM:eNOS:pHsp90 - koffHsp90eNOS*eNOS:pHsp90 - konpAktHsp90*eNOS:pHsp90*pAkt -
konbeNOSArg*Arg*eNOS:pHsp90 + koffbeNOSArg*Arg:eNOS:pHsp90 + koffpAktHsp90*eNOS:pHsp90:pAkt +
kdpeNOS*peNOS:pHsp90 ;

d(Arg:eNOS:pHsp90)/dt = konHsp90eNOS*Arg:eNOS*pHsp90 - kon2CeNOS_H*C:CaM*Arg:eNOS:pHsp90 +
koffCaMeNOS_H*Arg:C:CaM:eNOS:pHsp90 - kon2NeNOS_H*N:CaM*Arg:eNOS:pHsp90 +
koffCaMeNOS_H*Arg:N:CaM:eNOS:pHsp90 - kon4eNOS_H*CN:CaM*Arg:eNOS:pHsp90 +
koffCaMeNOS_H*Arg:CN:CaM:eNOS:pHsp90 - koffHsp90eNOS*Arg:eNOS:pHsp90 -
konpAktHsp90*Arg:eNOS:pHsp90*pAkt + konbeNOSArg*Arg*eNOS:pHsp90 - koffbeNOSArg*Arg:eNOS:pHsp90 +
koffpAktHsp90*Arg:eNOS:pHsp90:pAkt + kdpeNOS*Arg:peNOS:pHsp90 ;

d(C:CaM:eNOS:pHsp90)/dt = konHsp90_CaMeNOS*C:CaM:eNOS*pHsp90 - konCa2N*Ca*Ca*C:CaM:eNOS:pHsp90 +
koffCa2N*CN:CaM:eNOS:pHsp90 + kon2CeNOS_H*C:CaM*eNOS:pHsp90 - koffCaMeNOS_H*C:CaM:eNOS:pHsp90 -
koffHsp90_CaMeNOS*C:CaM:eNOS:pHsp90 - konpAktHsp90*C:CaM:eNOS:pHsp90*pAkt -
koneNOSArg_H*Arg*C:CaM:eNOS:pHsp90 + koffeNOSArg_H*Arg:C:CaM:eNOS:pHsp90 +
kcateNOS_H*Arg:C:CaM:eNOS:pHsp90 + koffpAktHsp90*C:CaM:eNOS:pHsp90:pAkt +
kdpeNOS*C:CaM:peNOS:pHsp90 ;

d(Arg:C:CaM:eNOS:pHsp90)/dt = konHsp90_CaMeNOS*Arg:C:CaM:eNOS*pHsp90 - konCa2N*Ca*Ca*Arg:C:CaM:eNOS:pHsp90
+ koffCa2N*Arg:CN:CaM:eNOS:pHsp90 + kon2CeNOS_H*C:CaM*Arg:eNOS:pHsp90 -
koffCaMeNOS_H*Arg:C:CaM:eNOS:pHsp90 - koffHsp90_CaMeNOS*Arg:C:CaM:eNOS:pHsp90 -
konpAktHsp90*Arg:C:CaM:eNOS:pHsp90*pAkt + koneNOSArg_H*Arg*C:CaM:eNOS:pHsp90 -
koffeNOSArg_H*Arg:C:CaM:eNOS:pHsp90 - kcateNOS_H*Arg:C:CaM:eNOS:pHsp90 +
koffpAktHsp90*Arg:C:CaM:eNOS:pHsp90:pAkt + kdpeNOS*Arg:C:CaM:peNOS:pHsp90 ;

d(N:CaM:eNOS:pHsp90)/dt = konHsp90_CaMeNOS*N:CaM:eNOS*pHsp90 - konCa2C*Ca*Ca*N:CaM:eNOS:pHsp90 +
koffCa2C*CN:CaM:eNOS:pHsp90 + kon2NeNOS_H*N:CaM*eNOS:pHsp90 - koffCaMeNOS_H*N:CaM:eNOS:pHsp90 -
koffHsp90_CaMeNOS*N:CaM:eNOS:pHsp90 - konpAktHsp90*N:CaM:eNOS:pHsp90*pAkt -
koneNOSArg_H*Arg*N:CaM:eNOS:pHsp90 + koffeNOSArg_H*Arg:N:CaM:eNOS:pHsp90 +
kcateNOS_H*Arg:N:CaM:eNOS:pHsp90 + koffpAktHsp90*N:CaM:eNOS:pHsp90:pAkt +
kdpeNOS*N:CaM:peNOS:pHsp90 ;

d(Arg:N:CaM:eNOS:pHsp90)/dt = konHsp90_CaMeNOS*Arg:N:CaM:eNOS*pHsp90 -
konCa2C*Ca*Ca*Arg:N:CaM:eNOS:pHsp90 + koffCa2C*Arg:CN:CaM:eNOS:pHsp90 +

kon2NeNOS_H*N:CaM*Arg:eNOS:pHsp90 - koffCaMeNOS_H*Arg:N:CaM:eNOS:pHsp90 - koffHsp90_CaMeNOS*Arg:N:CaM:eNOS:pHsp90 - konpAktHsp90*Arg:N:CaM:eNOS:pHsp90*pAkt + koneNOSArg_H*Arg*N:CaM:eNOS:pHsp90 - koffeNOSArg_H*Arg:N:CaM:eNOS:pHsp90 - kcateNOS_H*Arg:N:CaM:eNOS:pHsp90 + koffpAktHsp90*Arg:N:CaM:eNOS:pHsp90:pAkt + kdpeNOS*Arg:N:CaM:peNOS:pHsp90 ;

d(CN:CaM:eNOS:pHsp90)/dt = konHsp90_CaMeNOS*CN:CaM:eNOS*pHsp90 + konCa2C*Ca*Ca*N:CaM:eNOS:pHsp90 - koffCa2C*CN:CaM:eNOS:pHsp90 + konCa2N*Ca*Ca*C:CaM:eNOS:pHsp90 - koffCa2N*CN:CaM:eNOS:pHsp90 + kon4eNOS_H*CN:CaM*eNOS:pHsp90 - koffCaMeNOS_H*CN:CaM:eNOS:pHsp90 - koffHsp90_CaMeNOS*CN:CaM:eNOS:pHsp90 - konpAktHsp90*CN:CaM:eNOS:pHsp90*pAkt - koneNOSArg_H*Arg*CN:CaM:eNOS:pHsp90 + koffeNOSArg_H*Arg:CN:CaM:eNOS:pHsp90 + kcateNOS_H*Arg:CN:CaM:eNOS:pHsp90 + koffpAktHsp90*CN:CaM:eNOS:pHsp90:pAkt + kdpeNOS*CN:CaM:peNOS:pHsp90 ;

d(Arg:CN:CaM:eNOS:pHsp90)/dt = konHsp90_CaMeNOS*Arg:CN:CaM:eNOS*pHsp90 + konCa2C*Ca*Ca*Arg:N:CaM:eNOS:pHsp90 - koffCa2C*Arg:CN:CaM:eNOS:pHsp90 + konCa2N*Ca*Ca*Arg:C:CaM:eNOS:pHsp90 - koffCa2N*Arg:CN:CaM:eNOS:pHsp90 + kon4eNOS_H*CN:CaM*Arg:eNOS:pHsp90 - koffCaMeNOS_H*Arg:CN:CaM:eNOS:pHsp90 - koffHsp90_CaMeNOS*Arg:CN:CaM:eNOS:pHsp90 - konpAktHsp90*Arg:CN:CaM:eNOS:pHsp90*pAkt + koneNOSArg_H*Arg*CN:CaM:eNOS:pHsp90 - koffeNOSArg_H*Arg:CN:CaM:eNOS:pHsp90 - kcateNOS_H*Arg:CN:CaM:eNOS:pHsp90 + koffpAktHsp90*Arg:CN:CaM:eNOS:pHsp90:pAkt + kdpeNOS*Arg:CN:CaM:peNOS:pHsp90 ;

d(eNOS:pAkt)/dt = konAkteNOS*eNOS*pAkt - kon2CpeNOS*C:CaM*eNOS:pAkt + koffCaMpeNOS*C:CaM:eNOS:pAkt - kon2NpeNOS*N:CaM*eNOS:pAkt + koffCaMpeNOS*N:CaM:eNOS:pAkt - kon4peNOS*CN:CaM*eNOS:pAkt + koffCaMpeNOS*CN:CaM:eNOS:pAkt - koffAkteNOS*eNOS:pAkt - konHsp90eNOS*eNOS:pAkt*pHsp90 - konbeNOSArg*Arg*eNOS:pAkt + koffbeNOSArg*Arg:eNOS:pAkt + koffHsp90eNOS*eNOS:pHsp90:pAkt ;

d(Arg:eNOS:pAkt)/dt = konAkteNOS*Arg:eNOS*pAkt - kon2CpeNOS*C:CaM*Arg:eNOS:pAkt + koffCaMpeNOS*Arg:C:CaM:eNOS:pAkt - kon2NpeNOS*N:CaM*Arg:eNOS:pAkt + koffCaMpeNOS*Arg:N:CaM:eNOS:pAkt - kon4peNOS*CN:CaM*Arg:eNOS:pAkt + koffCaMpeNOS*Arg:CN:CaM:eNOS:pAkt - koffAkteNOS*Arg:eNOS:pAkt - konHsp90eNOS*Arg:eNOS:pAkt*pHsp90 + konbeNOSArg*Arg*eNOS:pAkt - koffbeNOSArg*Arg:eNOS:pAkt + koffHsp90eNOS*Arg:eNOS:pHsp90:pAkt ;

d(C:CaM:eNOS:pAkt)/dt = konAkteNOS*C:CaM:eNOS*pAkt - konCa2N*Ca*Ca*C:CaM:eNOS:pAkt + koffCa2N*CN:CaM:eNOS:pAkt + kon2CpeNOS*C:CaM*eNOS:pAkt - koffCaMpeNOS*C:CaM:eNOS:pAkt - koffAkteNOS*C:CaM:eNOS:pAkt - konHsp90_CaMeNOS*C:CaM:eNOS:pAkt*pHsp90 - kcatpAkt*C:CaM:eNOS:pAkt - koneNOSArg*Arg*C:CaM:eNOS:pAkt + koffeNOSArg*Arg:C:CaM:eNOS:pAkt + kcateNOS*Arg:C:CaM:eNOS:pAkt + koffHsp90_CaMeNOS*C:CaM:eNOS:pHsp90:pAkt ;

d(Arg:C:CaM:eNOS:pAkt)/dt = konAkteNOS*Arg:C:CaM:eNOS*pAkt - konCa2N*Ca*Ca*Arg:C:CaM:eNOS:pAkt + koffCa2N*Arg:CN:CaM:eNOS:pAkt + kon2CpeNOS*C:CaM*Arg:eNOS:pAkt - koffCaMpeNOS*Arg:C:CaM:eNOS:pAkt - koffAkteNOS*Arg:C:CaM:eNOS:pAkt - konHsp90_CaMeNOS*Arg:C:CaM:eNOS:pAkt*pHsp90 - kcatpAkt*Arg:C:CaM:eNOS:pAkt + koneNOSArg*Arg*C:CaM:eNOS:pAkt - koffeNOSArg*Arg:C:CaM:eNOS:pAkt - kcateNOS*Arg:C:CaM:eNOS:pAkt + koffHsp90_CaMeNOS*Arg:C:CaM:eNOS:pHsp90:pAkt ;

d(N:CaM:eNOS:pAkt)/dt = konAkteNOS*N:CaM:eNOS*pAkt - konCa2C*Ca*Ca*N:CaM:eNOS:pAkt + koffCa2C*CN:CaM:eNOS:pAkt + kon2NpeNOS*N:CaM*eNOS:pAkt - koffCaMpeNOS*N:CaM:eNOS:pAkt - koffAkteNOS*N:CaM:eNOS:pAkt - konHsp90_CaMeNOS*N:CaM:eNOS:pAkt*pHsp90 - kcatpAkt*N:CaM:eNOS:pAkt - koneNOSArg*Arg*N:CaM:eNOS:pAkt + koffeNOSArg*Arg:N:CaM:eNOS:pAkt + kcateNOS*Arg:N:CaM:eNOS:pAkt + koffHsp90_CaMeNOS*N:CaM:eNOS:pHsp90:pAkt ;

d(Arg:N:CaM:eNOS:pAkt)/dt = konAkteNOS*Arg:N:CaM:eNOS*pAkt - konCa2C*Ca*Ca*Arg:N:CaM:eNOS:pAkt + koffCa2C*Arg:CN:CaM:eNOS:pAkt + kon2NpeNOS*N:CaM*Arg:eNOS:pAkt - koffCaMpeNOS*Arg:N:CaM:eNOS:pAkt - koffAkteNOS*Arg:N:CaM:eNOS:pAkt - konHsp90_CaMeNOS*Arg:N:CaM:eNOS:pAkt*pHsp90 - kcatpAkt*Arg:N:CaM:eNOS:pAkt + koneNOSArg*Arg*N:CaM:eNOS:pAkt - koffeNOSArg*Arg:N:CaM:eNOS:pAkt - kcateNOS*Arg:N:CaM:eNOS:pAkt + koffHsp90_CaMeNOS*Arg:N:CaM:eNOS:pHsp90:pAkt ;

d(CN:CaM:eNOS:pAkt)/dt = konAkteNOS*CN:CaM:eNOS*pAkt + konCa2C*Ca*Ca*N:CaM:eNOS:pAkt - koffCa2C*CN:CaM:eNOS:pAkt + konCa2N*Ca*Ca*C:CaM:eNOS:pAkt - koffCa2N*CN:CaM:eNOS:pAkt + kon4peNOS*CN:CaM*eNOS:pAkt - koffCaMpeNOS*CN:CaM:eNOS:pAkt - koffAkteNOS*CN:CaM:eNOS:pAkt - konHsp90_CaMeNOS*CN:CaM:eNOS:pAkt*pHsp90 - kcatpAkt*CN:CaM:eNOS:pAkt -

koneNOSArg*Arg*CN:CaM:eNOS:pAkt + koffeNOSArg*Arg:CN:CaM:eNOS:pAkt + kcateNOS*Arg:CN:CaM:eNOS:pAkt + koffHsp90_CaMeNOS*CN:CaM:eNOS:pHsp90:pAkt ;

d(Arg:CN:CaM:eNOS:pAkt)/dt = konAkteNOS*Arg:CN:CaM:eNOS*pAkt + konCa2C*Ca*Ca*Arg:N:CaM:eNOS:pAkt - koffCa2C*Arg:CN:CaM:eNOS:pAkt + konCa2N*Ca*Ca*Arg:C:CaM:eNOS:pAkt - koffCa2N*Arg:CN:CaM:eNOS:pAkt + kon4peNOS*CN:CaM*Arg:eNOS:pAkt - koffCaMpeNOS*Arg:CN:CaM:eNOS:pAkt - koffAkteNOS*Arg:CN:CaM:eNOS:pAkt - konHsp90_CaMeNOS*Arg:CN:CaM:eNOS:pAkt*pHsp90 - kcatpAkt*Arg:CN:CaM:eNOS:pAkt + koneNOSArg*Arg*CN:CaM:eNOS:pAkt - koffeNOSArg*Arg:CN:CaM:eNOS:pAkt - kcateNOS*Arg:CN:CaM:eNOS:pAkt + koffHsp90_CaMeNOS*Arg:CN:CaM:eNOS:pHsp90:pAkt ;

d(pAkt:pHsp90)/dt = konpAktHsp90*pAkt*pHsp90 - kdpHsp90*pAkt:pHsp90 - kdpAkt*pAkt:pHsp90 - koffpAktHsp90*pAkt:pHsp90 - konHsp90eNOS*eNOS*pAkt:pHsp90 - konHsp90eNOS*Arg:eNOS*pAkt:pHsp90 - konHsp90_CaMeNOS*C:CaM:eNOS*pAkt:pHsp90 - konHsp90_CaMeNOS*Arg:C:CaM:eNOS*pAkt:pHsp90 - konHsp90_CaMeNOS*N:CaM:eNOS*pAkt:pHsp90 - konHsp90_CaMeNOS*Arg:N:CaM:eNOS*pAkt:pHsp90 - konHsp90_CaMeNOS*CN:CaM:eNOS*pAkt:pHsp90 - konHsp90_CaMeNOS*Arg:CN:CaM:eNOS*pAkt:pHsp90 + koffHsp90eNOS*eNOS:pHsp90:pAkt + koffHsp90eNOS*Arg:eNOS:pHsp90:pAkt + koffHsp90_CaMeNOS*C:CaM:eNOS:pHsp90:pAkt + koffHsp90_CaMeNOS*Arg:C:CaM:eNOS:pHsp90:pAkt + koffHsp90_CaMeNOS*N:CaM:eNOS:pHsp90:pAkt + koffHsp90_CaMeNOS*Arg:N:CaM:eNOS:pHsp90:pAkt + koffHsp90_CaMeNOS*CN:CaM:eNOS:pHsp90:pAkt +koffHsp90_CaMeNOS*Arg:CN:CaM:eNOS:pHsp90:pAkt ;

d(cGMP:PDE)/dt = koncGMPPDE*cGMP*PDE - koffcGMPPDE*cGMP:PDE - ka_PDE*cGMP:PDE + kd_PDE*cGMP:PDE_a ;

d(GMP)/dt = ((((kcatPDE*0.05)*(PDE+cGMP:PDE))/(KM_PDE+cGMP))+((kcatPDE*cGMP:PDE_a)/(KM_PDE+cGMP)))*cGMP ;

d(pAkt:Hsp90)/dt = kdpHsp90*pAkt:pHsp90 - kdpAkt*pAkt:Hsp90 - konHsp90eNOS*eNOS*pAkt:Hsp90 - konHsp90eNOS*Arg:eNOS*pAkt:Hsp90 - konHsp90_CaMeNOS*C:CaM:eNOS*pAkt:Hsp90 - konHsp90_CaMeNOS*Arg:C:CaM:eNOS*pAkt:Hsp90 - konHsp90_CaMeNOS*N:CaM:eNOS*pAkt:Hsp90 - konHsp90_CaMeNOS*Arg:N:CaM:eNOS*pAkt:Hsp90 - konHsp90_CaMeNOS*CN:CaM:eNOS*pAkt:Hsp90 - konHsp90_CaMeNOS*Arg:CN:CaM:eNOS*pAkt:Hsp90 + koffHsp90eNOS*eNOS:Hsp90:pAkt + koffHsp90eNOS*Arg:eNOS:Hsp90:pAkt + koffHsp90_CaMeNOS*C:CaM:eNOS:Hsp90:pAkt + koffHsp90_CaMeNOS*Arg:C:CaM:eNOS:Hsp90:pAkt + koffHsp90_CaMeNOS*N:CaM:eNOS:Hsp90:pAkt + koffHsp90_CaMeNOS*Arg:N:CaM:eNOS:Hsp90:pAkt + koffHsp90_CaMeNOS*CN:CaM:eNOS:Hsp90:pAkt + koffHsp90_CaMeNOS*Arg:CN:CaM:eNOS:Hsp90:pAkt ;

d(Akt:pHsp90)/dt = kdpAkt*pAkt:pHsp90 - kdpHsp90*Akt:pHsp90 - konHsp90eNOS*eNOS*Akt:pHsp90 - konHsp90eNOS*Arg:eNOS*Akt:pHsp90 - konHsp90_CaMeNOS*C:CaM:eNOS*Akt:pHsp90 - konHsp90_CaMeNOS*Arg:C:CaM:eNOS*Akt:pHsp90 - konHsp90_CaMeNOS*N:CaM:eNOS*Akt:pHsp90 - konHsp90_CaMeNOS*Arg:N:CaM:eNOS*Akt:pHsp90 - konHsp90_CaMeNOS*CN:CaM:eNOS*Akt:pHsp90 - konHsp90_CaMeNOS*Arg:CN:CaM:eNOS*Akt:pHsp90 + koffHsp90eNOS*eNOS:pHsp90:Akt + koffHsp90eNOS*Arg:eNOS:pHsp90:Akt + koffHsp90_CaMeNOS*C:CaM:eNOS:pHsp90:Akt + koffHsp90_CaMeNOS*Arg:C:CaM:eNOS:pHsp90:Akt + koffHsp90_CaMeNOS*N:CaM:eNOS:pHsp90:Akt + koffHsp90_CaMeNOS*Arg:N:CaM:eNOS:pHsp90:Akt + koffHsp90_CaMeNOS*CN:CaM:eNOS:pHsp90:Akt + koffHsp90_CaMeNOS*Arg:CN:CaM:eNOS:pHsp90:Akt ;

d(eNOS:pHsp90:pAkt)/dt = konHsp90eNOS*eNOS*pAkt:pHsp90 + konpAktHsp90*eNOS:pHsp90*pAkt + konHsp90eNOS*eNOS:pAkt*pHsp90 - kdpAkt*eNOS:pHsp90:pAkt - kon2CeNOS_H*C:CaM*eNOS:pHsp90:pAkt + koffCaMeNOS_H*C:CaM:eNOS:pHsp90:pAkt - kon2NeNOS_H*N:CaM*eNOS:pHsp90:pAkt + kon2NeNOS_H*N:CaM*Arg:eNOS:pHsp90:pAkt - kon4eNOS_H*CN:CaM*eNOS:pHsp90:pAkt + koffCaMeNOS_H*CN:CaM:eNOS:pHsp90:pAkt - kon2CpeNOS_H*C:CaM*eNOS:pHsp90:pAkt + koffCaMpeNOS_H*C:CaM:eNOS:pHsp90:pAkt - kon2NpeNOS_H*N:CaM*eNOS:pHsp90:pAkt + koffCaMpeNOS_H*N:CaM:eNOS:pHsp90:pAkt - kon4peNOS_H*CN:CaM*eNOS:pHsp90:pAkt + koffCaMpeNOS_H*CN:CaM:eNOS:pHsp90:pAkt - koffHsp90eNOS*eNOS:pHsp90:pAkt - koffpAktHsp90*eNOS:pHsp90:pAkt - koffHsp90eNOS*eNOS:pHsp90:pAkt - konbeNOSArg*Arg*eNOS:pHsp90:pAkt + koffbeNOSArg*Arg:eNOS:pHsp90:pAkt ;

d(Arg:eNOS:pHsp90:pAkt)/dt = konHsp90eNOS*Arg:eNOS*pAkt:pHsp90 + konpAktHsp90*Arg:eNOS:pHsp90*pAkt + konHsp90eNOS*Arg:eNOS:pAkt*pHsp90 - kdpAkt*Arg:eNOS:pHsp90:pAkt - kon2CeNOS_H*C:CaM*Arg:eNOS:pHsp90:pAkt + koffCaMeNOS_H*Arg:C:CaM:eNOS:pHsp90:pAkt - kon2NeNOS_H*N:CaM*Arg:eNOS:pHsp90:pAkt + koffCaMeNOS_H*Arg:N:CaM:eNOS:pHsp90:pAkt - kon4eNOS_H*CN:CaM*Arg:eNOS:pHsp90:pAkt + koffCaMeNOS_H*Arg:CN:CaM:eNOS:pHsp90:pAkt - kon2CpeNOS_H*C:CaM*Arg:eNOS:pHsp90:pAkt + koffCaMpeNOS_H*Arg:C:CaM:eNOS:pHsp90:pAkt - kon2NpeNOS_H*N:CaM*Arg:eNOS:pHsp90:pAkt + koffCaMpeNOS_H*Arg:N:CaM:eNOS:pHsp90:pAkt -

kon4peNOS_H*CN:CaM*Arg:eNOS:pHsp90:pAkt + koffCaMpeNOS_H*Arg:CN:CaM:eNOS:pHsp90:pAkt - koffHsp90eNOS*Arg:eNOS:pHsp90:pAkt - koffpAktHsp90*Arg:eNOS:pHsp90:pAkt - koffHsp90eNOS*Arg:eNOS:pHsp90:pAkt + konbeNOSArg*Arg*eNOS:pHsp90:pAkt - koffbeNOSArg*Arg:eNOS:pHsp90:pAkt ;

d(C:CaM:eNOS:pHsp90:pAkt)/dt = konpAktHsp90*C:CaM:eNOS:pHsp90*pAkt + konHsp90_CaMeNOS*C:CaM:eNOS:pAkt*pHsp90 + konHsp90_CaMeNOS*C:CaM:eNOS*pAkt:pHsp90 - kdpAkt*C:CaM:eNOS:pHsp90:pAkt - konCa2N*Ca*Ca*C:CaM:eNOS:pHsp90:pAkt + koffCa2N*CN:CaM:eNOS:pHsp90:pAkt + kon2CeNOS_H*C:CaM*eNOS:pHsp90:pAkt - koffCaMeNOS_H*C:CaM:eNOS:pHsp90:pAkt + kon2CpeNOS_H*C:CaM*eNOS:pHsp90:pAkt - koffCaMpeNOS_H*C:CaM:eNOS:pHsp90:pAkt - koffpAktHsp90*C:CaM:eNOS:pHsp90:pAkt - koffHsp90_CaMeNOS*C:CaM:eNOS:pHsp90:pAkt - koffHsp90_CaMeNOS*C:CaM:eNOS:pHsp90:pAkt - kcatpAkt_H*C:CaM:eNOS:pHsp90:pAkt - koneNOSArg_H*Arg*C:CaM:eNOS:pHsp90:pAkt + koffeNOSArg_H*Arg:C:CaM:eNOS:pHsp90:pAkt + kcateNOS_H*Arg:C:CaM:eNOS:pHsp90:pAkt ;

d(Arg:C:CaM:eNOS:pHsp90:pAkt)/dt = konpAktHsp90*Arg:C:CaM:eNOS:pHsp90*pAkt + konHsp90_CaMeNOS*Arg:C:CaM:eNOS:pAkt*pHsp90 + konHsp90_CaMeNOS*Arg:C:CaM:eNOS*pAkt:pHsp90 - kdpAkt*Arg:C:CaM:eNOS:pHsp90:pAkt - konCa2N*Ca*Ca*Arg:C:CaM:eNOS:pHsp90:pAkt + koffCa2N*Arg:CN:CaM:eNOS:pHsp90:pAkt + kon2CeNOS_H*C:CaM*Arg:eNOS:pHsp90:pAkt - koffCaMeNOS_H*Arg:C:CaM:eNOS:pHsp90:pAkt + kon2CpeNOS_H*C:CaM*Arg:eNOS:pHsp90:pAkt - koffCaMpeNOS_H*Arg:C:CaM:eNOS:pHsp90:pAkt - koffpAktHsp90*Arg:C:CaM:eNOS:pHsp90:pAkt - koffHsp90_CaMeNOS*Arg:C:CaM:eNOS:pHsp90:pAkt - koffHsp90_CaMeNOS*Arg:C:CaM:eNOS:pHsp90:pAkt - kcatpAkt_H*Arg:C:CaM:eNOS:pHsp90:pAkt + koneNOSArg_H*Arg*C:CaM:eNOS:pHsp90:pAkt - koffeNOSArg_H*Arg:C:CaM:eNOS:pHsp90:pAkt - kcateNOS_H*Arg:C:CaM:eNOS:pHsp90:pAkt ;

d(N:CaM:eNOS:pHsp90:pAkt)/dt = konpAktHsp90*N:CaM:eNOS:pHsp90*pAkt + konHsp90_CaMeNOS*N:CaM:eNOS:pAkt*pHsp90 + konHsp90_CaMeNOS*N:CaM:eNOS*pAkt:pHsp90 - kdpAkt*N:CaM:eNOS:pHsp90:pAkt - konCa2C*Ca*Ca*N:CaM:eNOS:pHsp90:pAkt + koffCa2C*CN:CaM:eNOS:pHsp90:pAkt + kon2NeNOS_H*N:CaM*eNOS:pHsp90:pAkt - kon2NeNOS_H*N:CaM*Arg:eNOS:pHsp90:pAkt + kon2NpeNOS_H*N:CaM*eNOS:pHsp90:pAkt - koffCaMpeNOS_H*N:CaM:eNOS:pHsp90:pAkt - koffpAktHsp90*N:CaM:eNOS:pHsp90:pAkt - koffHsp90_CaMeNOS*N:CaM:eNOS:pHsp90:pAkt - koffHsp90_CaMeNOS*N:CaM:eNOS:pHsp90:pAkt - kcatpAkt_H*N:CaM:eNOS:pHsp90:pAkt - koneNOSArg_H*Arg*N:CaM:eNOS:pHsp90:pAkt + koffeNOSArg_H*Arg:N:CaM:eNOS:pHsp90:pAkt + kcateNOS_H*Arg:N:CaM:eNOS:pHsp90:pAkt ;

d(Arg:N:CaM:eNOS:pHsp90:pAkt)/dt = konpAktHsp90*Arg:N:CaM:eNOS:pHsp90*pAkt + konHsp90_CaMeNOS*Arg:N:CaM:eNOS:pAkt*pHsp90 + konHsp90_CaMeNOS*Arg:N:CaM:eNOS*pAkt:pHsp90 - kdpAkt*Arg:N:CaM:eNOS:pHsp90:pAkt - konCa2C*Ca*Ca*Arg:N:CaM:eNOS:pHsp90:pAkt + koffCa2C*Arg:CN:CaM:eNOS:pHsp90:pAkt + kon2NeNOS_H*N:CaM*Arg:eNOS:pHsp90:pAkt - koffCaMeNOS_H*Arg:N:CaM:eNOS:pHsp90:pAkt + kon2NpeNOS_H*N:CaM*Arg:eNOS:pHsp90:pAkt - koffCaMpeNOS_H*Arg:N:CaM:eNOS:pHsp90:pAkt - koffpAktHsp90*Arg:N:CaM:eNOS:pHsp90:pAkt - koffHsp90_CaMeNOS*Arg:N:CaM:eNOS:pHsp90:pAkt - koffHsp90_CaMeNOS*Arg:N:CaM:eNOS:pHsp90:pAkt - kcatpAkt_H*Arg:N:CaM:eNOS:pHsp90:pAkt + koneNOSArg_H*Arg*N:CaM:eNOS:pHsp90:pAkt - koffeNOSArg_H*Arg:N:CaM:eNOS:pHsp90:pAkt - kcateNOS_H*Arg:N:CaM:eNOS:pHsp90:pAkt ;

d(CN:CaM:eNOS:pHsp90:pAkt)/dt = konpAktHsp90*CN:CaM:eNOS:pHsp90*pAkt + konHsp90_CaMeNOS*CN:CaM:eNOS:pAkt*pHsp90 + konHsp90_CaMeNOS*CN:CaM:eNOS*pAkt:pHsp90 - kdpAkt*CN:CaM:eNOS:pHsp90:pAkt + konCa2C*Ca*Ca*N:CaM:eNOS:pHsp90:pAkt - koffCa2C*CN:CaM:eNOS:pHsp90:pAkt + konCa2N*Ca*Ca*C:CaM:eNOS:pHsp90:pAkt - koffCa2N*CN:CaM:eNOS:pHsp90:pAkt + kon4eNOS_H*CN:CaM*eNOS:pHsp90:pAkt - koffCaMeNOS_H*CN:CaM:eNOS:pHsp90:pAkt + kon4peNOS_H*CN:CaM*eNOS:pHsp90:pAkt - koffCaMpeNOS_H*CN:CaM:eNOS:pHsp90:pAkt - koffpAktHsp90*CN:CaM:eNOS:pHsp90:pAkt - koffHsp90_CaMeNOS*CN:CaM:eNOS:pHsp90:pAkt - koffHsp90_CaMeNOS*CN:CaM:eNOS:pHsp90:pAkt - kcatpAkt_H*CN:CaM:eNOS:pHsp90:pAkt - koneNOSArg_H*Arg*CN:CaM:eNOS:pHsp90:pAkt + koffeNOSArg_H*Arg:CN:CaM:eNOS:pHsp90:pAkt + kcateNOS_H*Arg:CN:CaM:eNOS:pHsp90:pAkt ;

d(Arg:CN:CaM:eNOS:pHsp90:pAkt)/dt = konpAktHsp90*Arg:CN:CaM:eNOS:pHsp90*pAkt + konHsp90_CaMeNOS*Arg:CN:CaM:eNOS:pAkt*pHsp90 + konHsp90_CaMeNOS*Arg:CN:CaM:eNOS*pAkt:pHsp90 - kdpAkt*Arg:CN:CaM:eNOS:pHsp90:pAkt + konCa2C*Ca*Ca*Arg:N:CaM:eNOS:pHsp90:pAkt - koffCa2C*Arg:CN:CaM:eNOS:pHsp90:pAkt + konCa2N*Ca*Ca*Arg:C:CaM:eNOS:pHsp90:pAkt - koffCa2N*Arg:CN:CaM:eNOS:pHsp90:pAkt + kon4eNOS_H*CN:CaM*Arg:eNOS:pHsp90:pAkt -

koffCaMeNOS_H*Arg:CN:CaM:eNOS:pHsp90:pAkt + kon4peNOS_H*CN:CaM*Arg:eNOS:pHsp90:pAkt - koffCaMpeNOS_H*Arg:CN:CaM:eNOS:pHsp90:pAkt - koffpAktHsp90*Arg:CN:CaM:eNOS:pHsp90:pAkt - koffHsp90_CaMeNOS*Arg:CN:CaM:eNOS:pHsp90:pAkt -koffHsp90_CaMeNOS*Arg:CN:CaM:eNOS:pHsp90:pAkt - kcatpAkt_H*Arg:CN:CaM:eNOS:pHsp90:pAkt + koneNOSArg_H*Arg*CN:CaM:eNOS:pHsp90:pAkt - koffeNOSArg_H*Arg:CN:CaM:eNOS:pHsp90:pAkt - kcateNOS_H*Arg:CN:CaM:eNOS:pHsp90:pAkt ;

d(C:CaM:peNOS)/dt = kcatpAkt*C:CaM:eNOS:pAkt - konCa2N*Ca*Ca*C:CaM:peNOS + koffCa2N*CN:CaM:peNOS - koffCaMpeNOS*C:CaM:peNOS - konHsp90_CaMeNOS*C:CaM:peNOS*pHsp90 - kdpeNOS*C:CaM:peNOS - kdegpeNOS*C:CaM:peNOS - konpeNOSArg*Arg*C:CaM:peNOS + koffpeNOSArg*Arg:C:CaM:peNOS + kcatpeNOS*Arg:C:CaM:peNOS + kon2CpeNOS*C:CaM*peNOS + koffHsp90_CaMeNOS*C:CaM:peNOS:pHsp90 ;

d(Arg:C:CaM:peNOS)/dt = kcatpAkt*Arg:C:CaM:eNOS:pAkt - konCa2N*Ca*Ca*Arg:C:CaM:peNOS + koffCa2N*Arg:CN:CaM:peNOS - koffCaMpeNOS*Arg:C:CaM:peNOS - konHsp90_CaMeNOS*Arg:C:CaM:peNOS*pHsp90 - kdpeNOS*Arg:C:CaM:peNOS + konpeNOSArg*Arg*C:CaM:peNOS - koffpeNOSArg*Arg:C:CaM:peNOS - kcatpeNOS*Arg:C:CaM:peNOS + kon2CpeNOS*C:CaM*Arg:peNOS + koffHsp90_CaMeNOS*Arg:C:CaM:peNOS:pHsp90 ;

d(N:CaM:peNOS)/dt = kcatpAkt*N:CaM:eNOS:pAkt - konCa2C*Ca*Ca*N:CaM:peNOS + koffCa2C*CN:CaM:peNOS - koffCaMpeNOS*N:CaM:peNOS - konHsp90_CaMeNOS*N:CaM:peNOS*pHsp90 - kdpeNOS*N:CaM:peNOS - kdegpeNOS*N:CaM:peNOS - konpeNOSArg*Arg*N:CaM:peNOS + koffpeNOSArg*Arg:N:CaM:peNOS + kcatpeNOS*Arg:N:CaM:peNOS + kon2NpeNOS*N:CaM*peNOS + koffHsp90_CaMeNOS*N:CaM:peNOS:pHsp90 ;

d(Arg:N:CaM:peNOS)/dt = kcatpAkt*Arg:N:CaM:eNOS:pAkt - konCa2C*Ca*Ca*Arg:N:CaM:peNOS + koffCa2C*Arg:CN:CaM:peNOS - koffCaMpeNOS*Arg:N:CaM:peNOS - konHsp90_CaMeNOS*Arg:N:CaM:peNOS*pHsp90 - kdpeNOS*Arg:N:CaM:peNOS + konpeNOSArg*Arg*N:CaM:peNOS - koffpeNOSArg*Arg:N:CaM:peNOS - kcatpeNOS*Arg:N:CaM:peNOS + kon2NpeNOS*N:CaM*Arg:peNOS + koffHsp90_CaMeNOS*N:CaM:peNOS:pHsp90 ;

d(CN:CaM:peNOS)/dt = kcatpAkt*CN:CaM:eNOS:pAkt + konCa2C*Ca*Ca*N:CaM:peNOS - koffCa2C*CN:CaM:peNOS + konCa2N*Ca*Ca*C:CaM:peNOS - koffCa2N*CN:CaM:peNOS - koffCaMpeNOS*CN:CaM:peNOS - konHsp90_CaMeNOS*CN:CaM:peNOS*pHsp90 - kdpeNOS*CN:CaM:peNOS - kdegpeNOS*CN:CaM:peNOS - konpeNOSArg*Arg*CN:CaM:peNOS + koffpeNOSArg*Arg:CN:CaM:peNOS + kcatpeNOS*Arg:CN:CaM:peNOS + kon4peNOS*CN:CaM*peNOS + koffHsp90_CaMeNOS*CN:CaM:peNOS:pHsp90 ;

d(Arg:CN:CaM:peNOS)/dt = kcatpAkt*Arg:CN:CaM:eNOS:pAkt + konCa2C*Ca*Ca*Arg:N:CaM:peNOS - koffCa2C*Arg:CN:CaM:peNOS + konCa2N*Ca*Ca*Arg:C:CaM:peNOS - koffCa2N*Arg:CN:CaM:peNOS - koffCaMpeNOS*CN:CaM:peNOS - konHsp90_CaMeNOS*Arg:CN:CaM:peNOS*pHsp90 - kdpeNOS*Arg:CN:CaM:peNOS + konpeNOSArg*Arg*CN:CaM:peNOS - koffpeNOSArg*Arg:CN:CaM:peNOS - kcatpeNOS*Arg:CN:CaM:peNOS + kon4peNOS*CN:CaM*Arg:peNOS + koffHsp90_CaMeNOS*Arg:CN:CaM:peNOS:pHsp90 ;

d(cGMP:PDE_a)/dt = ka_PDE*cGMP:PDE - kd_PDE*cGMP:PDE_a ;

d(Akt:Hsp90)/dt = kdpHsp90*Akt:pHsp90 + kdpAkt*pAkt:Hsp90 - konHsp90eNOS*eNOS*Akt:Hsp90 - konHsp90eNOS*Arg:eNOS*Akt:Hsp90 - konHsp90_CaMeNOS*C:CaM:eNOS*Akt:Hsp90 - konHsp90_CaMeNOS*Arg:C:CaM:eNOS*Akt:Hsp90 - konHsp90_CaMeNOS*N:CaM:eNOS*Akt:Hsp90 - konHsp90_CaMeNOS*Arg:N:CaM:eNOS*Akt:Hsp90 - konHsp90_CaMeNOS*CN:CaM:eNOS*Akt:Hsp90 - konHsp90_CaMeNOS*Arg:CN:CaM:eNOS*Akt:Hsp90 + koffHsp90eNOS*eNOS:Hsp90:Akt + koffHsp90eNOS*Arg:eNOS:Hsp90:Akt + koffHsp90_CaMeNOS*C:CaM:eNOS:Hsp90:Akt + koffHsp90_CaMeNOS*Arg:C:CaM:eNOS:Hsp90:Akt + koffHsp90_CaMeNOS*N:CaM:eNOS:Hsp90:Akt + koffHsp90_CaMeNOS*Arg:N:CaM:eNOS:Hsp90:Akt + koffHsp90_CaMeNOS*CN:CaM:eNOS:Hsp90:Akt + koffHsp90_CaMeNOS*Arg:CN:CaM:eNOS:Hsp90:Akt ;

d(eNOS:pHsp90:Akt)/dt = kdpAkt*eNOS:pHsp90:pAkt + konHsp90eNOS*eNOS*Akt:pHsp90 - kon2CeNOS_H*C:CaM*eNOS:pHsp90:Akt + koffCaMeNOS_H*C:CaM:eNOS:pHsp90:Akt - kon2NeNOS_H*N:CaM*eNOS:pHsp90:Akt + koffCaMeNOS_H*N:CaM:eNOS:pHsp90:Akt - kon4eNOS_H*CN:CaM*eNOS:pHsp90:Akt + koffCaMeNOS_H*CN:CaM:eNOS:pHsp90:Akt - kon2CpeNOS_H*C:CaM*eNOS:pHsp90:Akt + koffCaMpeNOS_H*C:CaM:eNOS:pHsp90:Akt - kon2NpeNOS_H*N:CaM*eNOS:pHsp90:Akt + koffCaMpeNOS_H*N:CaM:eNOS:pHsp90:Akt - kon4peNOS_H*CN:CaM*eNOS:pHsp90:Akt + koffCaMpeNOS_H*CN:CaM:eNOS:pHsp90:Akt - koffHsp90eNOS*eNOS:pHsp90:Akt - koffHsp90eNOS*eNOS:pHsp90:Akt - konbeNOSArg*Arg*eNOS:pHsp90:Akt + koffbeNOSArg*Arg:eNOS:pHsp90:Akt + konHsp90eNOS*Akt:eNOS*pHsp90 ;

d(Arg:eNOS:pHsp90:Akt)/dt = kdpAkt*Arg:eNOS:pHsp90:pAkt + konHsp90eNOS*Arg:eNOS*Akt:pHsp90 - kon2CeNOS_H*C:CaM*Arg:eNOS:pHsp90:Akt + koffCaMeNOS_H*Arg:C:CaM:eNOS:pHsp90:Akt - kon2NeNOS_H*N:CaM*Arg:eNOS:pHsp90:Akt + koffCaMeNOS_H*Arg:N:CaM:eNOS:pHsp90:Akt - kon4eNOS_H*CN:CaM*Arg:eNOS:pHsp90:Akt + koffCaMeNOS_H*Arg:CN:CaM:eNOS:pHsp90:Akt - kon2CpeNOS_H*C:CaM*Arg:eNOS:pHsp90:Akt + koffCaMpeNOS_H*Arg:C:CaM:eNOS:pHsp90:Akt - kon2NpeNOS_H*N:CaM*Arg:eNOS:pHsp90:Akt + koffCaMpeNOS_H*Arg:N:CaM:eNOS:pHsp90:Akt - kon4peNOS_H*CN:CaM*Arg:eNOS:pHsp90:Akt + koffCaMpeNOS_H*Arg:CN:CaM:eNOS:pHsp90:Akt - koffHsp90eNOS*Arg:eNOS:pHsp90:Akt - koffHsp90eNOS*Arg:eNOS:pHsp90:Akt + konbeNOSArg*Arg*eNOS:pHsp90:Akt - koffbeNOSArg*Arg:eNOS:pHsp90:Akt + konHsp90eNOS*Arg:Akt:eNOS*pHsp90 ;

d(C:CaM:eNOS:pHsp90:Akt)/dt = kdpAkt*C:CaM:eNOS:pHsp90:pAkt + konHsp90_CaMeNOS*C:CaM:eNOS*Akt:pHsp90 - konCa2N*Ca*Ca*C:CaM:eNOS:pHsp90:Akt + koffCa2N*CN:CaM:eNOS:pHsp90:Akt + kon2CeNOS_H*C:CaM*eNOS:pHsp90:Akt - koffCaMeNOS_H*C:CaM:eNOS:pHsp90:Akt + kon2CpeNOS_H*C:CaM*eNOS:pHsp90:Akt - koffCaMpeNOS_H*C:CaM:eNOS:pHsp90:Akt - koffHsp90_CaMeNOS*C:CaM:eNOS:pHsp90:Akt - koffHsp90_CaMeNOS*C:CaM:eNOS:pHsp90:Akt - koneNOSArg_H*Arg*C:CaM:eNOS:pHsp90:Akt + koffeNOSArg_H*Arg:C:CaM:eNOS:pHsp90:Akt + kcateNOS_H*Arg:C:CaM:eNOS:pHsp90:Akt + konHsp90_CaMeNOS*C:CaM:eNOS:Akt*pHsp90 ;

d(Arg:C:CaM:eNOS:pHsp90:Akt)/dt = kdpAkt*Arg:C:CaM:eNOS:pHsp90:pAkt + konHsp90_CaMeNOS*Arg:C:CaM:eNOS*Akt:pHsp90 - konCa2N*Ca*Ca*Arg:C:CaM:eNOS:pHsp90:Akt + koffCa2N*Arg:CN:CaM:eNOS:pHsp90:Akt + kon2CeNOS_H*C:CaM*Arg:eNOS:pHsp90:Akt - koffCaMeNOS_H*Arg:C:CaM:eNOS:pHsp90:Akt + kon2CpeNOS_H*C:CaM*Arg:eNOS:pHsp90:Akt - koffCaMpeNOS_H*Arg:C:CaM:eNOS:pHsp90:Akt - koffHsp90_CaMeNOS*Arg:C:CaM:eNOS:pHsp90:Akt - koffHsp90_CaMeNOS*Arg:C:CaM:eNOS:pHsp90:Akt + koneNOSArg_H*Arg*C:CaM:eNOS:pHsp90:Akt - koffeNOSArg_H*Arg:C:CaM:eNOS:pHsp90:Akt - kcateNOS_H*Arg:C:CaM:eNOS:pHsp90:Akt + konHsp90_CaMeNOS*Arg:C:CaM:eNOS:Akt*pHsp90 ;

d(N:CaM:eNOS:pHsp90:Akt)/dt = kdpAkt*N:CaM:eNOS:pHsp90:pAkt + konHsp90_CaMeNOS*N:CaM:eNOS*Akt:pHsp90 - konCa2C*Ca*Ca*N:CaM:eNOS:pHsp90:Akt + koffCa2C*CN:CaM:eNOS:pHsp90:Akt + kon2NeNOS_H*N:CaM*eNOS:pHsp90:Akt - koffCaMeNOS_H*N:CaM:eNOS:pHsp90:Akt + kon2NpeNOS_H*N:CaM*eNOS:pHsp90:Akt - koffCaMpeNOS_H*N:CaM:eNOS:pHsp90:Akt - koffHsp90_CaMeNOS*N:CaM:eNOS:pHsp90:Akt - koffHsp90_CaMeNOS*N:CaM:eNOS:pHsp90:Akt - koneNOSArg_H*Arg*N:CaM:eNOS:pHsp90:Akt + koffeNOSArg_H*Arg:N:CaM:eNOS:pHsp90:Akt + kcateNOS_H*Arg:N:CaM:eNOS:pHsp90:Akt + konHsp90_CaMeNOS*N:CaM:eNOS:Akt*pHsp90 ;

d(Arg:N:CaM:eNOS:pHsp90:Akt)/dt = kdpAkt*Arg:N:CaM:eNOS:pHsp90:pAkt + konHsp90_CaMeNOS*Arg:N:CaM:eNOS*Akt:pHsp90 - konCa2C*Ca*Ca*Arg:N:CaM:eNOS:pHsp90:Akt + koffCa2C*Arg:CN:CaM:eNOS:pHsp90:Akt + kon2NeNOS_H*N:CaM*Arg:eNOS:pHsp90:Akt - koffCaMeNOS_H*Arg:N:CaM:eNOS:pHsp90:Akt + kon2NpeNOS_H*N:CaM*Arg:eNOS:pHsp90:Akt - koffCaMpeNOS_H*Arg:N:CaM:eNOS:pHsp90:Akt - koffHsp90_CaMeNOS*Arg:N:CaM:eNOS:pHsp90:Akt - koffHsp90_CaMeNOS*Arg:N:CaM:eNOS:pHsp90:Akt + koneNOSArg_H*Arg*N:CaM:eNOS:pHsp90:Akt - koffeNOSArg_H*Arg:N:CaM:eNOS:pHsp90:Akt - kcateNOS_H*Arg:N:CaM:eNOS:pHsp90:Akt + konHsp90_CaMeNOS*Arg:N:CaM:eNOS:Akt*pHsp90 ;

d(CN:CaM:eNOS:pHsp90:Akt)/dt = kdpAkt*CN:CaM:eNOS:pHsp90:pAkt + konHsp90_CaMeNOS*CN:CaM:eNOS*Akt:pHsp90 + konCa2C*Ca*Ca*N:CaM:eNOS:pHsp90:Akt - koffCa2C*CN:CaM:eNOS:pHsp90:Akt + konCa2N*Ca*Ca*C:CaM:eNOS:pHsp90:Akt - koffCa2N*CN:CaM:eNOS:pHsp90:Akt + kon4eNOS_H*CN:CaM*eNOS:pHsp90:Akt - koffCaMeNOS_H*CN:CaM:eNOS:pHsp90:Akt + kon4peNOS_H*CN:CaM*eNOS:pHsp90:Akt - koffCaMpeNOS_H*CN:CaM:eNOS:pHsp90:Akt - koffHsp90_CaMeNOS*CN:CaM:eNOS:pHsp90:Akt - koffHsp90_CaMeNOS*CN:CaM:eNOS:pHsp90:Akt - koneNOSArg_H*Arg*CN:CaM:eNOS:pHsp90:Akt + koffeNOSArg_H*Arg:CN:CaM:eNOS:pHsp90:Akt + kcateNOS_H*Arg:CN:CaM:eNOS:pHsp90:Akt + konHsp90_CaMeNOS*CN:CaM:eNOS:Hsp90:Akt*pHsp90 ;

d(Arg:CN:CaM:eNOS:pHsp90:Akt)/dt = kdpAkt*Arg:CN:CaM:eNOS:pHsp90:pAkt + konHsp90_CaMeNOS*Arg:CN:CaM:eNOS*Akt:pHsp90 + konCa2C*Ca*Ca*Arg:N:CaM:eNOS:pHsp90:Akt - koffCa2C*Arg:CN:CaM:eNOS:pHsp90:Akt + konCa2N*Ca*Ca*Arg:C:CaM:eNOS:pHsp90:Akt - koffCa2N*Arg:CN:CaM:eNOS:pHsp90:Akt + kon4eNOS_H*CN:CaM*Arg:eNOS:pHsp90:Akt - koffCaMeNOS_H*Arg:CN:CaM:eNOS:pHsp90:Akt + kon4peNOS_H*CN:CaM*Arg:eNOS:pHsp90:Akt - koffCaMpeNOS_H*Arg:CN:CaM:eNOS:pHsp90:Akt - koffHsp90_CaMeNOS*Arg:CN:CaM:eNOS:pHsp90:Akt - koffHsp90_CaMeNOS*Arg:CN:CaM:eNOS:pHsp90:Akt + koneNOSArg_H*Arg*CN:CaM:eNOS:pHsp90:Akt -

koffeNOSArg_H*Arg:CN:CaM:eNOS:pHsp90:Akt - kcateNOS_H*Arg:CN:CaM:eNOS:pHsp90:Akt + konHsp90_CaMeNOS*Arg:CN:CaM:eNOS:Hsp90:Akt*pHsp90 ;

d(peNOS)/dt = koffCaMpeNOS*C:CaM:peNOS + koffCaMpeNOS*N:CaM:peNOS + koffCaMpeNOS*CN:CaM:peNOS - kon2CpeNOS*C:CaM*peNOS - kon2NpeNOS*N:CaM*peNOS - kon4peNOS*CN:CaM*peNOS - konHsp90eNOS*peNOS*pHsp90 - kdpeNOS*peNOS - kdegpeNOS*peNOS - konpeNOSArg*Arg*peNOS + koffpeNOSArg*Arg:peNOS + kcatpeNOS*Arg:peNOS + koffHsp90eNOS*peNOS:pHsp90 ;

d(Arg:peNOS)/dt = koffCaMpeNOS*Arg:C:CaM:peNOS + koffCaMpeNOS*Arg:N:CaM:peNOS + koffCaMpeNOS*CN:CaM:peNOS - kon2CpeNOS*C:CaM*Arg:peNOS - kon2NpeNOS*N:CaM*Arg:peNOS - kon4peNOS*CN:CaM*Arg:peNOS - konHsp90eNOS*Arg:peNOS*pHsp90 - kdpeNOS*Arg:peNOS + konpeNOSArg*Arg*peNOS - koffpeNOSArg*Arg:peNOS - kcatpeNOS*Arg:peNOS + koffHsp90eNOS*Arg:peNOS:pHsp90 ;

d(C:CaM:peNOS:pHsp90)/dt = konHsp90_CaMeNOS*C:CaM:peNOS*pHsp90 + kcatpAkt_H*C:CaM:eNOS:pHsp90:pAkt - konCa2N*Ca*Ca*C:CaM:peNOS:pHsp90 + koffCa2N*CN:CaM:peNOS:pHsp90 - koffCaMpeNOS_H*C:CaM:peNOS:pHsp90 - koffHsp90_CaMeNOS*C:CaM:peNOS:pHsp90 - kdpeNOS*C:CaM:peNOS:pHsp90 - konpeNOSArg_H*Arg*C:CaM:peNOS:pHsp90 + koffpeNOSArg_H*Arg:C:CaM:peNOS:pHsp90 + kcatpeNOS_H*Arg:C:CaM:peNOS:pHsp90 + kon2CpeNOS_H*C:CaM*peNOS:pHsp90 ;

d(Arg:C:CaM:peNOS:pHsp90)/dt = konHsp90_CaMeNOS*Arg:C:CaM:peNOS*pHsp90 + kcatpAkt_H*Arg:C:CaM:eNOS:pHsp90:pAkt - konCa2N*Ca*Ca*Arg:C:CaM:peNOS:pHsp90 + koffCa2N*Arg:CN:CaM:peNOS:pHsp90 - koffCaMpeNOS_H*Arg:C:CaM:peNOS:pHsp90 - koffHsp90_CaMeNOS*Arg:C:CaM:peNOS:pHsp90 - kdpeNOS*Arg:C:CaM:peNOS:pHsp90 + konpeNOSArg_H*Arg*C:CaM:peNOS:pHsp90 - koffpeNOSArg_H*Arg:C:CaM:peNOS:pHsp90 - kcatpeNOS_H*Arg:C:CaM:peNOS:pHsp90 + kon2CpeNOS_H*C:CaM*Arg:peNOS:pHsp90 ;

d(N:CaM:peNOS:pHsp90)/dt = konHsp90_CaMeNOS*N:CaM:peNOS*pHsp90 + kcatpAkt_H*N:CaM:eNOS:pHsp90:pAkt - konCa2C*Ca*Ca*N:CaM:peNOS:pHsp90 + koffCa2C*CN:CaM:peNOS:pHsp90 - koffCaMpeNOS_H*N:CaM:peNOS:pHsp90 - koffHsp90_CaMeNOS*N:CaM:peNOS:pHsp90 - kdpeNOS*N:CaM:peNOS:pHsp90 - konpeNOSArg_H*Arg*N:CaM:peNOS:pHsp90 + koffpeNOSArg_H*Arg:N:CaM:peNOS:pHsp90 + kcatpeNOS_H*Arg:N:CaM:peNOS:pHsp90 + kon2NpeNOS_H*N:CaM*peNOS:pHsp90 ;

d(Arg:N:CaM:peNOS:pHsp90)/dt = konHsp90_CaMeNOS*Arg:N:CaM:peNOS*pHsp90 + kcatpAkt_H*Arg:N:CaM:eNOS:pHsp90:pAkt - konCa2C*Ca*Ca*Arg:N:CaM:peNOS:pHsp90 + koffCa2C*Arg:CN:CaM:peNOS:pHsp90 - koffCaMpeNOS_H*Arg:N:CaM:peNOS:pHsp90 - koffHsp90_CaMeNOS*N:CaM:peNOS:pHsp90 - kdpeNOS*Arg:N:CaM:peNOS:pHsp90 + konpeNOSArg_H*Arg*N:CaM:peNOS:pHsp90 - koffpeNOSArg_H*Arg:N:CaM:peNOS:pHsp90 - kcatpeNOS_H*Arg:N:CaM:peNOS:pHsp90 + kon2NpeNOS_H*N:CaM*Arg:peNOS:pHsp90 ;

d(CN:CaM:peNOS:pHsp90)/dt = konHsp90_CaMeNOS*CN:CaM:peNOS*pHsp90 + kcatpAkt_H*CN:CaM:eNOS:pHsp90:pAkt + konCa2C*Ca*Ca*N:CaM:peNOS:pHsp90 - koffCa2C*CN:CaM:peNOS:pHsp90 + konCa2N*Ca*Ca*C:CaM:peNOS:pHsp90 - koffCa2N*CN:CaM:peNOS:pHsp90 - koffCaMpeNOS_H*CN:CaM:peNOS:pHsp90 - koffHsp90_CaMeNOS*CN:CaM:peNOS:pHsp90 - kdpeNOS*CN:CaM:peNOS:pHsp90 - konpeNOSArg_H*Arg*CN:CaM:peNOS:pHsp90 + koffpeNOSArg_H*Arg:CN:CaM:peNOS:pHsp90 + kcatpeNOS_H*Arg:CN:CaM:peNOS:pHsp90 + kon4peNOS_H*CN:CaM*peNOS:pHsp90 ;

d(Arg:CN:CaM:peNOS:pHsp90)/dt = konHsp90_CaMeNOS*Arg:CN:CaM:peNOS*pHsp90 + kcatpAkt_H*Arg:CN:CaM:eNOS:pHsp90:pAkt + konCa2C*Ca*Ca*Arg:N:CaM:peNOS:pHsp90 - koffCa2C*Arg:CN:CaM:peNOS:pHsp90 + konCa2N*Ca*Ca*Arg:C:CaM:peNOS:pHsp90 - koffCa2N*Arg:CN:CaM:peNOS:pHsp90 - koffCaMpeNOS_H*Arg:CN:CaM:peNOS:pHsp90 - koffHsp90_CaMeNOS*Arg:CN:CaM:peNOS:pHsp90 - kdpeNOS*Arg:CN:CaM:peNOS:pHsp90 + konpeNOSArg_H*Arg*CN:CaM:peNOS:pHsp90 - koffpeNOSArg_H*Arg:CN:CaM:peNOS:pHsp90 - kcatpeNOS_H*Arg:CN:CaM:peNOS:pHsp90 + kon4peNOS_H*CN:CaM*Arg:peNOS:pHsp90 ;

d(eNOS:Hsp90:pAkt)/dt = konHsp90eNOS*eNOS*pAkt:Hsp90 - kdpAkt*eNOS:Hsp90:pAkt - kon2CeNOS_H*C:CaM*eNOS:Hsp90:pAkt + koffCaMeNOS_H*C:CaM:eNOS:Hsp90:pAkt - kon2NeNOS_H*N:CaM*eNOS:Hsp90:pAkt + koffCaMeNOS_H*N:CaM:eNOS:Hsp90:pAkt - kon4eNOS_H*CN:CaM*eNOS:Hsp90:pAkt + koffCaMeNOS_H*CN:CaM:eNOS:Hsp90:pAkt - koffHsp90eNOS*eNOS:Hsp90:pAkt - koffpAktHsp90*eNOS:Hsp90:pAkt - konbeNOSArg*Arg*eNOS:Hsp90:pAkt + koffbeNOSArg*Arg:eNOS:Hsp90:pAkt + konpAktHsp90*eNOS:Hsp90*pAkt ;

d(Arg:eNOS:Hsp90:pAkt)/dt = konHsp90eNOS*Arg:eNOS*pAkt:Hsp90 - kdpAkt*Arg:eNOS:Hsp90:pAkt -
kon2CeNOS_H*C:CaM*Arg:eNOS:Hsp90:pAkt + koffCaMeNOS_H*Arg:C:CaM:eNOS:Hsp90:pAkt -
kon2NeNOS_H*N:CaM*Arg:eNOS:Hsp90:pAkt + koffCaMeNOS_H*Arg:N:CaM:eNOS:Hsp90:pAkt -
kon4eNOS_H*CN:CaM*Arg:eNOS:Hsp90:pAkt + koffCaMeNOS_H*Arg:CN:CaM:eNOS:Hsp90:pAkt -
koffHsp90eNOS*Arg:eNOS:Hsp90:pAkt - koffpAktHsp90*Arg:eNOS:Hsp90:pAkt +
konbeNOSArg*Arg*eNOS:Hsp90:pAkt - koffbeNOSArg*Arg:eNOS:Hsp90:pAkt +
konpAktHsp90*Arg:eNOS:Hsp90*pAkt ;

d(C:CaM:eNOS:Hsp90:pAkt)/dt = konHsp90_CaMeNOS*C:CaM:eNOS*pAkt:Hsp90 - kdpAkt*C:CaM:eNOS:Hsp90:pAkt -
konCa2N*Ca*Ca*C:CaM:eNOS:Hsp90:pAkt + koffCa2N*CN:CaM:eNOS:Hsp90:pAkt +
kon2CeNOS_H*C:CaM*eNOS:Hsp90:pAkt - koffCaMeNOS_H*C:CaM:eNOS:Hsp90:pAkt -
koffpAktHsp90*C:CaM:eNOS:Hsp90:pAkt - koffHsp90_CaMeNOS*C:CaM:eNOS:Hsp90:pAkt -
koneNOSArg_H*Arg*C:CaM:eNOS:Hsp90:pAkt + koffeNOSArg_H*Arg:C:CaM:eNOS:Hsp90:pAkt +
kcateNOS_H*Arg:C:CaM:eNOS:Hsp90:pAkt + konpAktHsp90*C:CaM:eNOS:Hsp90*pAkt ;

d(Arg:C:CaM:eNOS:Hsp90:pAkt)/dt = konHsp90_CaMeNOS*Arg:C:CaM:eNOS*pAkt:Hsp90 -
kdpAkt*Arg:C:CaM:eNOS:Hsp90:pAkt - konCa2N*Ca*Ca*Arg:C:CaM:eNOS:Hsp90:pAkt +
koffCa2N*Arg:CN:CaM:eNOS:Hsp90:pAkt + kon2CeNOS_H*C:CaM*Arg:eNOS:Hsp90:pAkt -
koffCaMeNOS_H*Arg:C:CaM:eNOS:Hsp90:pAkt - koffpAktHsp90*Arg:C:CaM:eNOS:Hsp90:pAkt -
koffHsp90_CaMeNOS*Arg:C:CaM:eNOS:Hsp90:pAkt + koneNOSArg_H*Arg*C:CaM:eNOS:Hsp90:pAkt -
koffeNOSArg_H*Arg:C:CaM:eNOS:Hsp90:pAkt - kcateNOS_H*Arg:C:CaM:eNOS:Hsp90:pAkt +
konpAktHsp90*Arg:C:CaM:eNOS:Hsp90*pAkt ;

d(N:CaM:eNOS:Hsp90:pAkt)/dt = konHsp90_CaMeNOS*N:CaM:eNOS*pAkt:Hsp90 - kdpAkt*N:CaM:eNOS:Hsp90:pAkt -
konCa2C*Ca*Ca*N:CaM:eNOS:Hsp90:pAkt + koffCa2C*CN:CaM:eNOS:Hsp90:pAkt +
kon2NeNOS_H*N:CaM*eNOS:Hsp90:pAkt - koffCaMeNOS_H*N:CaM:eNOS:Hsp90:pAkt -
koffpAktHsp90*N:CaM:eNOS:Hsp90:pAkt - koffHsp90_CaMeNOS*N:CaM:eNOS:Hsp90:pAkt -
koneNOSArg_H*Arg*N:CaM:eNOS:Hsp90:pAkt + koffeNOSArg_H*Arg:N:CaM:eNOS:Hsp90:pAkt +
kcateNOS_H*Arg:N:CaM:eNOS:Hsp90:pAkt + konpAktHsp90*N:CaM:eNOS:Hsp90*pAkt ;

d(Arg:N:CaM:eNOS:Hsp90:pAkt)/dt = konHsp90_CaMeNOS*Arg:N:CaM:eNOS*pAkt:Hsp90 -
kdpAkt*Arg:N:CaM:eNOS:Hsp90:pAkt - konCa2C*Ca*Ca*Arg:N:CaM:eNOS:Hsp90:pAkt +
koffCa2C*Arg:CN:CaM:eNOS:Hsp90:pAkt + kon2NeNOS_H*N:CaM*Arg:eNOS:Hsp90:pAkt -
koffCaMeNOS_H*Arg:N:CaM:eNOS:Hsp90:pAkt - koffpAktHsp90*Arg:N:CaM:eNOS:Hsp90:pAkt -
koffHsp90_CaMeNOS*Arg:N:CaM:eNOS:Hsp90:pAkt + koneNOSArg_H*Arg*N:CaM:eNOS:Hsp90:pAkt -
koffeNOSArg_H*Arg:N:CaM:eNOS:Hsp90:pAkt - kcateNOS_H*Arg:N:CaM:eNOS:Hsp90:pAkt +
konpAktHsp90*Arg:N:CaM:eNOS:Hsp90*pAkt ;

d(CN:CaM:eNOS:Hsp90:pAkt)/dt = konHsp90_CaMeNOS*CN:CaM:eNOS*pAkt:Hsp90 - kdpAkt*CN:CaM:eNOS:Hsp90:pAkt +
konCa2C*Ca*Ca*N:CaM:eNOS:Hsp90:pAkt - koffCa2C*CN:CaM:eNOS:Hsp90:pAkt +
konCa2N*Ca*Ca*C:CaM:eNOS:Hsp90:pAkt - koffCa2N*CN:CaM:eNOS:Hsp90:pAkt +
kon4eNOS_H*CN:CaM*eNOS:Hsp90:pAkt - koffCaMeNOS_H*CN:CaM:eNOS:Hsp90:pAkt -
koffpAktHsp90*CN:CaM:eNOS:Hsp90:pAkt - koffHsp90_CaMeNOS*CN:CaM:eNOS:Hsp90:pAkt -
koneNOSArg_H*Arg*CN:CaM:eNOS:Hsp90:pAkt + koffeNOSArg_H*Arg:CN:CaM:eNOS:Hsp90:pAkt +
kcateNOS_H*Arg:CN:CaM:eNOS:Hsp90:pAkt + konpAktHsp90*CN:CaM:eNOS:Hsp90*pAkt ;

d(Arg:CN:CaM:eNOS:Hsp90:pAkt)/dt = konHsp90_CaMeNOS*Arg:CN:CaM:eNOS*pAkt:Hsp90 -
kdpAkt*Arg:CN:CaM:eNOS:Hsp90:pAkt + konCa2C*Ca*Ca*Arg:N:CaM:eNOS:Hsp90:pAkt -
koffCa2C*Arg:CN:CaM:eNOS:Hsp90:pAkt + konCa2N*Ca*Ca*Arg:C:CaM:eNOS:Hsp90:pAkt -
koffCa2N*Arg:CN:CaM:eNOS:Hsp90:pAkt + kon4eNOS_H*CN:CaM*Arg:eNOS:Hsp90:pAkt -
koffCaMeNOS_H*Arg:CN:CaM:eNOS:Hsp90:pAkt - koffpAktHsp90*Arg:CN:CaM:eNOS:Hsp90:pAkt -
koffHsp90_CaMeNOS*Arg:CN:CaM:eNOS:Hsp90:pAkt + koneNOSArg_H*Arg*CN:CaM:eNOS:Hsp90:pAkt -
koffeNOSArg_H*Arg:CN:CaM:eNOS:Hsp90:pAkt - kcateNOS_H*Arg:CN:CaM:eNOS:Hsp90:pAkt +
konpAktHsp90*Arg:CN:CaM:eNOS:Hsp90*pAkt ;

d(eNOS:Hsp90:Akt)/dt = kdpAkt*eNOS:Hsp90:pAkt + konHsp90eNOS*eNOS*Akt:Hsp90 -
kon2CeNOS_H*C:CaM*eNOS:Hsp90:Akt + koffCaMeNOS_H*C:CaM:eNOS:Hsp90:Akt -
kon2NeNOS_H*N:CaM*eNOS:Hsp90:Akt + koffCaMeNOS_H*N:CaM:eNOS:Hsp90:Akt -
kon4eNOS_H*CN:CaM*eNOS:Hsp90:Akt + koffCaMeNOS_H*CN:CaM:eNOS:Hsp90:Akt -
koffHsp90eNOS*eNOS:Hsp90:Akt - konbeNOSArg*Arg*eNOS:Hsp90:Akt + koffbeNOSArg*Arg:eNOS:Hsp90:Akt ;

d(Arg:eNOS:Hsp90:Akt)/dt = kdpAkt*Arg:eNOS:Hsp90:pAkt + konHsp90eNOS*Arg:eNOS*Akt:Hsp90 - kon2CeNOS_H*C:CaM*Arg:eNOS:Hsp90:Akt + koffCaMeNOS_H*Arg:C:CaM:eNOS:Hsp90:Akt - kon2NeNOS_H*N:CaM*Arg:eNOS:Hsp90:Akt + koffCaMeNOS_H*Arg:N:CaM:eNOS:Hsp90:Akt - kon4eNOS_H*CN:CaM*Arg:eNOS:Hsp90:Akt + koffCaMeNOS_H*Arg:CN:CaM:eNOS:Hsp90:Akt - koffHsp90eNOS*Arg:eNOS:Hsp90:Akt + konbeNOSArg*Arg*eNOS:Hsp90:Akt - koffbeNOSArg*Arg:eNOS:Hsp90:Akt ;

d(C:CaM:eNOS:Hsp90:Akt)/dt = kdpAkt*C:CaM:eNOS:Hsp90:pAkt + konHsp90_CaMeNOS*C:CaM:eNOS*Akt:Hsp90 - konCa2N*Ca*Ca*C:CaM:eNOS:Hsp90:Akt + koffCa2N*CN:CaM:eNOS:Hsp90:Akt + kon2CeNOS_H*C:CaM*eNOS:Hsp90:Akt - koffCaMeNOS_H*C:CaM:eNOS:Hsp90:Akt - koffHsp90_CaMeNOS*C:CaM:eNOS:Hsp90:Akt - koneNOSArg_H*Arg*C:CaM:eNOS:Hsp90:Akt + koffeNOSArg_H*Arg:C:CaM:eNOS:Hsp90:Akt + kcateNOS_H*Arg:C:CaM:eNOS:Hsp90:Akt ;

d(Arg:C:CaM:eNOS:Hsp90:Akt)/dt = kdpAkt*Arg:C:CaM:eNOS:Hsp90:pAkt + konHsp90_CaMeNOS*Arg:C:CaM:eNOS*Akt:Hsp90 - konCa2N*Ca*Ca*Arg:C:CaM:eNOS:Hsp90:Akt + koffCa2N*Arg:CN:CaM:eNOS:Hsp90:Akt + kon2CeNOS_H*C:CaM*Arg:eNOS:Hsp90:Akt - koffCaMeNOS_H*Arg:C:CaM:eNOS:Hsp90:Akt - koffHsp90_CaMeNOS*Arg:C:CaM:eNOS:Hsp90:Akt + koneNOSArg_H*Arg*C:CaM:eNOS:Hsp90:Akt - koffeNOSArg_H*Arg:C:CaM:eNOS:Hsp90:Akt - kcateNOS_H*Arg:C:CaM:eNOS:Hsp90:Akt ;

d(N:CaM:eNOS:Hsp90:Akt)/dt = kdpAkt*N:CaM:eNOS:Hsp90:pAkt + konHsp90_CaMeNOS*N:CaM:eNOS*Akt:Hsp90 - konCa2C*Ca*Ca*N:CaM:eNOS:Hsp90:Akt + koffCa2C*CN:CaM:eNOS:Hsp90:Akt + kon2NeNOS_H*N:CaM*eNOS:Hsp90:Akt - koffCaMeNOS_H*N:CaM:eNOS:Hsp90:Akt - koffHsp90_CaMeNOS*N:CaM:eNOS:Hsp90:Akt - koneNOSArg_H*Arg*N:CaM:eNOS:Hsp90:Akt + koffeNOSArg_H*Arg:N:CaM:eNOS:Hsp90:Akt + kcateNOS_H*Arg:N:CaM:eNOS:Hsp90:Akt ;

d(Arg:N:CaM:eNOS:Hsp90:Akt)/dt = kdpAkt*Arg:N:CaM:eNOS:Hsp90:pAkt + konHsp90_CaMeNOS*Arg:N:CaM:eNOS*Akt:Hsp90 - konCa2C*Ca*Ca*Arg:N:CaM:eNOS:Hsp90:Akt + koffCa2C*Arg:CN:CaM:eNOS:Hsp90:Akt + kon2NeNOS_H*N:CaM*Arg:eNOS:Hsp90:Akt - koffCaMeNOS_H*Arg:N:CaM:eNOS:Hsp90:Akt - koffHsp90_CaMeNOS*Arg:N:CaM:eNOS:Hsp90:Akt + koneNOSArg_H*Arg*N:CaM:eNOS:Hsp90:Akt - koffeNOSArg_H*Arg:N:CaM:eNOS:Hsp90:Akt - kcateNOS_H*Arg:N:CaM:eNOS:Hsp90:Akt ;

d(CN:CaM:eNOS:Hsp90:Akt)/dt = kdpAkt*CN:CaM:eNOS:Hsp90:pAkt + konHsp90_CaMeNOS*CN:CaM:eNOS*Akt:Hsp90 + konCa2C*Ca*Ca*N:CaM:eNOS:Hsp90:Akt - koffCa2C*CN:CaM:eNOS:Hsp90:Akt + konCa2N*Ca*Ca*C:CaM:eNOS:Hsp90:Akt - koffCa2N*CN:CaM:eNOS:Hsp90:Akt + kon4eNOS_H*CN:CaM*eNOS:Hsp90:Akt - koffCaMeNOS_H*CN:CaM:eNOS:Hsp90:Akt - koffHsp90_CaMeNOS*CN:CaM:eNOS:Hsp90:Akt - koneNOSArg_H*Arg*CN:CaM:eNOS:Hsp90:Akt + koffeNOSArg_H*Arg:CN:CaM:eNOS:Hsp90:Akt + kcateNOS_H*Arg:CN:CaM:eNOS:Hsp90:Akt ;

d(Arg:CN:CaM:eNOS:Hsp90:Akt)/dt = kdpAkt*Arg:CN:CaM:eNOS:Hsp90:pAkt + konHsp90_CaMeNOS*Arg:CN:CaM:eNOS*Akt:Hsp90 + konCa2C*Ca*Ca*Arg:N:CaM:eNOS:Hsp90:Akt - koffCa2C*Arg:CN:CaM:eNOS:Hsp90:Akt + konCa2N*Ca*Ca*Arg:C:CaM:eNOS:Hsp90:Akt - koffCa2N*Arg:CN:CaM:eNOS:Hsp90:Akt + kon4eNOS_H*CN:CaM*Arg:eNOS:Hsp90:Akt - koffCaMeNOS_H*Arg:CN:CaM:eNOS:Hsp90:Akt - koffHsp90_CaMeNOS*Arg:CN:CaM:eNOS:Hsp90:Akt + koneNOSArg_H*Arg*CN:CaM:eNOS:Hsp90:Akt - koffeNOSArg_H*Arg:CN:CaM:eNOS:Hsp90:Akt - kcateNOS_H*Arg:CN:CaM:eNOS:Hsp90:Akt ;

d(peNOS:pHsp90)/dt = koffCaMpeNOS_H*C:CaM:peNOS:pHsp90 + koffCaMpeNOS_H*N:CaM:peNOS:pHsp90 + koffCaMpeNOS_H*CN:CaM:peNOS:pHsp90 + konHsp90eNOS*peNOS*pHsp90 - kon2CpeNOS_H*C:CaM*peNOS:pHsp90 - kon2NpeNOS_H*N:CaM*peNOS:pHsp90 - kon4peNOS_H*CN:CaM*peNOS:pHsp90 - koffHsp90eNOS*peNOS:pHsp90 - kdpeNOS*peNOS:pHsp90 - konpeNOSArg_H*Arg*peNOS:pHsp90 + koffpeNOSArg_H*Arg:peNOS:pHsp90 + kcatpeNOS_H*Arg:peNOS:pHsp90 ;

d(Arg:peNOS:pHsp90)/dt = koffCaMpeNOS_H*Arg:C:CaM:peNOS:pHsp90 + koffCaMpeNOS_H*Arg:N:CaM:peNOS:pHsp90 + koffCaMpeNOS_H*Arg:CN:CaM:peNOS:pHsp90 + konHsp90eNOS*Arg:peNOS*pHsp90 - kon2CpeNOS_H*C:CaM*Arg:peNOS:pHsp90 - kon2NpeNOS_H*N:CaM*Arg:peNOS:pHsp90 - kon4peNOS_H*CN:CaM*Arg:peNOS:pHsp90 - koffHsp90eNOS*Arg:peNOS:pHsp90 - kdpeNOS*Arg:peNOS:pHsp90 + konpeNOSArg_H*Arg*peNOS:pHsp90 - koffpeNOSArg_H*Arg:peNOS:pHsp90 - kcatpeNOS_H*Arg:peNOS:pHsp90 ;

d(eNOS:Hsp90)/dt = koffpAktHsp90*eNOS:Hsp90:pAkt - kon2CeNOS_H*C:CaM*eNOS:Hsp90 + koffCaMeNOS_H*C:CaM:eNOS:Hsp90 - kon2NeNOS_H*N:CaM*eNOS:Hsp90 + koffCaMeNOS_H*N:CaM:eNOS:Hsp90 - kon4eNOS_H*CN:CaM*eNOS:Hsp90 + koffCaMeNOS_H*CN:CaM:eNOS:Hsp90 - konpAktHsp90*eNOS:Hsp90*pAkt - konbeNOSArg*Arg*eNOS:Hsp90 + koffbeNOSArg*Arg:eNOS:Hsp90 ;

d(Arg:eNOS:Hsp90)/dt = koffpAktHsp90*Arg:eNOS:Hsp90:pAkt - kon2CeNOS_H*C:CaM*Arg:eNOS:Hsp90 + koffCaMeNOS_H*Arg:C:CaM:eNOS:Hsp90 - kon2NeNOS_H*N:CaM*Arg:eNOS:Hsp90 + koffCaMeNOS_H*Arg:N:CaM:eNOS:Hsp90 - kon4eNOS_H*CN:CaM*Arg:eNOS:Hsp90 + koffCaMeNOS_H*Arg:CN:CaM:eNOS:Hsp90 - konpAktHsp90*Arg:eNOS:Hsp90*pAkt + konbeNOSArg*Arg*eNOS:Hsp90 - koffbeNOSArg*Arg:eNOS:Hsp90 ;

d(C:CaM:eNOS:Hsp90)/dt = koffpAktHsp90*C:CaM:eNOS:Hsp90:pAkt - konCa2N*Ca*Ca*C:CaM:eNOS:Hsp90 + koffCa2N*CN:CaM:eNOS:Hsp90 + kon2CeNOS_H*C:CaM*eNOS:Hsp90 - koffCaMeNOS_H*C:CaM:eNOS:Hsp90 - konpAktHsp90*C:CaM:eNOS:Hsp90*pAkt - koneNOSArg_H*Arg*C:CaM:eNOS:Hsp90 + koffeNOSArg_H*Arg:C:CaM:eNOS:Hsp90 + kcateNOS_H*Arg:C:CaM:eNOS:Hsp90 ;

d(Arg:C:CaM:eNOS:Hsp90)/dt = koffpAktHsp90*Arg:C:CaM:eNOS:Hsp90:pAkt - konCa2N*Ca*Ca*Arg:C:CaM:eNOS:Hsp90 + koffCa2N*Arg:CN:CaM:eNOS:Hsp90 + kon2CeNOS_H*C:CaM*Arg:eNOS:Hsp90 - koffCaMeNOS_H*Arg:C:CaM:eNOS:Hsp90 - konpAktHsp90*Arg:C:CaM:eNOS:Hsp90*pAkt + koneNOSArg_H*Arg*C:CaM:eNOS:Hsp90 - koffeNOSArg_H*Arg:C:CaM:eNOS:Hsp90 - kcateNOS_H*Arg:C:CaM:eNOS:Hsp90 ;

d(N:CaM:eNOS:Hsp90)/dt = koffpAktHsp90*N:CaM:eNOS:Hsp90:pAkt - konCa2C*Ca*Ca*N:CaM:eNOS:Hsp90 + koffCa2C*CN:CaM:eNOS:Hsp90 + kon2NeNOS_H*N:CaM*eNOS:Hsp90 - koffCaMeNOS_H*N:CaM:eNOS:Hsp90 - konpAktHsp90*N:CaM:eNOS:Hsp90*pAkt - koneNOSArg_H*Arg*N:CaM:eNOS:Hsp90 + koffeNOSArg_H*Arg:N:CaM:eNOS:Hsp90 + kcateNOS_H*Arg:N:CaM:eNOS:Hsp90 ;

d(Arg:N:CaM:eNOS:Hsp90)/dt = koffpAktHsp90*Arg:N:CaM:eNOS:Hsp90:pAkt - konCa2C*Ca*Ca*Arg:N:CaM:eNOS:Hsp90 + koffCa2C*Arg:CN:CaM:eNOS:Hsp90 + kon2NeNOS_H*N:CaM*Arg:eNOS:Hsp90 - koffCaMeNOS_H*Arg:N:CaM:eNOS:Hsp90 - konpAktHsp90*Arg:N:CaM:eNOS:Hsp90*pAkt + koneNOSArg_H*Arg*N:CaM:eNOS:Hsp90 - koffeNOSArg_H*Arg:N:CaM:eNOS:Hsp90 - kcateNOS_H*Arg:N:CaM:eNOS:Hsp90 ;

d(CN:CaM:eNOS:Hsp90)/dt = koffpAktHsp90*CN:CaM:eNOS:Hsp90:pAkt + konCa2C*Ca*Ca*N:CaM:eNOS:Hsp90 - koffCa2C*CN:CaM:eNOS:Hsp90 + konCa2N*Ca*Ca*C:CaM:eNOS:Hsp90 - koffCa2N*CN:CaM:eNOS:Hsp90 + kon4eNOS_H*CN:CaM*eNOS:Hsp90 - koffCaMeNOS_H*CN:CaM:eNOS:Hsp90 - konpAktHsp90*CN:CaM:eNOS:Hsp90*pAkt - koneNOSArg_H*Arg*CN:CaM:eNOS:Hsp90 + koffeNOSArg_H*Arg:CN:CaM:eNOS:Hsp90 + kcateNOS_H*Arg:CN:CaM:eNOS:Hsp90 ;

d(Arg:CN:CaM:eNOS:Hsp90)/dt = koffpAktHsp90*Arg:CN:CaM:eNOS:Hsp90:pAkt + konCa2C*Ca*Ca*Arg:N:CaM:eNOS:Hsp90 - koffCa2C*Arg:CN:CaM:eNOS:Hsp90 + konCa2N*Ca*Ca*Arg:C:CaM:eNOS:Hsp90 - koffCa2N*Arg:CN:CaM:eNOS:Hsp90 + kon4eNOS_H*CN:CaM*Arg:eNOS:Hsp90 - koffCaMeNOS_H*Arg:CN:CaM:eNOS:Hsp90 - konpAktHsp90*Arg:CN:CaM:eNOS:Hsp90*pAkt + koneNOSArg_H*Arg*CN:CaM:eNOS:Hsp90 - koffeNOSArg_H*Arg:CN:CaM:eNOS:Hsp90 - kcateNOS_H*Arg:CN:CaM:eNOS:Hsp90 ;

d(Akt:eNOS)/dt = koffHsp90eNOS*eNOS:pHsp90:Akt - kon2CpeNOS*C:CaM*Akt:eNOS + koffCaMpeNOS*C:CaM:eNOS:Akt - kon2NpeNOS*N:CaM*Akt:eNOS + koffCaMpeNOS*N:CaM:eNOS:Akt - kon4peNOS*CN:CaM*Akt:eNOS + koffCaMpeNOS*CN:CaM:eNOS:Hsp90:Akt - konHsp90eNOS*Akt:eNOS*pHsp90 - konbeNOSArg*Arg*Akt:eNOS + koffbeNOSArg*Arg:Akt:eNOS ;

d(Arg:Akt:eNOS)/dt = koffHsp90eNOS*Arg:eNOS:pHsp90:Akt - kon2CpeNOS*C:CaM*Arg:Akt:eNOS + koffCaMpeNOS*Arg:C:CaM:eNOS:Akt - kon2NpeNOS*N:CaM*Arg:Akt:eNOS + koffCaMpeNOS*Arg:N:CaM:eNOS:Akt - kon4peNOS*CN:CaM*Arg:Akt:eNOS + koffCaMpeNOS*Arg:CN:CaM:eNOS:Hsp90:Akt - konHsp90eNOS*Arg:Akt:eNOS*pHsp90 + konbeNOSArg*Arg*Akt:eNOS - koffbeNOSArg*Arg:Akt:eNOS ;

d(C:CaM:eNOS:Akt)/dt = koffHsp90_CaMeNOS*C:CaM:eNOS:pHsp90:Akt - konCa2N*Ca*Ca*C:CaM:eNOS:Akt + koffCa2N*CN:CaM:eNOS:Hsp90:Akt + kon2CpeNOS*C:CaM*Akt:eNOS - koffCaMpeNOS*C:CaM:eNOS:Akt - konHsp90_CaMeNOS*C:CaM:eNOS:Akt*pHsp90 - koneNOSArg*Arg*C:CaM:eNOS:Akt + koffeNOSArg*Arg:C:CaM:eNOS:Akt + kcateNOS*Arg:C:CaM:eNOS:Akt ;

d(Arg:C:CaM:eNOS:Akt)/dt = koffHsp90_CaMeNOS*Arg:C:CaM:eNOS:pHsp90:Akt - konCa2N*Ca*Ca*Arg:C:CaM:eNOS:Akt + koffCa2N*Arg:CN:CaM:eNOS:Hsp90:Akt + kon2CpeNOS*C:CaM*Arg:Akt:eNOS - koffCaMpeNOS*Arg:C:CaM:eNOS:Akt - konHsp90_CaMeNOS*Arg:C:CaM:eNOS:Akt*pHsp90 + koneNOSArg*Arg*C:CaM:eNOS:Akt - koffeNOSArg*Arg:C:CaM:eNOS:Akt - kcateNOS*Arg:C:CaM:eNOS:Akt ;

d(N:CaM:eNOS:Akt)/dt = koffHsp90_CaMeNOS*N:CaM:eNOS:pHsp90:Akt - konCa2C*Ca*Ca*N:CaM:eNOS:Akt + koffCa2C*CN:CaM:eNOS:Hsp90:Akt + kon2NpeNOS*N:CaM*Akt:eNOS - koffCaMpeNOS*N:CaM:eNOS:Akt -

konHsp90_CaMeNOS*N:CaM:eNOS:Akt*pHsp90 - koneNOSArg*Arg*N:CaM:eNOS:Akt + koffeNOSArg*Arg:N:CaM:eNOS:Akt + kcateNOS*Arg:N:CaM:eNOS:Akt ;

d(Arg:N:CaM:eNOS:Akt)/dt = koffHsp90_CaMeNOS*Arg:N:CaM:eNOS:pHsp90:Akt - konCa2C*Ca*Ca*Arg:N:CaM:eNOS:Akt + koffCa2C*Arg:CN:CaM:eNOS:Hsp90:Akt + kon2NpeNOS*N:CaM*Arg:Akt:eNOS - koffCaMpeNOS*Arg:N:CaM:eNOS:Akt - konHsp90_CaMeNOS*Arg:N:CaM:eNOS:Akt*pHsp90 + koneNOSArg*Arg*N:CaM:eNOS:Akt - koffeNOSArg*Arg:N:CaM:eNOS:Akt - kcateNOS*Arg:N:CaM:eNOS:Akt ;

d(CN:CaM:eNOS:Hsp90:Akt)/dt = koffHsp90_CaMeNOS*CN:CaM:eNOS:pHsp90:Akt + konCa2C*Ca*Ca*N:CaM:eNOS:Akt - koffCa2C*CN:CaM:eNOS:Hsp90:Akt + konCa2N*Ca*Ca*C:CaM:eNOS:Akt - koffCa2N*CN:CaM:eNOS:Hsp90:Akt + kon4peNOS*CN:CaM*Akt:eNOS - koffCaMpeNOS*CN:CaM:eNOS:Hsp90:Akt - konHsp90_CaMeNOS*CN:CaM:eNOS:Hsp90:Akt*pHsp90 - koneNOSArg*Arg*CN:CaM:eNOS:Hsp90:Akt + koffeNOSArg*Arg:CN:CaM:eNOS:Hsp90:Akt + kcateNOS*Arg:CN:CaM:eNOS:Hsp90:Akt ;

d(Arg:CN:CaM:eNOS:Hsp90:Akt)/dt = koffHsp90_CaMeNOS*Arg:CN:CaM:eNOS:pHsp90:Akt + konCa2C*Ca*Ca*Arg:N:CaM:eNOS:Akt - koffCa2C*Arg:CN:CaM:eNOS:Hsp90:Akt + konCa2N*Ca*Ca*Arg:C:CaM:eNOS:Akt - koffCa2N*Arg:CN:CaM:eNOS:Hsp90:Akt + kon4peNOS*CN:CaM*Arg:Akt:eNOS - koffCaMpeNOS*Arg:CN:CaM:eNOS:Hsp90:Akt - konHsp90_CaMeNOS*Arg:CN:CaM:eNOS:Hsp90:Akt*pHsp90 + koneNOSArg*Arg*CN:CaM:eNOS:Hsp90:Akt - koffeNOSArg*Arg:CN:CaM:eNOS:Hsp90:Akt - kcateNOS*Arg:CN:CaM:eNOS:Hsp90:Akt ;

species used as place holders (2)

d(Trash)/dt = kdegVEGF*TSP1:VEGF + kdegR2*R2cyt + kdegCD47*CD47cyt + kdegR2*VEGF:R2cyt + kdegCD47*TSP1:CD47cyt + kdegCD47*CD47:R2cyt + kdegR2*VEGF:pR2cyt + kdegpR2*VEGF:pR2cyt + kdegCD47*VEGF:R2.CD47cyt + kdegpR2CD47*VEGF:pR2:CD47cyt + kdegIP3*IP3 + kdegpeNOS*C:CaM:peNOS + kdegpeNOS*N:CaM:peNOS + kdegpeNOS*CN:CaM:peNOS + kdegpeNOS*peNOS ;

d(null)/dt = 0
