# Supplementary material for: Mathematical Model Predicts Effective Strategies to Inhibit VEGF-eNOS Signaling
Source: J Clin Med. 2020 Apr 26;9(5):1255. doi: 10.3390/jcm9051255 (PMC7287924; doi:10.3390/jcm9051255)
Supplement: Supplementary file 1 [file jcm-09-01255-s001.zip › jcm-727669-US-SI-supplementary-for conversion 2/jcm-727669-US-SI-supplementary-for conversion 2.docx]

**Figure S1.** Distribution of estimated parameters. Middle line: median parameter value among 19 estimations. Box: first to third quartile (25%–75%). Whiskers: minimum and maximum of the estimated values.

**Figure S2.** Sensitivity analysis to inform model fitting. Total sensitivity indices (S_ti_) of each model parameter with respect to five signaling species in a global sensitivity analysis using the extended Fourier Amplitude Sensitivity Test (eFAST) method. Simulations were run with the vascular endothelial growth factor (VEGF) concentration of 1.1 nM (30 ng/mL) and no Thrombospondin-1 (TSP1). Due to the large size of the model, we separated the model parameters to seven categories and conducted the sensitivity analyses within each group. These results were used to identify the influential but unknown parameters for model fitting. White squares: the parameter sensitivity index is not significant when compared to that of a random dummy variable (*p* > 0.05).

**Figure S3.** Sensitivity analyses to inform perturbation simulations. (**a**) Total sensitivity indices (S_ti_) of each model parameter with respect to five signaling species in a global sensitivity analysis using the eFAST method. Simulations were run without VEGF or TSP1. This set of results was used to identify the influential parameters for perturbation of the basal condition. (**b**) S_ti_ of each model parameter in simulation with 0.389 nM VEGF and 2.2 nM TSP1. This set of results was used to identify the influential parameters for perturbation of the VEGF-stimulated condition. White squares: the parameter sensitivity index is not significant when compared to that of a random dummy variable (*p* > 0.05). sGC, Soluble Guanylate Cyclase; eNOS, Endothelial Nitric Oxide Synthase.


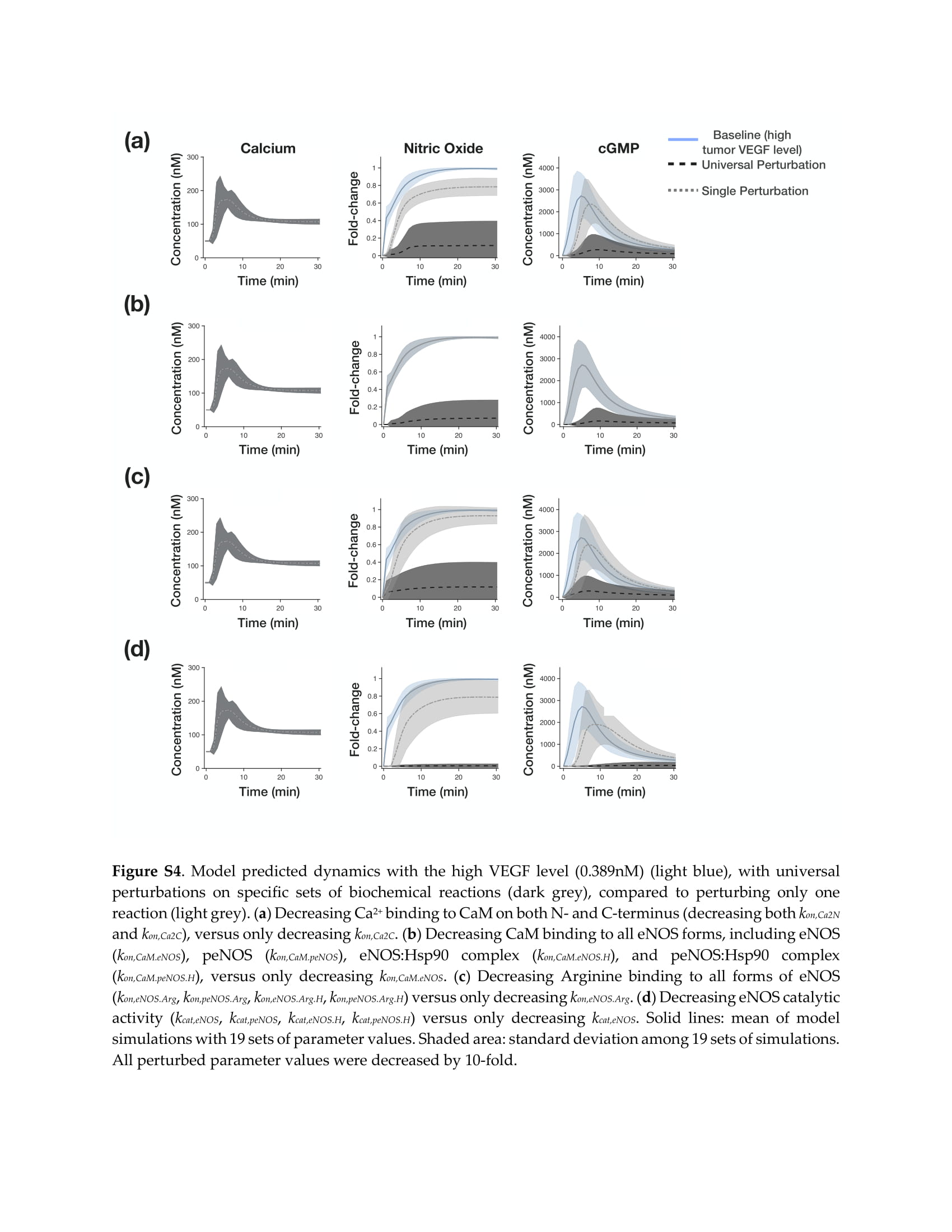


**Figure S4.** Model predicted dynamics with the high VEGF level (0.389 nM) (light blue), with universal perturbations on specific sets of biochemical reactions (dark grey), compared to perturbing only one reaction (light grey). (**a**) Decreasing Ca^2+^ binding to calmodulin (CaM) on both N- and C-terminus (decreasing both *k_on,Ca2N_* and *k_on,Ca2C_*), versus only decreasing *k_on,Ca2C_*. (**b**) Decreasing CaM binding to all endothelial nitric oxide synthase (eNOS) forms, including eNOS (*k_on,CaM.eNOS_*), phosphorylated eNOS (peNOS) (*k_on,CaM.peNOS_*), eNOS:Hsp90 complex (*k_on,CaM.eNOS.H_*), and peNOS:Hsp90 complex (*k_on,CaM.peNOS.H_*), versus only decreasing *k_on,CaM.eNOS_*. (**c**) Decreasing Arginine binding to all forms of eNOS (*k_on,eNOS.Arg_*, *k_on,peNOS.Arg_*, *k_on,eNOS.Arg.H_*, *k_on,peNOS.Arg.H_*) versus only decreasing *k_on,eNOS.Arg_*. (**d**) Decreasing eNOS catalytic activity (*k_cat,eNOS_*, *k_cat,peNOS_*, *k_cat,eNOS.H_*, *k_cat,peNOS.H_*) versus only decreasing *k_cat,eNOS_*. Solid lines: mean of model simulations with 19 sets of parameter values. Shaded area: standard deviation among 19 sets of simulations. All perturbed parameter values were decreased by 10-fold. cGMP, Cyclic Guanosine Monophosphate.

**Table S1.** List of model parameters.

| **Index** | **Parameter** | **Value *** | **Unit** | **Source** |
| --- | --- | --- | --- | --- |
| **Receptor Module** | | | | |
| 1 | k_on,TSP1.VEGF_ | 0.03 | nM^−1^ min^−1^ | as assumed in [1] |
| 2 | k_off,TSP1.VEGF_ | 0.3 | min^−1^ | as assumed in [1] |
| 3 | k_deg,VEGF.TSP1_ | 0.0116 | min^−1^ | as assumed in [1] |
| 4 | k_on,VEGF.R2_ | 0.6 | nM^−1^ min^−1^ | assumed¶ |
| 5 | k_off,VEGF.R2_ | 0.06 | min^−1^ | calculated based on a k_d_ of 100 pm [2] |
| 6 | k_on,TSP1.CD47_ | 0.03 | nM^−1^ min^−1^ | as assumed in [1] |
| 7 | k_off,TSP1.CD47_ | 0.0003 | min^−1^ | calculated based on a k_d_ of 10 pm[3] |
| 8 | k_on,R2.CD47_ | 0.0011 | nM^−1^ min^−1^ | as assumed in [4] |
| 9 | k_off,R2.CD47_ | 0.06 | min^−1^ | as assumed in [4] |
| 10 | k_inter,R2_ (R2, R2:CD47) | 0.0426  (0.0421–0.0429) | min^−1^ | estimated in receptor module (see Appendix)¶¶ |
| 11 | k_inter,pR2_ (pR2, R2:CD47) | 4.7682  (3.0321–5.8339) | min^−1^ | estimated in receptor module (see Appendix) |
| 12 | k_inter,CD47_ (only CD47) | 0.5692  (0.2987–0.8321) | min^−1^ | estimated in full model |
| 13 | k_inter,R2.CD47_ | 0.0085 | min^−1^ | estimated (Appendix) |
| 14 | k_inter,pR2.CD47_ | 23.8410 | min^−1^ | estimated (Appendix) |
| 15 | k_inter,TSP1bd_ (any complex containing TSP1) | 0.5692 | min^−1^ | assume same as k_inter,CD47_ |
| 16 | k_p,R2_ | 0.6  (0.3251–0.8473) | min^−1^ | estimated in full model |
| 17 | k_dp,R2_ | 0.06 | min^−1^ | tuned† |
| 18 | k_dp,R2.CD47bd_ | 0.01 | min^−1^ | tuned |
| 19 | k_syn,R2_ | 0.0482  (0.0478–0.0484) | nM *min^−1^ | estimated in receptor module (see Appendix) |
| 20 | k_syn,CD47_ | 0.0899  (1.382e-4–0.1) | nM *min^−1^ | estimated in receptor module (see Appendix) |
| 21 | k_deg,R2_ | 0.0126  (0.0125–0.0210) | min^−1^ | estimated in receptor module (see Appendix) |
| 22 | k_deg,pR2_ | 0.2219  (0.1309–0.3697) | min^−1^ | estimated in full model |
| 23 | k_deg,pR2.CD47_ | 0.0168  (0.0059–0.0212) | min^−1^ | estimated in receptor module (see Appendix) |
| 24 | k_deg,CD47_ | 0.0063 | min^−1^ | tuned |
| 25 | k_deg,R2TSP1bd_ | 0.0168 | min^−1^ | assume same as #23 |
| **Src/Akt/Hsp90 Module** | | | | |
| 26 | k_on,Src.pR2_ | 0.06  (0.0296–0.3001) | nM^−1^ min^−1^ | estimated in full model |
| 27 | k_off,Src.pR2_ | 25 | min^−1^ | tuned |
| 28 | k_p,Src_ | 20  (10.9511–29.1037) | min^−1^ | estimated in full model |
| 29 | k_dp,Src_ | 0.4  (3080–0.5572) | min^−1^ | estimated in full model |
| 30 | k_on,Src.Hsp90_ | 0.5 | nM^−1^ min^−1^ | assumed |
| 31 | k_off,Src.hsp90_ | 0.5 | min^−1^ | tuned |
| 32 | k_p,Hsp90_ | 30 | min^−1^ | assumed |
| 33 | k_dp,Hsp90_ | 0.01 | min^−1^ | tuned |
| 34 | k_on,Src.Akt_ | 0.06 | nM^−1^ min^−1^ | assumed |
| 35 | k_off,Src.Akt_ | 20 | min^−1^ | tuned |
| 36 | k_p,Akt_ | 3  (1.7288–4.5689) | min^−1^ | estimated in full model |
| 37 | k_dp,Akt_ | 0.1  (0.0407–0.1444) | min^−1^ | estimated in full model |
| **Calcium Module** | | | | |
| 38 | k_p,PLCg_ | 10  (6.3173–15.4105) | min^−1^ | estimated in full model |
| 39 | k_dp,PLCg_ | 0.3 | min^−1^ | assumed |
| 40 | K_M,PIP2PLCg_ | 193.5858 | nM | [5] |
| 41 | n_IP3_ | 1 | Unitless; coefficient | the hill number for generation of IP3 by PLCγ; assumed |
| 42 | k_cat,PLCg_ | 0.3000  (0.0935–0.4366) | min^−1^ | estimated in full model |
| 43 | k_deg,IP3_ | 0.0200  (0.0039–0.0376) | min^−1^ | estimated in full model |
| 44 | k_syn,PIP2_ | 10 | nM * min^−1^ | tuned |
| 45 | I_CRAC_ | 1.50E-09  (9.2142 × 10^−10^–3.0694 × 10^−9^) | min^−1^ | estimated in full model |
| 46 | K_CRAC_ | 2492.5441  (938.1226–2492.5441) | nM | estimated in fitting calcium module (Appendix) |
| 47 | t_stim_ | 1 | min | tuned |
| 48 | K_M,PLCgR2_ | 8000 | nM | [5] |
| 49 | n_CRAC_ | 0.6580 | Unitless; coefficient | the hill number for the steady-state CRAC channel activation; estimated (lsqnonlin) |
| 50 | I_IP3R_ | 0.3910  (0.2638–0.5892) | min^−1^ | estimated in full model |
| 51 | K_M,IP3R_ | 20000  (10292–33382) | nM | baseline assumed based on [6]; estimated in full model |
| 52 | I_PMCA_ | 220418.4750 | min^−1^ | calculated for calcium homeostasis (see Appendix)§ |
| 53 | K_M,PMCA_ | 260 | nM | assumed based on [7] |
| 54 | Ca_ext_ | 2000000 | nM | assumed |
| 55 | I_SERCA_ | 435320.5900  (3.8909 × 10^5–^7.6429 × 10^5^) | min^−1^ | estimated in calcium module (see Appendix) |
| 56 | k_leak,ER_ | 6.80E-09 | min^−1^ | calculated for homeostasis |
| 57 | K_M,SERCA_ | 150 | nM | [5] |
| 58 | K_i,Ca_ | 1000 | nM | [5] |
| 59 | CSQN | 15000000 | nM | [5] |
| 60 | K_CSQN_ | 800000 | nM | [5] |
| **eNOS Module** | | | | |
| 61 | k_on,Ca2C_ | 0.24 | nM^−1^ min^−1^ | [8] |
| 62 | k_off,Ca2C_ | 555 | min^−1^ | [8] |
| 63 | k_on,Ca2N_ | 6 | nM^−1^ min^−1^ | [8] |
| 64 | k_off,Ca2N_ | 45000 | min^−1^ | [8] |
| 65 | k_on,2NeNOS_ | 0.0081 | nM^−1^ min^−1^ | [9] |
| 66 | k_on,2CeNOS_ | 0.078 | nM^−1^ min^−1^ | [9] |
| 67 | k_on,4eNOS_ | 0.078 | nM^−1^ min^−1^ | [9] |
| 68 | k_off,CaMeNOS_ | 0.6 | min^−1^ | [9] |
| 69 | k_on,2N.eNOS.H_ | 0.0325 | nM^−1^ min^−1^ | calculated based on [10] |
| 70 | k_on,2C.eNOS.H_ | 0.3133 | nM^−1^ min^−1^ | calculated based on [10] |
| 71 | k_on,4.eNOS.H_ | 0.3133 | nM^−1^ min^−1^ | calculated based on [10] |
| 72 | k_off,CaM.eNOS.H_ | 0.6000 | min^−1^ | [9] |
| 73 | k_on,2N.peNOS_ | 0.0675 | nM^−1^ min^−1^ | calculated based on [10] |
| 74 | k_on,2C.peNOS_ | 0.65 | nM^−1^ min^−1^ | calculated based on [10] |
| 75 | k_on,4.peNOS_ | 0.65 | nM^−1^ min^−1^ | calculated based on [10] |
| 76 | k_off,CaMpeNOS_ | 0.6 | min^−1^ | [9] |
| 77 | k_on,2N.peNOS.H_ | 0.1157 | nM^−1^ min^−1^ | calculated based on [10] |
| 78 | k_on,2CpeNOS.H_ | 1.1143 | nM^−1^ min^−1^ | calculated based on [10] |
| 79 | k_on,4peNOS.H_ | 1.1143 | nM^−1^ min^−1^ | calculated based on [10] |
| 80 | k_off,CaM.peNOS.H_ | 0.6 | min^−1^ | calculated based on [10] |
| 81 | k_on,Akt.eNOS_ | 0.02 | nM^−1^ min^−1^ | assumed |
| 82 | k_off,Akt.eNOS_ | 4.0380  (3.7831–4.8589) | min^−1^ | estimated in eNOS module (See Appendix) |
| 83 | k_on,pAkt.Hsp90_ | 0.5 | nM^−1^ min^−1^ | assumed |
| 84 | k_off,pAkt.Hsp90_ | 10.2826  (2.0842–10.7935) | min^−1^ | estimated in eNOS module (see Appendix) |
| 85 | k_on,Hsp90.eNOS_ | 0.5 | nM^−1^ min^−1^ | assumed |
| 86 | k_off,Hsp90.eNOS_ | 5.7327  (0.8854–47.0715) | min^−1^ | estimated in eNOS module (see Appendix) |
| 87 | k_on,Hsp90.CaMeNOS_ | 0.5 | nM^−1^ min^−1^ | assumed |
| 88 | k_off,Hsp90.CaMeNOS_ | 10.0976  (1.0167–10.0976) | min^−1^ | estimated in eNOS module (see Appendix) |
| 89 | k_cat,pAkt_ | 3 | min^−1^ | tuned |
| 90 | k_cat,pAk.H_ | 10 | min^−1^ | tuned |
| 91 | k_dp,eNOS_ | 2.8  (1.7897–5.0371) | min^−1^ | estimated in full model |
| 92 | k_deg,peNOS_ | 0.001 | min^−1^ | tuned |
| 93 | k_on,beNOS.Arg_ | 0.048 | nM^−1^ min^−1^ | [11] |
| 94 | k_off,beNOS.Arg_ | 96 | min^−1^ | [11] |
| 95 | k_on,eNOS.Arg_ | 0.048 | nM^−1^ min^−1^ | [11] |
| 96 | k_off,eNOS.Arg_ | 2.9023  (0.3844–10.1851) | min^−1^ | estimated in eNOS module (see Appendix) |
| 97 | k_on,peNOS.Arg_ | 0.048 | nM^−1^ min^−1^ | [11] |
| 98 | k_off,peNOS.Arg_ | 4.9891  (0.4062–33.1763) | min^−1^ | estimated in eNOS module (see Appendix) |
| 99 | k_on,eNOS.Arg.H_ | 0.048 | nM^−1^ min^−1^ | [11] |
| 100 | k_off,eNOS.Arg.H_ | 6.987  (0.7341–55.1524) | min^−1^ | estimated in eNOS module (see Appendix) |
| 101 | k_on,peNOS.Arg.H_ | 0.048 | nM^−1^ min^−1^ | [11] |
| 102 | k_off,peNOS.Arg.H_ | 10.8397  (1.1183–85.2601) | min^−1^ | estimated in eNOS module (see Appendix) |
| 103 | k_cat,eNOS_ | 1.49  (1.0729–8.6044) | min^−1^ | estimated in eNOS module (see Appendix) |
| 104 | k_cat,peNOS_ | 2.682  (2.1118–16.7032) | min^−1^ | estimated in eNOS module (see Appendix) |
| 105 | k_cat,eNOS.H_ | 3.132  (1.8966–17.1657) | min^−1^ | estimated in eNOS module (see Appendix) |
| 106 | k_cat,peNOS.H_ | 4.22  (4.0505–24.2466) | min^−1^ | estimated in eNOS module (see Appendix) |
| **sGC Module** | | | | |
| 107 | k_clearNO_ | 10  (5.4822–14.5019) | min^−1^ | baseline assumed based on [12,13]; estimated in full model |
| 108 | k_on,NO.sGC_ | 18 | nM^−1^ min^−1^ | [14] |
| 109 | k_off,NO.sGC_ | 360 | min^−1^ | [14] |
| 110 | k_a.sGC_ | 1680 | min^−1^ | [14] |
| 111 | k_d,sGC_ | 1680 | min^−1^ | [14] |
| 112 | k_on,NO.NOGC_ | 0.24 | nM^−1^ min^−1^ | [14] |
| 113 | k_off,NO.NOGC_ | 60000 | min^−1^ | [14] |
| 114 | k_f,NOGC.NO_ | 120000 | nM^−1^ min^−1^ | [14] |
| 115 | k_r,NOGC.NO_ | 0.108 | min^−1^ | [14] |
| 116 | k_f.GC.NO_ | 24 | nM^−1^ min^−1^ | [14] |
| 117 | k_r.GC.NO_ | 0.044 | min^−1^ | [14] |
| 118 | k_f6_ | 60 | min^−1^ | [14] |
| 119 | k_r6_ | 0.06 | min^−1^ | [14] |
| 120 | k_cat,sGC_ | 31.5 | min^−1^ | tuned |
| 121 | k_on,cGMP.PDE_ | 0.001 | nM^−1^ min^−1^ | [14] |
| 122 | k_off,cGMP.PDE_ | 7.752 | min^−1^ | [14] |
| 123 | k_a,PDE_ | 18 | min^−1^ | [14] |
| 124 | k_d,PDE_ | 7.2 | min^−1^ | [14] |
| 125 | K_M.PDE1_ | 4000 | nM | [14] |
| 126 | k_cat,PDE_ | 0.2925  (0.1602–0.4325) | min^−1^ | estimated in full model |
| 127 | K_M,PDE_ | 1000 | nM | [14] |
| **Geometric Parameters** | | | | |
| 128 | Vol_cyto_ | 9.12 × 10^−13^ | L | [7,15] |
| 129 | Vol_ext_ | 5.00 × 10^−4^ | L | assumed |
| 130 | Vol_ER_ | 3.35 × 10^−13^ | L | [7] |
| **Initial Condition** | | | | |
| 131 | TSP1_0 | 0–2.2 | nM | Based on experimentally used exogeneous concentrations |
| 132 | VEGF_0 | 0–1.1 | nM | Based on range of measured tissue concentrations as compiled in [16] and experimentally used exogenous concentrations[17] |
| 133 | R2_0 | 8.971 | nM | [18] |
| 134 | CD47_0 | 199.336 | nM | measured‡ |
| 135 | Src_0 | 344  (188.5609–517.2423) | nM | estimated in full model |
| 136 | Ca_0 | 50 | nM | assumed |
| 137 | Ca_store__0 | 2000000 | nM | assumed |
| 138 | CaM_0 | 30  (13.4411–41.0572) | nM | baseline assumed based on [19]; estimated in full model |
| 139 | eNOS_0 | 100  (46.5482–158.0576) | nM | baseline assumed based on [20];  estimated in full model |
| 140 | Arg_0 | 100000 | nM | assumed based on [21] |
| 141 | Hsp90_0 | 500  (234.0886–687.3168) | nM | estimated in full model |
| 142 | Akt_0 | 800  (493.1294–1182.4231) | nM | baseline assumed based on [22]; estimated in full model |
| 143 | sGC_b_0 | 10 | nM | tuned |
| 144 | GTP_0 | 500000 | nM | Assumed based on [23] |
| 145 | PIP2_0 | 200000  (1.4409 × 10^5–^3.0279 × 10^5^) | nM | baseline assumed based on [24]; estimated in full model |
| 146 | PLCg_0 | 500 | nM | assumed |
| 147 | Istim0 | 19990.3745 | nM/min | Initial flux through CRAC, calculated for homeostasis§§ |
| 148 | PDE_0 | 500000 | nM | tuned |

*, Value used in model. Range indicates range of estimated parameter values obtained during fitting. ¶, assumed: no data for reference unless indicated. ¶¶, estimated in module: parameters were previously estimated during model development before full model fitting. The baseline model takes the best fit value from estimated values. †, tuned: manually adjusted. ‡, measured: species level quantified using flow cytometry (see Appendix: Supplemental Text: Receptor Quantification). §, I_PMCA_ is calculated by the equation I_PMCA_ *(Ca_0)^1^.^4^/(K_M,PMCA_^1^.^4^ + Ca_0^1^.^4^) + Istim0 = 0, to balance the initial calcium flux across cell membrane. §§, Istim0 is calculated by setting the initial CRAC influx to be zero, through equation ((((((Vol_ext_/Vol_cyto_) *Ca_ext)-Ca_0)*I_CRAC_)*((K_CRAC_^n_CRAC_)/((K_CRAC_^n_CRAC_) + (Ca_store__0^n_CRAC_))))/t_stim_)-(Istim0/t_stim_) = 0. IP_3_, Inositol 1,4,5-Trisphosphate; PLCγ, phospholipase C-γ; CRAC, Calcium Release-Activated Channels; eNOS, Endothelial Nitric Oxide Synthase.

**Table S2.** List of Perturbations.

| **Altered Parameter (Direction)** | **Function** | **Reduce Basal Activity (0.003 nM VEGF)** | | | **Reduce 0.389 nM VEGF-Stimulated Signaling** | | | **Existing Therapeutic Compounds** |
| --- | --- | --- | --- | --- | --- | --- | --- | --- |
|  |  | **Ca^2+^** | **NO** | **cGMP** | **Ca^2+^** | **NO** | **cGMP** |  |
| k_p.R2_ (-) | Phosphorylation of R2 | / | / | / | Yes | Yes | No | TKIs (Sorafenib, Sunitinib, etc.) [25] |
| k_on,Src.pR2_ (-) | Binding of Src with pR2 | / | / | / | No | Yes | No |  |
| k_p,Src_ (-) | Activation of Src | / | / | / | No | Yes | No | Apatinib (TKI) [26], Dasatinib, Saracatinib, Bosutinib, KX01 [27] |
| k_dp,Src_ (+) | Deactivation of Src | / | / | / | No | Yes | No |  |
| k_p,Hsp90_ (-) | Activation of Hsp90 | / | / | / | No | Yes | No | Geldanamycin and its derived analogs, radicicol [28] |
| k_p,PLC𝜸_ (-) | Phosphorylation of PLC𝜸 | / | / | / | Yes | No | No | Genistein [29] |
| k_cat,PLC𝜸_ (-) | Catalytic activity of PLC𝜸 | / | / | / | Yes | No | No | U73122 and compounds under investigation [30] |
| k_deg,IP3_ (+) | Degradation of IP_3_ | / | / | / | Yes | No | No |  |
| I_IP3 R_ (-) | Flux through IP_3_R | / | / | / | Yes | No | No | 2-APB, caffeine [31], CAI [32] |
| K_M,IP3R_ (+) | Michaelis-Menten constant of IP_3_R | / | / | / | Yes | No | No | Heparin [31] |
| K_M,PMCA_ (-) | Michaelis-Menten constant of PMCA | / | / | / | Yes | Yes | No | Caloxins [33] |
| I_CRAC_ (-) | Flux through CRAC | Yes | Yes | No | Yes | No | No | CAI [32] |
| K_CRAC_ (-) | Michaelis-Menten constant of CRAC | Yes | Yes | No | Yes | No | No |  |
| Ca_ext_ (-) | Extracelluar Ca^2+^ | Yes | Yes | No | Yes | No | No |  |
| I_SERCA_  (+) | Flux through SERCA | Yes | Yes | Yes | Yes | No | No |  |
| K_M,SERCA_ (-) | Michaelis-Menten constant of SERCA | Yes | Yes | Yes | Yes | No | No | Thapsigargin [34,35] |
| k_off,Ca2C_ (+) | Dissociation of Ca^2+^ from CaM C-terminus | No | Yes | Yes | No | Yes | No |  |
| k_off,CaMeNOS_ (+) | Dissociation of CaM and eNOS | No | Yes | Yes | No | Yes | No |  |
| k_on,Hsp90.CaMeNOS_ (-) | Binding of Hsp90 with CaM:eNOS | / | / | / | No | Yes | No |  |
| k_off,Hsp90.CaMeNOS_ (+) | Dissociation of Hsp90 and CaM:eNOS | / | / | / | No | Yes | No |  |
| k_cat,eNOS_ (-) | Catalytic activity of eNOS | No | Yes | Yes | No | Yes | No |  |
| k_cat,eNOS.H_ (-) | Catalytic activity of Hsp90-bound eNOS | / | / | / | No | Yes | Yes |  |
| k_clear,NO_ (+) | Clearance rate of NO | No | Yes | Yes | No | Yes | Yes |  |
| k_off.NO.sGC_ (+) | Dissociation of NO from sGC distal heme; k_-1_ in Halvey model | / | / | / | No | No | Yes | ODQ, NS2028 [36] |
| k_a.sGC_ (-) | Activation of sGC; k_2_ in Halvey model | / | / | / | No | No | Yes |  |
| k_off.NO.NOGC_ (-) | Dissociation of NO from sGC proximal heme; k_-3_ in Halvey model | / | / | / | No | No | Yes |  |
| k_f.NOGC.NO_ (+) | Dissociation of NO from NO-bound sGC distal heme; k4 in Halvey model | / | / | / | No | No | Yes |  |
| k_r.NOGC.NO_ (-) | Association of NO to NO-bound sGC on distal heme; k-4 in Halvey model | / | / | / | No | No | Yes |  |
| k_cat.sGC_ (-) | Catalytic activity of sGC | No | No | Yes | No | No | Yes |  |
| k_on,cGMP.PDE_ (+) | Binding of cGMP with PDE | / | / | / | No | No | Yes |  |
| k_off.cGMP.PDE_ (-) | Dissociation of cGMP and PDE | / | / | / | No | No | Yes |  |
| k_a.PDE_ (+) | Activation of the first state of PDE | No | No | Yes | No | No | Yes |  |
| k_d,PDE_ (-) | Deactivation of the first state PDE | No | No | Yes | No | No | Yes |  |
| k_a2.PDE_ (+) | Activation of the second state of PDE | No | No | Yes | No | No | Yes |  |
| k_d2,PDE_ (-) | Deactivation of the second state PDE | No | No | Yes | No | No | Yes |  |
| K_M,PDE_ (-) | Michaelis-Menten constant of PDE activity | / | / | / | No | No | Yes |  |
| k_cat,PDE_ (+) | Catalytic activity of PDE | No | No | Yes | No | No | Yes |  |
| Src (-) | Src expression level | / | / | / | No | Yes | Yes |  |
| CaM (-) | CaM expression level | No | Yes | Yes | No | Yes | Yes | trifluoperazine [37] |
| eNOS (-) | eNOS expression level | No | Yes | Yes | No | Yes | Yes |  |
| Hsp90 (-) | Hsp90 expression level | / | / | / | No | Yes | No |  |
| PIP2 (-) | PIP2 level | / | / | / | Yes | No | No | Triazole-based compounds [38] |
| sGC (-) | sGC expression level | No | No | Yes | No | No | Yes |  |
| GTP (-) | GTP level | No | No | Yes | No | No | Yes |  |

(+) sign: parameter was increased using the left panel Hill function in Figure 3; (-) sign: parameter was decreased using the right panel Hill function in Figure 3; forward slash (/): Not applicable. The parameter was not used as perturbation target, because it was not an influential parameter in the corresponding VEGF condition. R2, Vascular Endothelial Growth Factor Receptor 2; TKI, tyrosine kinase inhibitor; pR2, phosphorylated R2; Src, proto-oncogene protein kinase Src; Hsp90, Heat Shock Protein 90; PLCγ, Phospholipase C, Gamma; IP_3_, Inositol 1,4,5-Trisphosphate; IP3R, IP3 Receptor; 2-APB, **2**-Aminoethoxydiphenyl borate; CAI, Carboxyamidotriazole; PMCA, Plasma Membrane Ca^2+^ Atpase; CRAC, Calcium Release-Activated Channels; SERCA, Sarco/Endoplasmic Reticulum Ca^2+^-Atpase; CaM, Calmodulin; eNOS, Endothelial Nitric Oxide Synthase; NO, Nitric Oxide; sGC, Soluble Guanylate Cyclase; ODQ, 1H-(1,2,4) oxadiazolo (4,3-a) quinoxalin-1-one; cGMP, Cyclic Guanosine Monophosphate; PDE, Phosphodiesterase; PIP_2_, Phosphatidylinositol 4,5-Bisphosphate; GTP, Guanosine Triphosphate. CSQN, Calsequestrin.

References

1. Li, D.; Finley, S.D. The impact of tumor receptor heterogeneity on the response to anti-angiogenic cancer treatment. *Integr. Biol.* **2018,** *10*, 253–269, doi:10.1039/C8IB00019K.
2. Keyt, B.A.; Berleau, L.T.; Nguyen, H.V.; Chen, H.; Heinsohn, H.; Vandlen, R.; Ferrara, N. The Carboxyl-terminal Domain (111165) of Vascular Endothelial Growth Factor Is Critical for Its Mitogenic Potency. *J. Biol. Chem.* **1996,** *271*, 7788–7795, doi:10.1074/jbc.271.13.7788.
3. Isenberg, J.S.; Ridnour, L.A.; Dimitry, J.; Frazier, W.A.; Wink, D.A.; Roberts, D.D. CD47 Is Necessary for Inhibition of Nitric Oxide-stimulated Vascular Cell Responses by Thrombospondin-1. *J. Biol. Chem.* **2006,** *281*, 26069–26080, doi:10.1074/jbc.M605040200.
4. Rohrs, J.A.; Sulistio, C.D.; Finley, S.D. Predictive model of thrombospondin-1 and vascular endothelial growth factor in breast tumor tissue. *Npj. Syst. Biol. Appl.* **2016,** *2*, 16030, doi:10.1038/npjsba.2016.30.
5. Bazzazi, H.; Isenberg, J.S.; Popel, A.S. Inhibition of VEGFR2 activation and its downstream signaling to ERK1/2 and calcium by Thrombospondin-1 (TSP1): In silico investigation. *Front. Physiol.* **2017,** *8*, doi:10.3389/fphys.2017.00048.
6. Carter, T.D.; Ogden, D. Kinetics of Ca2+ release by InsP3 in pig single aortic endothelial cells: Evidence for an inhibitory role of cytosolic Ca2+ in regulating hormonally evoked Ca2+ spikes. *J. Physiol.* **1997,** *504*, 17–33, doi:10.1111/j.1469-7793.1997.00017.x.
7. Silva, H.S.; Kapela, A.; Tsoukias, N.M. A mathematical model of plasma membrane electrophysiology and calcium dynamics in vascular endothelial cells. *Am. J. Physiol. Cell Physiol.* **2007,** *293*, 277–293, doi:10.1152/ajpcell.00542.2006.
8. Pepke, S.; Kinzer-Ursem, T.; Mihalas, S.; Kennedy, M.B. A dynamic model of interactions of Ca2+, calmodulin, and catalytic subunits of Ca2+/calmodulin-dependent protein kinase ii. *Plos Comput. Biol.* **2010,** *6*, doi:10.1371/journal.pcbi.1000675.
9. Romano, D.R.; Pharris, M.C.; Patel, N.M.; Kinzer-Ursem, T.L. Competitive tuning: Competition’s role in setting the frequency-dependence of Ca2+-dependent proteins. *Plos Comput. Biol.* **2017,** *13*, doi:10.1371/journal.pcbi.1005820.
10. Chen, Y.; Jiang, B.; Zhuang, Y.; Peng, H.; Chen, W. Differential effects of heat shock protein 90 and serine 1179 phosphorylation on endothelial nitric oxide synthase activity and on its cofactors. *PloS One* **2017,** *12*, doi:10.1371/journal.pone.0179978.
11. Berka, V.; Tsai, A. Characterization of Interactions among the Heme Center, Tetrahydrobiopterin, and l -Arginine Binding Sites of Ferric eNOS using Imidazole, Cyanide, and Nitric Oxide as Probes ^†^. *Biochemistry* **2000,** *39*, 9373–9383, doi:10.1021/bi992769y.
12. Sriram, K.; Laughlin, J.G.; Rangamani, P.; Tartakovsky, D.M. Shear-induced nitric oxide production by endothelial cells. *Biophys. J.* **2016,** *111*, 208–221, doi:10.1016/j.bpj.2016.05.034.
13. Villanueva, C.; Giulivi, C. Subcellular and cellular locations of nitric-oxide synthase isoforms as determinants of health and disease. *Free Radic. Biol. Med.* **2010,** *49*, 307–316, doi:10.1016/j.freeradbiomed.2010.04.004.
14. Halvey, E.J.; Vernon, J.; Roy, B.; Garthwaite, J. Mechanisms of Activity-dependent Plasticity in Cellular Nitric Oxide-cGMP Signaling. *J. Biol. Chem.* **2009,** *284*, 25630–25641, doi:10.1074/jbc.M109.030338.
15. Rubin, D.B.; Drab, E.A.; Bauer, K.D. Endothelial cell subpopulations in vitro: Cell volume, cell cycle, and radiosensitivity. *J. Appl Physiol* **1989,** *67*, 1585–1590, doi:10.1152/jappl.1989.67.4.1585.
16. Finley, S.D.; Dhar, M.; Popel, A.S. Compartment model predicts VEGF secretion and investigates the effects of VEGF trap in tumor-bearing mice. *Front. Oncol.* **2013,** *3*, doi:10.3389/fonc.2013.00196.
17. Isenberg, J.S.; Ridnour, L.A.; Perruccio, E.M.; Espey, M.G.; Wink, D.A.; Roberts, D.D. Thrombospondin-1 inhibits endothelial cell responses to nitric oxide in a cGMP-dependent manner. *Proc. Natl. Acad. Sci. USA* **2005,** *102*, 13141–13146, doi:10.1073/pnas.0502977102.
18. Chen, S.; Guo, X.; Imarenezor, O.; Imoukhuede, P.I. Quantification of VEGFRs, NRP1, and PDGFRs on Endothelial Cells and Fibroblasts Reveals Serum, Intra-Family Ligand, and Cross-Family Ligand Regulation. *Cel. Mol. Bioeng.* **2015,** *8*, 383–403, doi:10.1007/s12195-015-0411-x.
19. Piazza, M.; Dieckmann, T.; Guillemette, J.G. Structural studies of a complex between endothelial nitric oxide synthase and calmodulin at physiological calcium concentration. *Biochemistry* **2016,** *55*, 5962–5971, doi:10.1021/acs.biochem.6b00821.
20. Chen, K.; Popel, A.S. Theoretical analysis of biochemical pathways of nitric oxide release from vascular endothelial cells. *Free Radic. Biol. Med.* **2006,** *41*, 668–680, doi:10.1016/j.freeradbiomed.2006.05.009.
21. Shin, S.; Mohan, S.; Fung, H.-L. Intracellular L-arginine concentration does not determine NO production in endothelial cells: Implications on the “L-arginine paradox.” *Biochem. Biophys. Res. Commun.* **2011,** *414*, 660–663, doi:10.1016/j.bbrc.2011.09.112.
22. Song, M.; Finley, S.D. Mechanistic insight into activation of MAPK signaling by pro-angiogenic factors. *Bmc. Syst. Biol.* **2018,** *12*, doi:10.1186/s12918-018-0668-5.
23. Traut, T.W. Physiological concentrations of purines and pyrimidines. *Mol. Cell Biochem.* **1994,** *140*, 1–22, doi:10.1007/BF00928361.
24. Faehling, M.; Kroll, J.; Föhr, K.J.; Fellbrich, G.; Mayr, U.; Trischler, G.; Waltenberger, J. Essential role of calcium in vascular endothelial growth factor A-induced signaling: Mechanism of the antiangiogenic effect of carboxyamidotriazole. *Faseb. J.* **2002,** *16*, 1805–1807, doi:10.1096/fj.01-0938fje.
25. Aprile, G.; Bonotto, M.; Ongaro, E.; Pozzo, C.; Giuliani, F. Critical Appraisal of Ramucirumab (IMC-1121B) for Cancer Treatment: From Benchside to Clinical Use. *Drugs* **2013,** *73*, 2003–2015, doi:10.1007/s40265-013-0154-8.
26. Tian, S.; Quan, H.; Xie, C.; Guo, H.; Lü, F.; Xu, Y.; Li, J.; Lou, L. YN968D1 is a novel and selective inhibitor of vascular endothelial growth factor receptor-2 tyrosine kinase with potent activity in vitro and in vivo. *Cancer Sci.* **2011,** *102*, 1374–1380, doi:10.1111/j.1349-7006.2011.01939.x.
27. Creedon, H.; Brunton, V.G. Src Kinase Inhibitors: Promising Cancer Therapeutics? *CRO* **2012,** *17*, doi:10.1615/CritRevOncog.v17.i2.20.
28. Neckers, L.; Workman, P. Hsp90 molecular chaperone inhibitors: Are we there yet? *Clin. Cancer Res.* **2012,** *18*, 64–76, doi:10.1158/1078-0432.CCR-11-1000.
29. Papapetropoulos, A.; García-Cardeña, G.; Madri, J.A.; Sessa, W.C. Nitric oxide production contributes to the angiogenic properties of vascular endothelial growth factor in human endothelial cells. *J. Clin. Invest.* **1997,** *100*, 3131–3139, doi:10.1172/JCI119868.
30. Huang, W.; Barrett, M.; Hajicek, N.; Hicks, S.; Harden, T.K.; Sondek, J.; Zhang, Q. Small Molecule Inhibitors of Phospholipase C from a Novel High-throughput Screen. *J. Biol. Chem.* **2013,** *288*, 5840–5848, doi:10.1074/jbc.M112.422501.
31. Saleem, H.; Tovey, S.C.; Molinski, T.F.; Taylor, C.W. Interactions of antagonists with subtypes of inositol 1,4,5-trisphosphate (IP3) receptor. *Br. J. Pharm.* **2014,** *171*, 3298–3312, doi:10.1111/bph.12685.
32. Comprehensive Cancer Information. Available online: https://www.cancer.gov/ (accessed 2 March 2020).
33. Pande, J.; Szewczyk, M.M.; Grover, A.K. Allosteric inhibitors of plasma membrane Ca2+ pumps: Invention and applications of caloxins. *World J. Biol. Chem.* **2011**, *2*, 39–47, doi:10.4331/wjbc.v2.i3.39.
34. Sehgal, P.; Szalai, P.; Olesen, C.; Praetorius, H.A.; Nissen, P.; Christensen, S.B.; Engedal, N.; Møller, J.V. Inhibition of the sarco/endoplasmic reticulum (ER) Ca2+-ATPase by thapsigargin analogs induces cell death via ER Ca2+ depletion and the unfolded protein response. *J. Biol. Chem.* **2017,** *292*, doi:10.1074/jbc.M117.796920.
35. Toyoshima, C.; Nomura, H. Structural changes in the calcium pump accompanying the dissociation of calcium. *Nature* **2002,** *418*, 605–611, doi:10.1038/nature00944.
36. Vijayaraghavan, J.; Kramp, K.; Harris, M.E.; van den Akker, F. Inhibition of Soluble Guanylyl Cyclase by Small Molecules Targeted to the Catalytic Domain. *Febs. Lett.* **2016,** *590*, 3669–3680, doi:10.1002/1873-3468.12427.
37. Vandonselaar, M.; Hickie, R.A.; Quail, W.; Delbaere, L.T.J. Trifluoperazine-induced conformational change in Ca^2+^ -calmodulin. *Nat. Struct. Mol. Biol.* **1994,** *1*, 795–801, doi:10.1038/nsb1194-795.
38. Gorai, S.; Bagdi, P.R.; Borah, R.; Paul, D.; Santra, M.K.; Khan, A.T.; Manna, D. Insights into the inhibitory mechanism of triazole-based small molecules on phosphatidylinositol-4,5-bisphosphate binding pleckstrin homology domain. *Biochem. Biophys. Rep.* **2015,** *2*, 75–86, doi:10.1016/j.bbrep.2015.05.007.

| 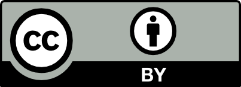 | © 2020 by the authors. Licensee MDPI, Basel, Switzerland. This article is an open access article distributed under the terms and conditions of the Creative Commons Attribution (CC BY) license (http://creativecommons.org/licenses/by/4.0/). |
| --- | --- |
